# Adverse Events in Nonsurgical Facial Aesthetic Procedures: A Systematic Review and Meta‐Analysis

**DOI:** 10.1111/odi.70109

**Published:** 2025-10-05

**Authors:** Vitória Maria Sousa Cruz, Sebastião Silvério Sousa‐Neto, Caique Mariano Pedroso, Maria Eduarda Pérez‐de‐Oliveira, Ana Gabriela Costa Normando, Helen Kaline Farias Bezerra, Rafael Tomaz Gomes, Ana Carolina Prado‐Ribeiro, Danyel Elias da Cruz Perez, Pablo Agustin Vargas, Alan Roger Santos‐Silva

**Affiliations:** ^1^ Oral Diagnosis Department, Piracicaba Dental School University of Campinas (UNICAMP) São Paulo Brazil; ^2^ Private Practice São Paulo Brazil; ^3^ Dental Oncology Service, Instituto Do Câncer Do Estado de São Paulo (ICESP) Faculdade de Medicina da Universidade de São Paulo (FMUSP) São Paulo Brazil; ^4^ Department of Clinical and Preventive Dentistry Universidade Federal de Pernambuco (UFPE) Recife Brazil

**Keywords:** adverse events, botulinum toxins, hyaluronic acid fillers, meta‐analysis, systematic review, thread lifting

## Abstract

**Objective:**

To determine the prevalence and clinical profile of treatment‐related adverse events (TRAE) associated with: (1) botulinum toxin in the upper face; (2) hyaluronic acid fillers in the lower face and nasolabial folds; and (3) nonsurgical facelifts with absorbable threads.

**Methods:**

Searches were conducted in five electronic databases and gray literature. Proportion meta‐analyses were performed to estimate the prevalence of TRAE for each type of aesthetic procedure.

**Results:**

42 studies were included. TRAEs were reported in 34.8% of included patients. The highest TRAEs prevalence was associated with hyaluronic acid fillers (63.0%, 95% CI: 35%–84%), followed by nonsurgical facelifts with absorbable threads (20.0%, 95% CI: 8%–41%) and botulinum toxin in the upper face (18.0%, 95% CI: 10%–32%). The most frequently reported TRAEs were swelling (22.4%) and pain (19.0%) for hyaluronic acid fillers; pain (31.5%) and bruising (23.4%) for nonsurgical facelifts with threads; and headache for botulinum toxins. Hyaluronic acid fillers accounted for 67.3% of all TRAEs (*n* = 3729/4971).

**Conclusions:**

The overall prevalence of adverse events was low for botulinum toxin and thread lifts, but high for hyaluronic acid fillers. Future studies should report all adverse events, expected and not expected, detailing the number of patients affected, total events, onset and recovery times, severity, and provider category.

## 1 Introduction

Nonsurgical aesthetic procedures (NSAP), also known as minimally invasive aesthetic treatments, are elective healthcare interventions designed to improve appearance without surgical incisions or general anesthesia (Triana et al. 2024). A commonly used facial rejuvenation approach combines botulinum toxin‐A (BoNT‐A) in the upper face (forehead, glabellar, and lateral canthal lines) with hyaluronic acid (HA) fillers in the lower face (chin, marionette lines, and lips) and nasolabial folds, correcting both dynamic wrinkles and volume loss (Coleman and Carruthers 2006). Nonsurgical thread lifting has also become popular as a less invasive alternative to facelift surgery, often yielding longer lasting results than BoNT‐A or HA fillers alone, with lower morbidity and faster recovery (Ziade et al. 2025).

Despite their popularity, demand for these procedures has raised safety concerns (Hong et al. 2024b, 2024a). Although treatment‐related adverse events (TRAEs) are generally mild and self‐limiting (Chadha et al. 2024; Taylor et al. 2019; Alimohammadi et al. 2024), standardized terminology and classifications, management protocols, and reporting criteria remain lacking. The TRAEs profile depends on procedure type, injection site, injector skill, and material used and may include localized reactions, vascular occlusion, or systemic effects, with variable onset (Hong et al. 2024b; Sito et al. 2019).

Anatomical differences across facial regions further influence risks (Hong et al. 2024a; Sethi et al. 2021). Thus, we focused on regions commonly treated with HA fillers and BoNT‐A to reduce bias. This systematic review aims to synthesize and analyze evidence on the prevalence and profile of TRAEs associated with BoNT‐A in the upper face, HA fillers in the lower face (including lips) and nasolabial folds, and nonsurgical facelifts with absorbable threads. The focused question of this systematic review was: In patients undergoing minimally invasive aesthetic procedures with botulinum toxin‐A in the upper face, hyaluronic acid fillers in the lower face/nasolabial folds, or nonsurgical facelifts with absorbable threads, what is the prevalence and clinical profile of treatment‐related adverse events?

## 2 Material and Methods

### 2.1 Protocol

The present systematic review was conducted using the Preferred Reporting Items for Systematic Reviews and Meta‐Analysis (PRISMA) checklist (Table S1) as the reporting template (Page et al. 2021). The study was previously registered with the International Prospective Register of Systematic Reviews in Health and Social Care (PROSPERO, National Institute for Health Research, UK) under the number CRD42022321104.

### 2.2 Eligibility Criteria

**FIGURE 1:**
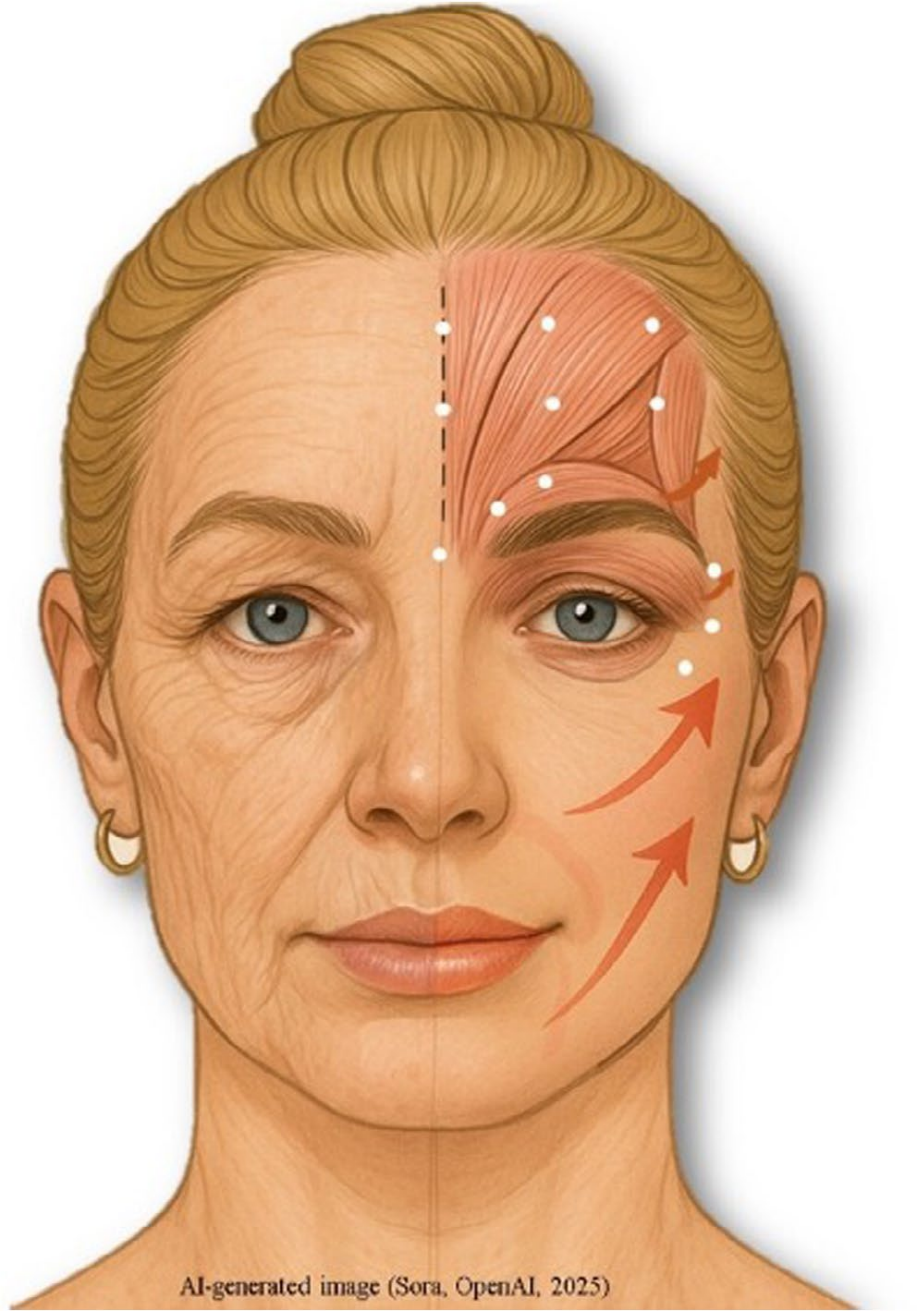
Illustration representing the regions per procedure considered for the studies included in this review. Orange arrows—Indicate facelifts performed with absorbable threads in various regions of the face, excluding the neck. White dots—Indicate the most common points of botulinum toxin application in three upper facial regions included in the study: the external canthal area (crow's feet), the glabellar/procerus complex, and the forehead/frontal region. Pink shading—Highlights the labial region, chin, nasolabial folds and marionette lines. Studies were included in which hyaluronic acid‐based dermal fillers were applied.

The PICO framework guided the review question: Population—adult individuals undergoing nonsurgical aesthetic procedures in the facial area; Intervention—botulinum toxin‐A in the upper face, hyaluronic acid fillers in the lower face/nasolabial folds, or nonsurgical facelifts with absorbable threads; Comparison—none; Outcomes—prevalence and clinical profile of treatment‐related adverse events. Figure 1 illustrates the anatomical regions, materials, and procedures considered for inclusion in the study.

We used the National Cancer Institute's (2025) definition of adverse event, characterized as “An undesired effect of a drug or other type of treatment, ranging from mild to severe and can be life‐threatening.” We adapted this definition in our study to any undesirable sign, symptom, or condition with a plausible causal relationship to the aesthetic procedure, regardless of whether it was expected or unexpected. TRAEs could be reported either by physicians (objective signs such as erythema, swelling, or bruising) or by patients (subjective symptoms such as pain, itching, or burning sensation).

A predefined list of adverse events was not established to avoid underreporting. Health events without a causal link to the intervention (e.g., ovarian cancer) were not classified as TRAEs and were therefore excluded. Both objectively assessed and subjectively reported adverse events were considered. Signs such as erythema, swelling, or bruising could be recorded by physicians or patients, while symptoms such as pain, itching, or a burning sensation were primarily self‐reported.

We excluded the following studies: (1) that involved procedures for non‐aesthetic reasons, non‐facial or lip locations, or procedures other than BoNT‐A, HA fillers, or nonsurgical facelift with absorbable threads; (2) that did not investigate the frequency of TRAEs related to the specified procedures; (3) studies in which TRAEs could not be extracted due to clustering with other aesthetic procedures; (4) involving thread‐lifting procedures that extended to the neck; (5) on injectable fillers that did not use hyaluronic acid; (6) when fillers did not include the marionette lines, chin, lips, or nasolabial folds; (7) when toxin did not target the procerus, frontal region/forehead, or lateral canthal lines; (8) historical cohort studies in which TRAEs were part of the inclusion criterion; (9) that combined multiple minimally invasive facial aesthetic procedures within the same treatment period, thereby preventing isolated analysis of individual interventions; (10) reviews, case series/reports, protocols, short communications, personal opinions, letters, conference abstracts, book chapters, and in vitro or in vivo animal studies; (11) full‐text copy was not available; (12) published in languages other than English, Spanish, or Portuguese; (13) duplicated sample.

### 2.3 Information Sources and Search Strategy

Searches were conducted on December 12, 2024, in PubMed, Scopus, EMBASE, Web of Science, and LILACS without time restrictions. Gray literature was also searched via Google Scholar, ProQuest (Table S2), and references of included studies.

### 2.4 Selection Process

We used a two‐step duplicate removal process: records were first exported to EndNote (V.M.S.C.) for initial duplicate removal, then imported into Rayyan for manual deduplication. Two reviewers (V.M.S.C. and S.S.S.‐N.) screened titles and abstracts in Rayyan; potentially relevant articles underwent full‐text review. Disagreements were resolved by consulting a third reviewer (A.R.S.‐S.). A fourth author (C.M.P.) screened references of eligible studies.

### 2.5 Data Items and Collection Process

We extracted the following variables: authorship/year; study design; number of patients/procedures; treated site; follow‐up duration; number of patients with TRAEs; total TRAEs; TRAE description/intensity; onset and resolution times; permanent sequelae; patient sex and age range; and, when available, TRAE management. Data were collected separately for HA fillers, thread lifting, and BoNT‐A in specific facial areas.

### 2.6 Risk of Bias Assessment

Two reviewers (V.M.S.C. and S.S.S.‐N.) independently assessed methodological quality and risk of bias using Joanna Briggs Institute tools for cohort studies, non‐randomized trials, and randomized trials (Aromataris and Munn 2020). The checklists covered domains such as sequence generation, blinding, confounding control, outcome measurement, and statistical analysis. Discrepancies were resolved by discussion or with a third reviewer (C.M.P.). Risk of bias was categorized as high (≤ 49% “yes”), moderate (50%–69%), or low (≥ 70%). Risk of bias graphs were generated in Review Manager 5.4.

### 2.7 Effect Measures

The primary outcome was the prevalence of TRAEs associated with each type of procedure. Secondary outcomes included: (i) the clinical profile of TRAEs (type, intensity, onset, duration, resolution, and sequelae); (ii) management strategies for TRAEs.

Continuous variables (e.g., mean age and number of procedures per patient) were extracted and summarized descriptively but not pooled, as no continuous outcome was common across studies. Dichotomous data (e.g., number of patients with ≥ 1 TRAE out of total treated patients) were used to calculate prevalence. As no continuous outcomes were common across included studies, weighted mean differences were not used.

### 2.8 Synthesis Methods

Studies reporting both total patients and those with TRAEs were included in the quantitative synthesis, grouped by intervention. Pooled prevalence meta‐analysis was performed using the inverse variance method with Freeman–Tukey double arcsine transformation. A random‐effects model with the DerSimonian–Laird estimator was applied to account for between‐study variance. Heterogeneity was assessed with the Q test and *I* ^2^ statistic, with thresholds of 25%, 50%, and 75% considered low, moderate, and high heterogeneity, respectively. Subgroup analyses were performed according to: (i) type of procedure (HA fillers, BoNT‐A injections, nonsurgical facelifts with absorbable threads), and (ii) timing of adverse events (early‐onset within hours to days vs. late‐onset within weeks to years).

TRAEs were also classified according to their time of onset. We adapted the classification proposed by Urdiales‐Gálvez et al. (2018), which defined immediate (< 24 h), early (24 h–4 weeks), and late (> 4 weeks) complications. However, most of the included studies did not distinguish between immediate and early events, nor did they specify the onset separately for each symptom. Therefore, events were grouped into two categories: “early,” referring to complications that arise within hours to days, and “late,” referring to those that arise within weeks to years. When the exact timing was not reported, classification was based on the plausibility of the description (e.g., swelling, erythema, and pain—early; nodules, pigmentation changes, and hair migration—late).

### 2.9 Certainty Assessment

The certainty of the evidence was assessed using the GRADE approach (Guyatt et al. 2011), considering risk of bias, inconsistency, indirectness, imprecision, and other factors. The evidence was rated as high, moderate, low, or very low, and a GRADE profile was generated with GRADEpro.

## 3 Results

### 3.1 Search and Study Selection

The electronic search identified 9328 records in databases and 742 additional studies in gray literature, resulting in a total of 10,070 references. After the duplicate removal, 5469 references remained, with 6491 from the main databases and 742 from the gray literature. In the screening phase, 392 articles were selected for assessing the full text. From these, 41 studies met the eligibility criteria. In addition, one study was selected from a manual search of reference lists of the included studies, resulting in a total of 42 studies included in the qualitative synthesis and 31 in the quantitative synthesis. The selection process is illustrated in Figure S1.

### 3.2 Study Characteristics

The 42 included studies were published between 2000 and 2024, comprising 1 prospective cohort study, 10 retrospective cohort studies, and 31 clinical trials. There were 21 articles (50%; *n* = 2009 patients); investigating HA fillers, 13 (31%; *n* = 6254 patients); upper face BoNT‐A, and 8 (19%; *n* = 590 patients); nonsurgical facelift. In total, 8853 patients were evaluated, ranging 10–2785 patients among the studies (Tables S4–S6). The patients' age ranged 18‐86 years. Females (*n* = 7784; 87.9%) outnumbered males (*n* = 823; 9.3%), with a male‐to‐female ratio of 1:9.5. There were 4971 TRAEs after aesthetic intervention reported in 2758/7932 patients (34.8%). The follow‐up of the patients ranged 0–44 months. The procedure with most reported TRAEs was the HA fillers (*n* = 3729; 67.3%), followed by BoNT‐A (*n* = 1448; 26.1%) and nonsurgical facelift (*n* = 368; 6.6%).

### 3.3 Risk of Bias

All randomized trials were classified as low risk of bias. Among non‐randomized trials, one (11.1%) had moderate risk and eight (88.9%) low risk, mainly due to lack of a control group, incomplete follow‐up, or unclear statistical methods. For cohort studies, three (27.3%) had low risk, three (27.3%) moderate risk, and five (45.4%) high risk, often due to confounding, incomplete follow‐up, or inadequate statistical analysis. Detailed assessments are in Tables S7–S9. Figure S2 presents the risk of bias assessment for each included study using the Cochrane risk of bias tool.

### 3.4 Results of Individual Studies

Eleven studies did not report the number of patients who experienced TRAEs, providing only the total number of events. The remaining 31 studies (*n* = 7932) reported the number of patients treated, the number of patients with TRAEs, and the total number of TRAEs. For HA fillers, a TRAE rate of 54.2% was observed (967 out of 1783 treated patients), with a total of 3609 TRAEs reported. Regarding BoNT‐A, 1717 out of 5862 patients (29.3%) experienced at least one TRAE. For nonsurgical facelift, 141 TRAEs were reported in 74 of the 287 treated patients (25.8%).

In some studies, the number of patients with TRAEs exceeded the number of treatment‐related events (Li, Chiang, et al. 2023; Lowe et al. 2005; Kerscher et al. 2015; Cox et al. 2023; Solish et al. 2023; Chadha et al. 2024; Fagien et al. 2024), as some publications reported any health event experienced by participants, even when not directly associated with the aesthetic procedure. In these cases, it was not possible to count the number of patients who reported TRAEs, excluding patients who reported events unrelated to treatment.

#### 3.4.1 HA Fillers

The complementary characteristics according to each procedure are detailed in Table S10. Most of the patients were female (*n* = 1658; 82.5%), with ages ranging 18–79 years; however, patients' sex was not reported in three studies (Marcus et al. 2022; Ince et al. 2024; Li, Chiang, et al. 2023).

Among six studies evaluating HA fillers in the lips that reported both the number of participants and the number of TRAEs (Yazdanparast et al. 2017; Taylor et al. 2019; Hilton et al. 2023; Ehlinger‐David et al. 2023; Ince et al. 2024; Müller et al. 2024), 233 out of 358 patients (65.1%) reported at least one event (Table S6). Regarding the nasolabial fold, six studies (Nikolis et al. 2021; Li, Li, et al. 2023; Xie et al. 2023; Alimohammadi et al. 2024; Lheritier et al. 2024; Shao et al. 2024) described 483 TRAEs among 673 treated patients (71.8%). For the chin, three studies (Marcus et al. 2022; Liao et al. 2025; Nikolis et al. 2024) reported that 227 out of 419 patients (54.2%) experienced TRAEs. Additionally, one study evaluating HA filler application in the lips, marionette lines, and nasolabial folds observed that 74 out of 153 treated patients (48.4%) reported TRAEs (David et al. 2023).

The five most common TRAEs associated with the injection of HA fillers were swelling (*n* = 835; 22.4%), pain (*n* = 709; 19%), bruising/hematoma (*n* = 636; 17.1%), erythema/redness (*n* = 561; 15%); and tenderness (*n* = 337; 9%). Most TRAEs were mild to moderate in intensity (1382), while only 81 reported TRAEs were serious. However, eight studies (*n* = 2259) didn't classify the TRAEs according to their intensity (Müller et al. 2024; Shao et al. 2024; Nikolis et al. 2024; Liao et al. 2025; Li, Chiang, et al. 2023; Ince et al. 2024; Ehlinger‐David et al. 2023; David et al. 2023). No permanent sequel was reported. The duration of events ranged 1–122 days. Many TRAEs were resolved spontaneously. Hyaluronidase was used only in a few cases (Alimohammadi et al. 2024; Marcus et al. 2022; Nikolis et al. 2021; Yazdanparast et al. 2017). Furthermore, one study reported the occurrence of two cases of herpes simplex (Hilton et al. 2023) and another (Samadi et al. 2025) reported the occurrence of mild dry skin in one patient.

**FIGURE 2:**
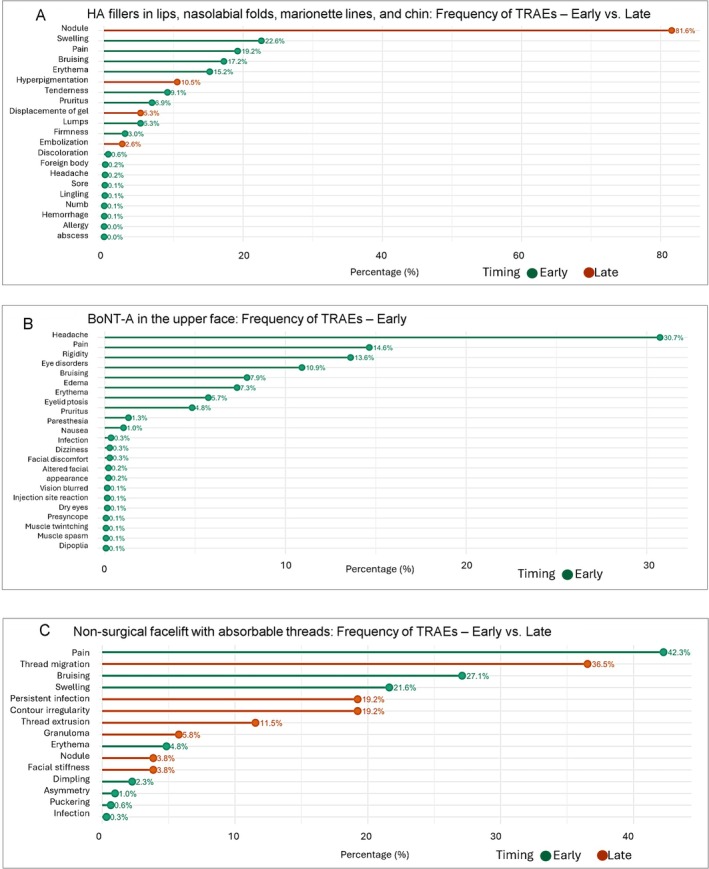
Prevalence of early (in green) and late (in orange) TRAEs after HA fillers in lips, lower face and nasolabial folds (a), upper face treatment with BoNT‐A (b), and nonsurgical facelift with absorbable threads (c).

Figure 2a shows the early and late TRAEs and the percentage relative to the total effects of each class. It can be observed that the late TRAEs were represented by nodules, hyperpigmentation, displacement of gel, and embolization.

#### 3.4.2 BoNT‐A

The complementary characteristics according to each procedure are detailed in Table S11. There were 6254 treated patients. Most of the patients were female (*n* = 5587, 89.3%), aged between 21 and 86 years. DaxibotulinumtoxinA (*n* = 2833; 45.3%) was the main material used; followed by OnabotulinumtoxinA (*n* = 1250; 20%); AbobotulinumtoxinA (*n* = 150; 2.4%); PrabotulinumtoxinA (*n* = 841; 13.4%); Botulinum Toxin A (*n* = 826 patients; 13.2%); Botulinum Toxin Type A—MBA‐P01 (*n* = 249; 4%); and IncobotulinumtoxinA (*n* = 105 procedures; 1.7%).

The total number of TRAEs was 1447 (Table S5). The five main TRAEs were headache (*n* = 445; 30.7%), pain (*n* = 208; 14.4%), rigidity (*n* = 197; 13.6%), eye disorders (*n* = 158; 10.9%), and bruising/ecchymosis (*n* = 114; 7.9%). No permanent or late sequel has been reported (Figure 2b). Eyelid ptosis appeared in the first 14 days after treatment in two studies (Fagien et al. 2024; Lowe et al. 2005). The time to onset of TRAEs was reported in only three studies (Lowe et al. 2005; Chadha et al. 2024; Fagien et al. 2024). Of these, two studies (Lowe et al. 2005; Fagien et al. 2024) reported only the time to onset of eyelid ptosis (14–15 days), while the time to improvement was reported in only one study (Fagien et al. 2024) as 67 days. Another study (Chadha et al. 2024) reported that TRAEs appeared on the same day as the treatment, with healing occurring within 4 days. Two additional studies (Carruthers et al. 2003; Ascher et al. 2004), which did not report the time to onset of TRAEs, provided data on the time to improvement, stating that headaches resolved within a few hours, changes in facial appearance within 2–3 weeks, and ecchymosis within up to 1 month.

#### 3.4.3 Nonsurgical Facelift With Absorbable Threads

The complementary characteristics according to each procedure are detailed in Table S12. There were 590 patients. Most of the patients were female (*n* = 539 patients; 91.4%), aged between 19 and 72 years. The types of materials used were polydioxanone thread (*n* = 547 patients; 92.7%) and poly‐lactic‐caprolactone thread (*n* = 50; 7.3%).

There were 74/287 patients presenting a total of 141 TRAEs (Table S9). The five common TRAEs associated with nonsurgical facelift techniques were pain (*n* = 116; 31.5%), followed by bruising (*n* = 86; 23.4%), swelling (*n* = 67; 18.2%), thread migration or exposure (*n* = 19; 5.2%), and dimpling (*n* = 17; 4.6%). No permanent sequel was reported.

The time to onset of TRAEs was not reported in any study. Many studies (*n* = 27) did not report on the management of TRAEs (*n* = 2045; 41.1%). In 87 occurrences, the TRAEs were resolved spontaneously. However, systemic antibiotics were used to treat infections (*n* = 2); nodule formations (*n* = 2), while nonsteroidal anti‐inflammatory drugs were applied in five cases of pain. Corticosteroids were used to treat granuloma formation (*n* = 2). Thread extraction was treated with removal in 19 cases. Two cases of dimpling were treated with massage and subcision, while another 10 cases were treated with manual massage alone. In seven cases of erythema (3) or ecchymosis (4), the use of cold compress without pressure was advised. Figure 2c presents early and late‐onset TRAE associated with nonsurgical facelifts using absorbable threads. Among late‐onset effects, thread migration, persistent infection, contour irregularity, and thread extrusion were the most frequently reported.

### 3.5 Results of Data Synthesis

#### 3.5.1 Dermal Fillers

**FIGURE 3:**
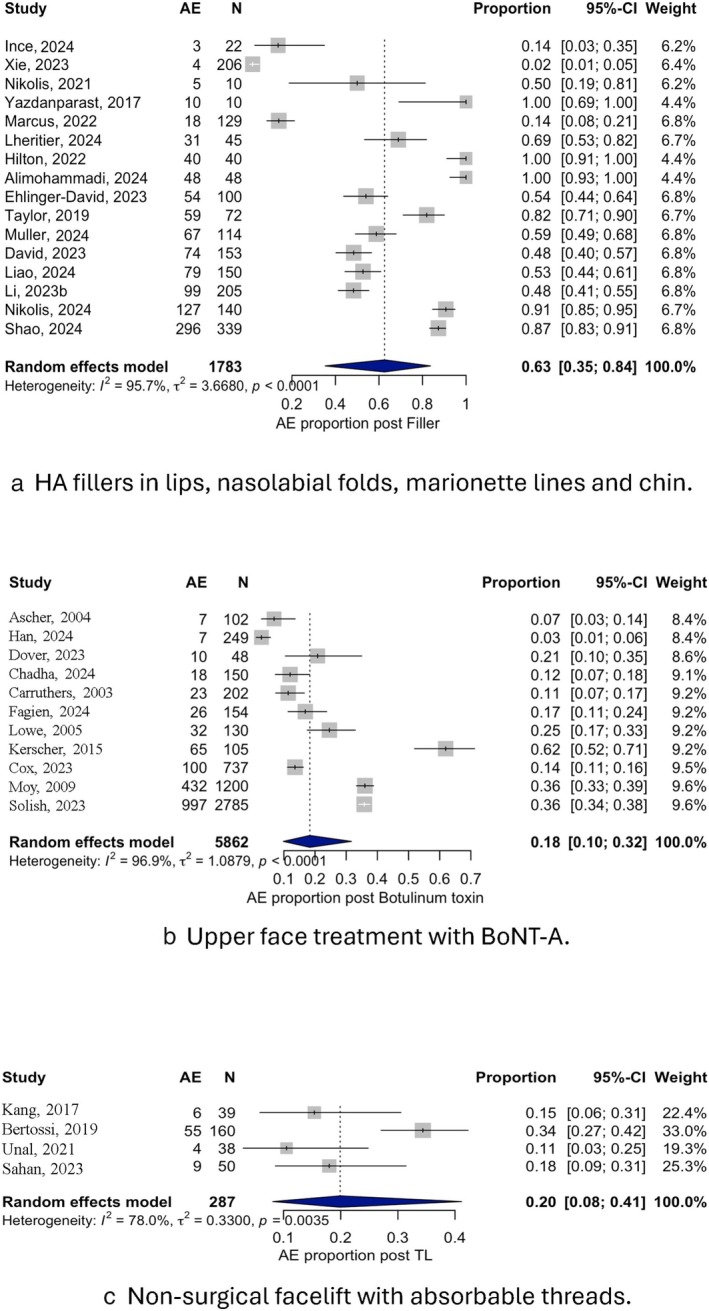
Prevalence of TRAEs after HA fillers in lips, lower face, and nasolabial folds (a), upper face treatment with BoNT‐A (b), and nonsurgical facelift with absorbable threads (c).

Twenty‐one studies reported TRAEs associated with HA fillers procedures in the lip, nasolabial folds, and lower face region. The overall prevalence of TRAEs was 63.0% (95% CI: 0.35–0.84; Figure 3a). Heterogeneity was high (*I* ^2^ = 95.7%), supporting the use of a random‐effects approach.

#### 3.5.2 Nonsurgical Facelift With Absorbable Threads

Eight studies assessed adverse outcomes following nonsurgical facelifts with absorbable threads. The overall prevalence of TRAEs was 20.0% (95% CI: 0.08%–0.41%; Figure 3b). Heterogeneity across studies was substantial (*I* ^2^ = 78%), and all analyses were performed using a random‐effects model.

#### 3.5.3 BoNT‐A

A total of 14 studies reported data on TRAEs to aesthetic use of BoNT‐A in the upper face region. The overall prevalence of patients experiencing at least one TRAE was 18% (95% CI: 0.10%–0.32%; Figure 3c). Heterogeneity among studies was high (*I* ^2^ = 96.9), justifying the use of a random‐effects model.

### 3.6 Early and Late Adverse Events

Figure S3 shows forest plots of TRAEs for HA fillers (a, b) and thread lifting (c, d). For HA fillers, pooled early TRAEs were 0.91 events/patient (95% CI: 0.47–1.77; *I* ^2^ = 99.3%) and late TRAEs 0.02 events/patient (95% CI: 0.01–0.04; *I* ^2^ = 57.6%). For thread lifting, pooled early TRAEs were 0.36 events/patient (95% CI: 0.12–1.06; *I* ^2^ = 96.8%) and late TRAEs 0.06 events/patient (95% CI: 0.03–0.15; *I* ^2^ = 75.8%). Heterogeneity was high, especially for early TRAEs. Early TRAEs were more frequent than late TRAEs in both procedures.

### 3.7 Certainty of Evidence

Very low certainty of evidence was demonstrated for the prevalence of TRAEs following HA fillers, BoNT‐A, and nonsurgical facelifts. This very low certainty was primarily due to considerable inconsistency, imprecision in all procedure types, and risk of bias, particularly in the case of nonsurgical facelifts. As such, future research is likely to have a significant impact on confidence in the effect estimates, potentially resulting in higher certainty compared to the current analysis (Table S13).

## 4 Discussion

This systematic review and meta‐analysis found an overall prevalence of TRAEs of 41.1% after aesthetic procedures involving BoNT‐A in the upper face region, HA fillers in the lips, lower face, and nasolabial folds, and nonsurgical facelift with absorbable threads. Although most TRAEs were mild and self‐limited, such as swelling, pain, and bruising, a minority of cases involved more significant or persistent events, including eyelid ptosis, nodule formation, or vascular complications. These findings highlight that although these procedures are widely considered safe, they are not without risk, reinforcing the need for appropriate technique, practitioner expertise, and patient counselling.

The growing number of aesthetic procedures parallels the global rise in life expectancy and the societal pressure to delay visible signs of aging. In this study, 87.9% of the patients evaluated were female. Literature highlights self‐confidence and improved well‐being as key motivators, particularly among middle‐aged women with higher incomes (Rohrich et al. 2009).

Commonly reported adverse events such as ecchymosis, swelling, pain, and erythema (Nikolis et al. 2021) are common during or after procedures but are not necessarily causally related to the intervention itself (Matarasso et al. 2006). These typically resolve spontaneously or with symptomatic management (Nikolis et al. 2021), justifying why most articles classified them as mild or moderate. In contrast, complications of the treatment are events with at least a reasonable causal relationship to the procedure and may result in worsened pre‐existing conditions (Mobayed et al. 2020). Examples include vascular obstruction from excessive filler (Hong et al. 2024a), diplopia after BoNT‐A (Moy et al. 2009), and infection (Bertossi et al. 2019). These iatrogenic TRAEs require professional management strategies, different from those used for common adverse effects (Hong et al. 2024a, 2024b).

HA fillers are widely used in minimally invasive aesthetic procedures (Urdiales‐Gálvez et al. 2018). Although HA is generally considered biocompatible, mild inflammatory responses have been observed histopathologically. In a retrospective multicentre study, Pires et al. (2025) documented 151 cases of late‐onset oral and perioral complications, with residual HA identified in 13 cases (11.9%). These reactions to filler material were clinically characterized as nodule/swelling, plaques, or scarring (Pires et al. 2025).

The use of hyaluronidase was reported in three studies to manage filler‐related TRAE such as lumps, nodules, or localized masses (Yazdanparast et al. 2017; Marcus et al. 2022; Alimohammadi et al. 2024). However, no consensus exists regarding the optimal dose or treatment protocol for its use in the facial region (Borzabadi‐Farahani et al. 2024). Another point worth highlighting is that HA fillers from different brands are developed using distinct technologies with varying degrees of modification (Wongprasert et al. 2022). Consequently, their safety profiles, as reported by manufacturers, may differ.

Absorbable threads have been used in suture materials since 1981 (Bertossi et al. 2019). These threads are generally reabsorbed within 8 months and incite a minimal foreign body reaction (Suh et al. 2015). Most of the TRAEs were resolved spontaneously, as reported by Singh et al. (2023). However, some cases of infection and granuloma formation required the use of antibiotics and systemic corticosteroids, respectively (Singh et al. 2023; Unal et al. 2021). In cases of dimple formation or contour irregularity, manual massages (Bertossi et al. 2019), subcision, and follow‐up were performed (Kang et al. 2017), while thread removal was necessary in cases of migration or extrusion (Bertossi et al. 2019; Singh et al. 2023). Persistent complications like these are often associated with poor technique, patient selection, or insufficient anatomical knowledge (Yi and Park 2025).

BoNT‐A is considered safe, well‐tolerated, and associated with mild, transient TRAEs (Chadha et al. 2024). Carruthers et al. (2003) demonstrated that treatment with BoNT‐A can be performed without major safety concerns when correctly administered by a professional. Comparable results were found in this study, where TRAEs were recorded in only 29.3% of cases. The main TRAEs associated with BoNT‐A were headache (30.8%), pain (13.4%), rigidity (13.6%), eye disorders (10.9%), and bruising/ecchymosis (7.9%), but only three studies classified TRAEs according to their intensity (Ahn et al. 2000; Chadha et al. 2024; Fagien et al. 2024), making it difficult to reliably analyze the intensity of symptoms.

Increasing demand for aesthetic treatments has led to a growing number of professionals entering the field (Triana et al. 2024). The literature indicates that most complications with botulinum toxin and fillers are linked to insufficient anatomical knowledge or inadequate technique (Cassuto and Sundaram 2013; Goodman et al. 2020), and serious adverse events have been reported when procedures were performed by unlicensed individuals (Centers for Disease Control and Prevention 2023). A recent Dutch cohort study also showed that injector experience was associated with vascular adverse events, although academic degree was not a predictive factor (Steenen et al. 2023).

Another important aspect is the role of professional qualifications and regulations in preventing adverse events. In the United States of America, regulatory authority over who can perform these procedures varies by state. In Alabama, physicians are authorized to perform cosmetic injectables, while dentists may administer botulinum toxin or fillers only under specific conditions regulated by the Alabama Board of Dental Examiners (Alabama Administrative Code 2024). As more professionals from diverse disciplines become involved in aesthetic medicine, evaluating their competence will become increasingly important. In the European Union, there is considerable variability regarding who is legally authorized to perform minimally invasive cosmetic procedures: while in some countries these are restricted to specialist physicians, such as dermatologists or plastic surgeons, in others they can also be performed by dentists or nurses (UK National Health Service 2023; European Commission 2022). Moreover, dermal and mucosal fillers, such as hyaluronic acid, may only be administered by appropriately trained healthcare professionals accredited under national law (European Commission 2022).

As the main limitation, there was heterogeneity regarding the overall sample among all studies included. Although some studies included all TRAEs, others only included unexpected or more severe events, which may lead to an underestimation of overall TRAEs rated. All aesthetic procedures involve some level of impact on adjacent tissues. However, the terminology related to damage notification is not standardized. This can confuse or result in errors when synthesizing studies. Standardized reporting of TRAEs associated with NSAP is essential to improve transparency, reproducibility, and safety. Therefore, we propose a structured classification system for TRAE reporting, designed to enhance consistency and transparency across studies (Table S14).

A further limitation of our review is the wide time span of the included studies, which covers more than two decades. During this period, significant improvements have occurred in filler formulations, botulinum toxin preparations, and thread‐lifting techniques, as well as in injection practices and safety protocols. These developments may have influenced both the actual occurrence and the reporting of adverse events. However, given the heterogeneity in study design and outcome reporting, a temporal subgroup analysis was not feasible. Future studies with standardized AE reporting are warranted to better capture potential temporal trends. Another relevant limitation of our review is the lack of information on post‐procedural management protocols across the included studies. Supportive measures such as the use of analgesics, anti‐inflammatory medications, cold application, antibiotics, or specific aftercare instructions may influence the onset, intensity, and duration of adverse events such as pain, swelling, or erythema. However, most studies did not provide sufficient detail regarding these aspects, which prevented us from performing any stratified analysis or adjustment. This variability in supportive care may represent a potential confounding factor and should be considered when interpreting the prevalence estimates reported in our synthesis.

## 5 Conclusions

HA fillers demonstrated the highest prevalence of TRAEs, predominantly early‐onset and self‐limiting, such as edema and pain. BoNT‐A showed the lowest rate of TRAEs, with headache being the most frequently reported complaint. Nonsurgical facelift with absorbable threads was associated with TRAEs such as pain and late‐onset events, including thread migration, which occasionally required specific interventions. Despite heterogeneity among studies, most patients experienced mild to moderate AEs. Although most events were transient and of low severity, inconsistencies in TRAE reporting and limited follow‐up across studies restrict the ability to perform direct comparisons. To optimize safety, the need for terminological standardization in future studies, technical qualifications of professionals, and prospective protocols that monitor long‐term effects is reinforced.

## Author Contributions

**Vitória Maria Sousa Cruz:** investigation, methodology, validation, visualization, writing – original draft. **Sebastião Silvério Sousa‐Neto:** investigation, writing – original draft, methodology, validation, visualization, writing – review and editing, data curation. **Caique Mariano Pedroso:** methodology, validation, visualization, writing – review and editing, formal analysis, data curation. **Maria Eduarda Pérez‐de‐Oliveira:** methodology, validation, visualization, writing – review and editing. **Ana Gabriela Costa Normando:** methodology, validation, visualization, writing – review and editing. **Helen Kaline Farias Bezerra:** methodology, validation, visualization, writing – review and editing. **Rafael Tomaz Gomes:** methodology, validation, visualization, writing – review and editing. **Ana Carolina Prado‐Ribeiro:** methodology, validation, visualization, writing – review and editing. **Danyel Elias da Cruz Perez:** writing – review and editing. **Pablo Agustin Vargas:** writing – review and editing. **Alan Roger Santos‐Silva:** conceptualization, methodology, validation, visualization, writing – review and editing, supervision.

## Ethics Statement

The authors have nothing to report.

## Consent

The authors have nothing to report.

## Conflicts of Interest

The authors declare no conflicts of interest.

## Supporting information


**Figure S1:** Flow diagram of literature search and selection criteria adapted from PRISMA (Page et al., 2020).
**Figure S2:** Risk of bias summaries of randomized controlled trials (a), non‐randomized controlled trials (c), and cohort studies (e); and graphs of randomized controlled trials (b), non‐randomized controlled trials (d), and cohort studies (f), assessed by the Joanna Briggs Institute Critical Appraisal Tools for use in JBI Systematic Reviews. Risk of bias was categorized as High when the study reaches up to 49% score “yes”, Moderate when the study reached 50% to 69% score “yes”, and Low when the study reached more than 70% score “yes” (e).
**Figure S3:** Comparison of the prevalence of early and late TRAEs after HA fillers in lips, lower face and nasolabial folds (a,b), and nonsurgical facelift with absorbable threads (c,d).

**Table S1:** PRISMA 2020 checklist.

**Table S2:** Search strategies and their results for each database and gray literature.

**Table S3:** Excluded articles and their reasons for exclusion (*n* = 348).

**Table S4:** Treatment‐related adverse events associated with aesthetic hyaluronic acid fillers in the lips, nasolabial folds, marionette lines and chin.
**Table S5:** Treatment‐related adverse events associated with aesthetic use of botulinum toxin type A in the upper face region.
**Table S6:** Treatment‐related adverse events associated with aesthetic nonsurgical facial lifting using absorbable threads.

**Table S7:** Risk of bias reviewers' summary judgments about each checklist item presented as percentages according to the randomized controlled trial studies.
**Table S8:** Risk of bias reviewers' summary judgments about each checklist item presented as percentages according to the non‐randomized controlled study.
**Table S9:** Risk of bias reviewers' summary judgments about each checklist item presented as percentages according to the cohort study.

**Table S10:** General characteristics of aesthetic procedures using HA fillers in the lips, lower face and nasolabial folds.
**Table S11:** General characteristics of aesthetic BoNT‐A injections in the upper face region (procerus, forehead, and lateral canthal region).
**Table S12:** General characteristics of aesthetic nonsurgical facelift procedures using absorbable threads.

**Table S13:** Grading of Recommendation, Assessment, Development, and Evaluation (GRADE) evidence profile.

**Table S14:** Checklist for Treatment‐Related Adverse Event (TRAE) Reporting in Minimally Invasive Aesthetic Treatments.

**Appendix S1:** List of references of articles included in this systematic review.

## Data Availability

The data that supports the findings of this study are available in the Supporting Information of this article.
