# Supplementary material for: Adverse Events in Nonsurgical Facial Aesthetic Procedures: A Systematic Review and Meta‐Analysis
Source: Oral Dis. 2025 Oct 5;32(2):384–94. doi: 10.1111/odi.70109 (PMC13077022; doi:10.1111/odi.70109)
Supplement: Supplementary file 1 — Figure S1: Flow diagram of literature search and selection criteria adapted from PRISMA (Page et al., 2020). Figure S2: Risk of bias summaries of randomized controlled trials (a), non‐randomized controlled trials (c), and cohort studies (e); and graphs of randomized controlled trials (b), non‐randomized controlled trials (d), and cohort studies (f), assessed by the Joanna Briggs Institute Critical Appraisal Tools for use in JBI Systematic Reviews. Risk of bias was categorized as High when the study reaches up to 49% score “yes”, Moderate when the study reached 50% to 69% score “yes”, and Low when the study reached more than 70% score “yes” (e). Figure S3: Comparison of the prevalence of early and late TRAEs after HA fillers in lips, lower face and nasolabial folds (a,b), and nonsurgical facelift with absorbable threads (c,d). [file ODI-32-384-s008.docx]

**SUPPLEMENTARY FIGURES**

**Supplementary Figure 1**


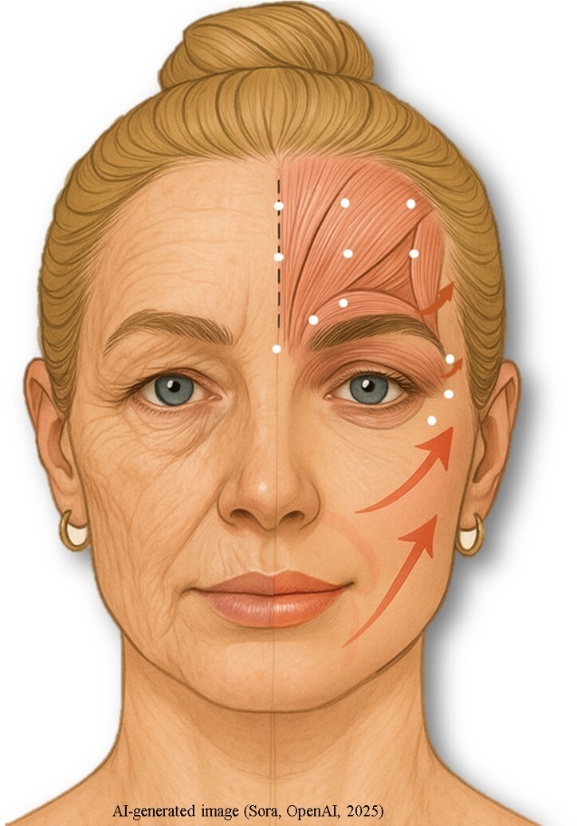


**Supplementary Figure 2**


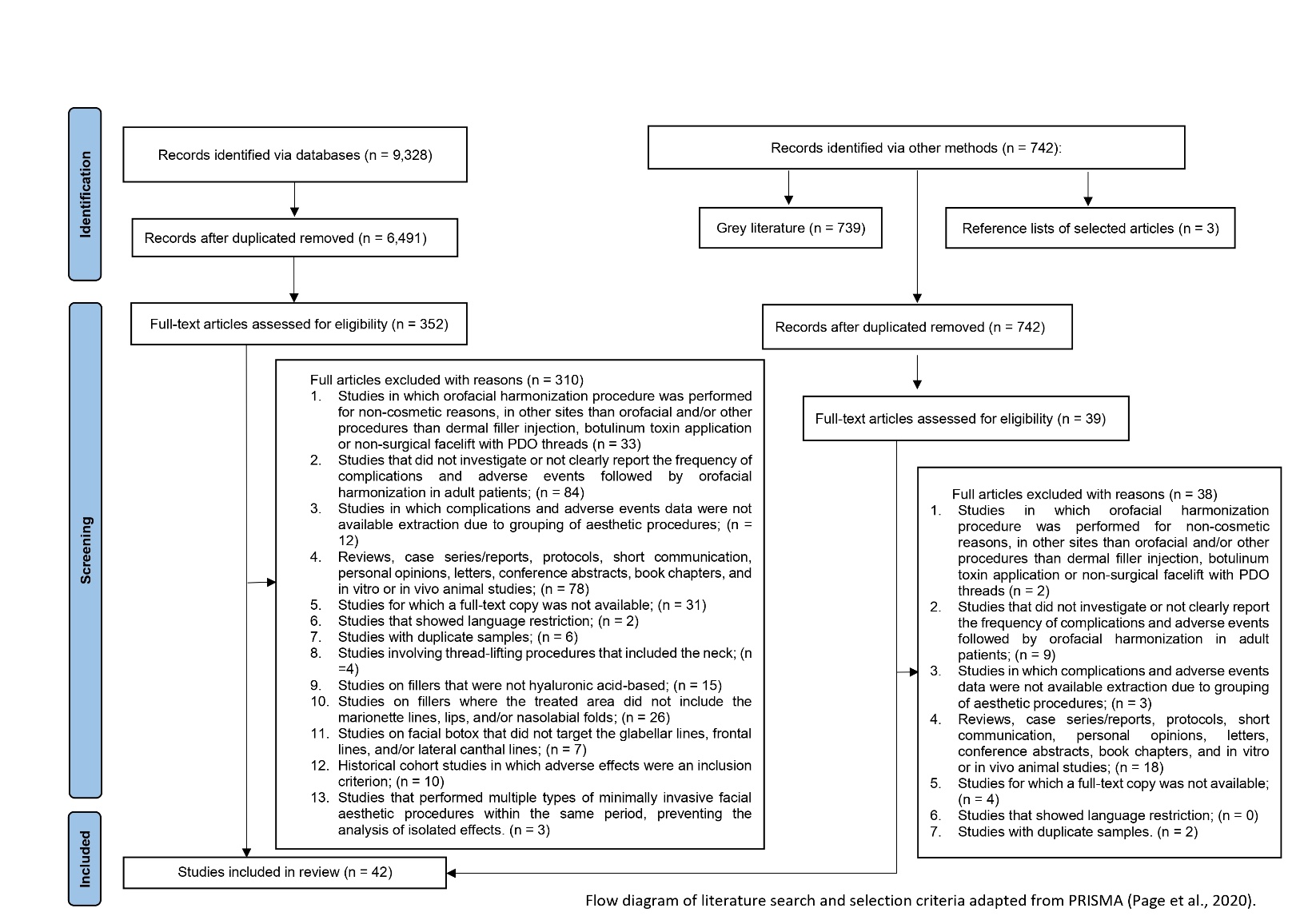


**Supplementary Figure 3**


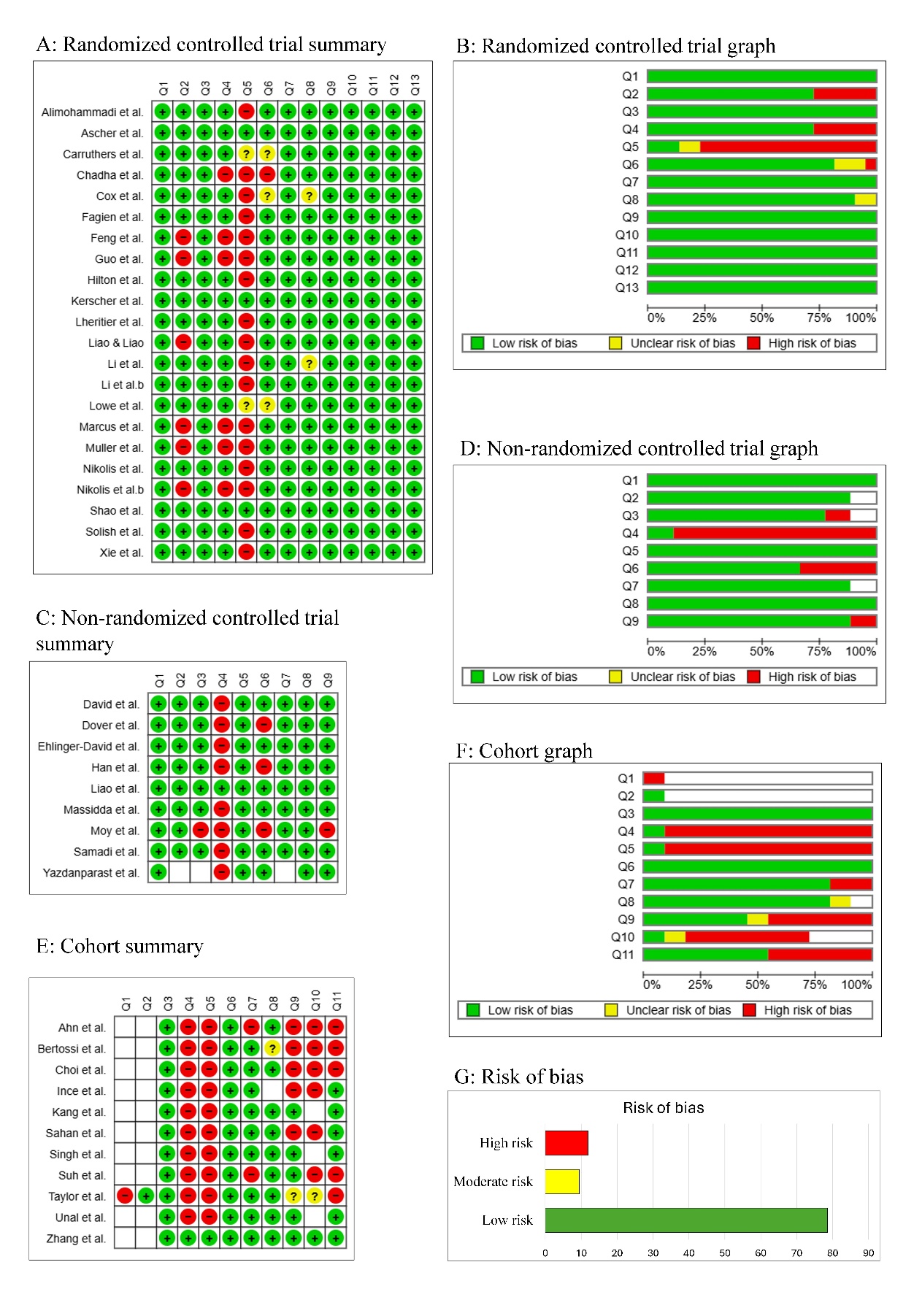


Supplementary Figure 4


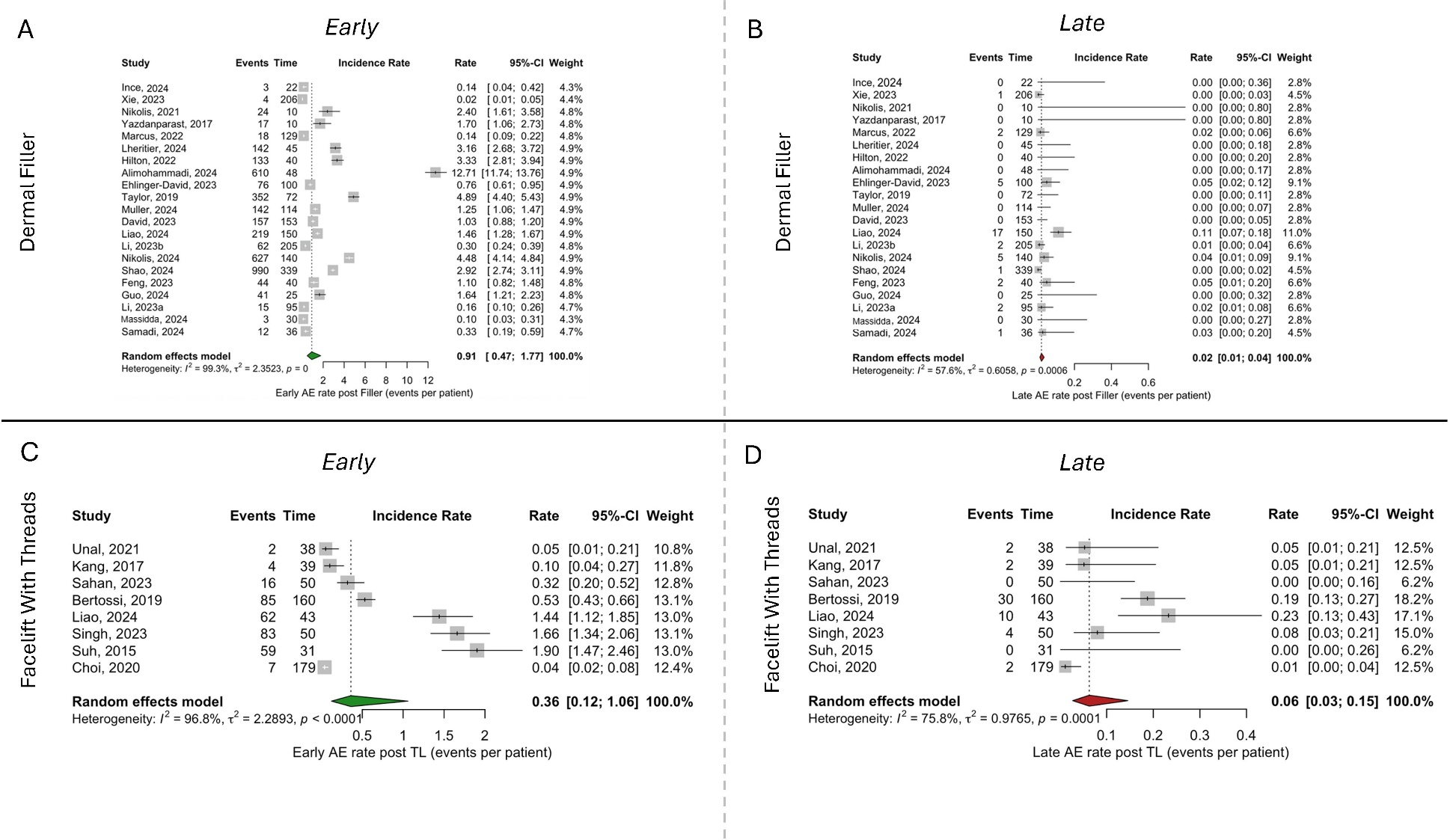


**SUPPLEMENTARY FIGURE LEGENDS**

**SUPPLEMENTARY FIGURE S1** Illustration representing the regions per procedure considered for the studies included in this review. **Orange arrows** – Indicate facelifts performed with absorbable threads in various regions of the face, excluding the neck. **White dots** – Indicate the most common points of botulinum toxin application in three upper facial regions included in the study: the external canthal area (crow’s feet), the glabellar/procerus complex, and the forehead/frontal region. **Pink shading** – Highlights the labial region, chin, nasolabial folds and marionette lines. Studies were included in which hyaluronic acid-based dermal fillers were applied.

**SUPPLEMENTARY FIGURE S2** Flow diagram of literature search and selection criteria adapted from PRISMA (Page et al., 2020).

**SUPPLEMENTARY FIGURE S3** Risk of bias summaries of randomized controlled trials (a), non-randomized controlled trials (c), and cohort studies (e); and graphs of randomized controlled trials (b), non-randomized controlled trials (d), and cohort studies (f), assessed by the Joanna Briggs Institute Critical Appraisal Tools for use in JBI Systematic Reviews. Risk of bias was categorized as High when the study reaches up to 49% score “yes”, Moderate when the study reached 50% to 69% score “yes”, and Low when the study reached more than 70% score “yes” (e).

**SUPPLEMENTARY FIGURE S4** Comparison of the prevalence of early and late TRAEs after HA fillers in lips, lower face and nasolabial folds (a,b), and non-surgical facelift with absorbable threads (c,d).
