# Supplementary material for: Adverse Events in Nonsurgical Facial Aesthetic Procedures: A Systematic Review and Meta‐Analysis
Source: Oral Dis. 2025 Oct 5;32(2):384–94. doi: 10.1111/odi.70109 (PMC13077022; doi:10.1111/odi.70109)
Supplement: Supplementary file 3 — Table S2: Search strategies and their results for each database and gray literature. [file ODI-32-384-s006.docx]

**Supplementary Table S2** - Search strategies and their results for each database and grey literature.

| **Databases** | **Search strategies** *(Search date: December 12th, 2024)* | **Results** |
| --- | --- | --- |
| **PUBMED** | (“dermal fillers”[MeSH Terms] OR “dermal fillers” OR filler OR fillers OR filling lift OR lifting OR lipectomy[MeSH Terms] OR lipectomy OR lipectomies OR lipolysis OR liposuction OR liposuctions OR lipoplasty OR lipoplasties OR bichectomy OR “botulinum toxins”[MeSH Terms] OR “botulinum toxins” OR “botulinum toxin” OR “botulinum neurotoxins” OR “clostridium botulinum” OR botulin OR botox) **AND** (“adverse event” OR “adverse events” OR “adverse reactions” OR “adverse reaction” OR complication OR complications) **AND** (face[MeSH Terms] OR face OR facial OR mouth[MeSH Terms] OR mouth OR oral OR perioral OR orofacial) | **4,354** |
| **SCOPUS** | TITLE-ABS-KEY(filler OR fillers OR filling lift OR lifting OR lipectomy OR lipectomies OR lipolysis OR liposuction OR liposuctions OR lipoplasty OR lipoplasties OR bichectomy OR “botulinum toxins” OR “botulinum toxin” OR “botulinum neurotoxins” OR “clostridium botulinum” OR botulin OR botox) **AND** TITLE-ABS-KEY (“adverse event” OR “adverse events” OR “adverse reactions” OR “adverse reaction” OR complication OR complications) **AND** TITLE-ABS-KEY (face OR facial OR mouth OR oral OR perioral OR orofacial) | **329** |
| **EMBASE** | (('filler'/de OR fillers OR filling) AND 'lift'/de OR 'lifting'/de OR 'lipectomy'/de OR lipectomies OR 'lipolysis'/de OR 'liposuction'/de OR liposuctions OR 'lipoplasty'/de OR lipoplasties OR bichectomy OR 'botulinum toxins'/de OR 'botulinum toxin'/de OR 'botulinum neurotoxins' OR 'clostridium botulinum'/de OR 'botulin'/de OR 'botox'/de) **AND** ('adverse event'/de OR 'adverse events'/de OR 'adverse reactions' OR 'adverse reaction'/de OR 'complication'/de OR 'complications'/de) **AND** ('face'/de OR facial OR 'mouth'/de OR oral OR perioral OR orofacial) | **511** |
| **WEB OF SCIENCE** | TS=(filler OR fillers OR filling lift OR lifting OR lipectomy OR lipectomies OR lipolysis OR liposuction OR liposuctions OR lipoplasty OR lipoplasties OR bichectomy OR “botulinum toxins” OR “botulinum toxin” OR “botulinum neurotoxins” OR “clostridium botulinum” OR botulin OR botox) **AND** TS=(“adverse event” OR “adverse events” OR “adverse reactions” OR “adverse reaction” OR complication OR complications) **AND** TS=(face OR facial OR mouth OR oral OR perioral OR orofacial) | **3,350** |
| **LILACS** | (“dermal fillers” OR “preenchedores dérmicos” OR “rellenos dérmicos” OR “botulinum toxins” OR “toxinas botulínicas” OR lipectomy OR lipectomia OR lipectomía) **AND** (face OR “mouth mucosa” OR “mucosa bucal”) | **45** |
| **GOOGLE SCHOLAR** | (filler OR “botulinum toxin” OR lipoplasty OR bichectomy) AND (complications OR "adverse events") AND (face OR facial) | **100** |
| **ProQUEST** | TI,AB(filler OR fillers OR filling lift OR lifting OR lipectomy OR lipectomies OR lipolysis OR liposuction OR liposuctions OR lipoplasty OR lipoplasties OR bichectomy OR “botulinum toxins” OR “botulinum toxin” OR “botulinum neurotoxins” OR “clostridium botulinum” OR botulin OR botox) **AND** TI,AB(“adverse event” OR “adverse events” OR “adverse reactions” OR “adverse reaction” OR complication OR complications) **AND** TI,AB(face OR facial OR mouth OR oral OR perioral OR orofacial) | **639** |
