# Supplementary material for: Adverse Events in Nonsurgical Facial Aesthetic Procedures: A Systematic Review and Meta‐Analysis
Source: Oral Dis. 2025 Oct 5;32(2):384–94. doi: 10.1111/odi.70109 (PMC13077022; doi:10.1111/odi.70109)
Supplement: Supplementary file 4 — Table S3: Excluded articles and their reasons for exclusion (n = 348). [file ODI-32-384-s003.docx]

**Supplementary Table S3.** Excluded articles and reasons for exclusion (n= 348)

| References | | Reasons for exclusion | |
| --- | --- | --- | --- |
| 1. ANTON, Christopher. Botulinum toxins: adverse effects. Adverse Drug Reaction Bulletin, n. 267, p. 1027-1030, 2011. | 4 | |  |
| 1. DAYAN, Steven H. Complications from toxins and fillers in the dermatology clinic: recognition, prevention, and treatment. Facial Plastic Surgery Clinics, v. 21, n. 4, p. 663-673, 2013. | 4 | |  |
| 1. HUANG, Lily et al. Safety of Botulinum Neurotoxin Injection to the Central Upper Eyelid and Eyebrow Regions. Investigative Ophthalmology & Visual Science, v. 54, n. 15, p. 741-741, 2013. | 5 | |  |
| 1. FISCHER, Tanja et al. Safety, Tolerability, and Efficacy of Repeat-Dose Injections of IncobotulinumtoxinA in the Treatment of Upper Facial Lines: Results from a Prospective, Open-Label, Phase III Study. Journal of Drugs in Dermatology: JDD, v. 19, n. 5, p. 461-469, 2020. | 5 | |  |
| 1. MILANI, C.; MILANI, S. L. S. Severe adverse reactions after botulinum toxin treatment. In: MOVEMENT DISORDERS. 111 RIVER ST, HOBOKEN 07030-5774, NJ USA: WILEY-BLACKWELL, 2013. p. S389-S390. | 5 | |  |
| 1. PARK, Je-Young et al. IncobotulinumtoxinA injection for sebum control, face lifting, and pore size improvement. Toxicon, v. 190, p. S56-S57, 2021. | 4 | |  |
| 1. RAZUMOVSKAIA, Elena. CLINICAL ASPECTS OF USING AN UPPER FACE MAPPING SYSTEM FOR ADMINISTRATION OF BOTOX®. Toxicon, v. 156, p. S96, 2018. | 4 | |  |
| 1. TREVIDIC, Patrick et al. Safety and efficacy of incobotulinumtoxinA for the treatment of upper facial lines: A randomized, double-blind, placebo-controlled, phase 3 study with open-label extension. Toxicon, v. 156, p. S110, 2018. | 4 | |  |
| 1. ALAM, Murad et al. Multicenter prospective cohort study of the incidence of adverse events associated with cosmetic dermatologic procedures: lasers, energy devices, and injectable neurotoxins and fillers. JAMA dermatology, v. 151, n. 3, p. 271-277, 2015. | 1 | |  |
| 1. ANAND, Chytra. Facial Contouring With Fillers, Neuromodulators, and Lipolysis to Achieve a Natural Look in Patients With Facial Fullness. Journal of Drugs in Dermatology: JDD, v. 15, n. 12, p. 1536-1542, 2016. | 5 | |  |
| 1. ASCHER, B.; ROSSI, B. Botulinum toxin and wrinkles: few side effects and effective combining procedures with other treatments. In: Annales de Chirurgie Plastique et Esthetique. 2004. p. 537-552. | 4 | |  |
| 1. CARRUTHERS, Alastair et al. Multicenter, randomized, parallel‐group study of the safety and effectiveness of onabotulinumtoxinA and hyaluronic acid dermal fillers (24‐mg/ml smooth, cohesive gel) alone and in combination for lower facial rejuvenation. Dermatologic surgery, v. 36, p. 2121-2134, 2010. | 3 | |  |
| 1. COX, Sue Ellen; ADIGUN, Chris G. Complicações de preenchedores injetáveis ​​e neurotoxinas. Terapia Dermatológica , v. 24, n. 6, pág. 524-536, 2011. | 4 | |  |
| 1. DA CUNHA, Marisa Gonzaga et al. Superficial application technique with cohesive polydensified matrix hyaluronic acid for the treatment of lines and wrinkles. Surgical & cosmetic dermatology, v. 11, n. 3, p. 205-210, 2019. | 2 | |  |
| 1. DELORENZI, Claudio. Complications of injectable fillers, part 2: vascular complications. Aesthetic surgery journal, v. 34, n. 4, p. 584-600, 2014. | 4 | |  |
| 1. DU, YX; WU, L.; SHEN, Y. Avanços nas complicações oculares iatrogênicas causadas por injeções cosméticas faciais. [Zhonghua yan ke za Zhi] Revista Chinesa de Oftalmologia , v. 57, n. 5, pág. 391-395, 2021. | 6 | |  |
| 1. GOLDMAN, Alberto; WOLLINA, Uwe. Polymethylmethacrylate‐induced nodules of the lips: Clinical presentation and management by intralesional neodymium: YAG laser therapy. Dermatologic Therapy, v. 32, n. 1, p. e12755, 2019. | 4 | |  |
| 1. HEDÉN, Per et al. Effective and safe repeated full-face treatments with abobotulinumtoxina, hyaluronic acid filler, and skin boosting hyaluronic acid. Journal of Drugs in Dermatology: JDD, v. 18, n. 7, p. 682-689, 2019. | 5 | |  |
| 1. HU, Yifan et al. Efficacy and safety of two hyaluronic acid fillers with different injection depths for the correction of moderate‐to‐severe nasolabial folds: A 52‐week, prospective, randomized, double‐blinded study in a Chinese population. Journal of Cosmetic Dermatology, v. 21, n. 3, p. 940-948, 2022. | 5 | |  |
| 1. HUMPHREY, Shannon; CARRUTHERS, Jean; CARRUTHERS, Alastair. Clinical experience with 11,460 mL of a 20-mg/mL, smooth, highly cohesive, viscous hyaluronic acid filler. Dermatologic Surgery, v. 41, n. 9, p. 1060-1067, 2015. | 2 | |  |
| 1. KANG, Seung H.; MOON, Seok H.; KIM, Hei S. Nonsurgical rhinoplasty with polydioxanone threads and fillers. Dermatologic surgery, v. 46, n. 5, p. 664-670, 2020. | 3 | |  |
| 1. LUPO, Mary P. et al. Effectiveness of Juvéderm Ultra Plus dermal filler in the treatment of severe nasolabial folds. Plastic and reconstructive surgery, v. 121, n. 1, p. 289-297, 2008. | 2 | |  |
| 1. NGUYEN, Tam T. Plastic Surgery and Cosmetic Procedures: Facial Injection Procedures. FP essentials, v. 497, p. 11-17, 2020. | 5 | |  |
| 1. PAVICIC, Tatiana. Calcium hydroxylapatite filler: an overview of safety and tolerability. Journal of Drugs in Dermatology: JDD, v. 12, n. 9, p. 996-1002, 2013. | 5 | |  |
| 1. PRAGER, Welf et al. Mid-Face Volumization With Hyaluronic Acid: Injection Technique and Safety Aspects from a Controlled, Randomized, Double-Blind Clinical Study. Journal of Drugs in Dermatology: JDD, v. 16, n. 4, p. 351-357, 2017. | 5 | |  |
| 1. RYU, Hwa Jung et al. New classification of late and delayed complications after dermal filler: Localized or Generalized?. Journal of Cosmetic and Laser Therapy, v. 22, n. 6-8, p. 244-252, 2020. | 4 | |  |
| 1. SINGH, Kuldeep; NOOREYEZDAN, Shahin. Nonvascular Complications of Injectable Fillers—Prevention and Management. Indian Journal of Plastic Surgery, v. 53, n. 03, p. 335-343, 2020. | 4 | |  |
| 1. SMALL, Rebecca. Botulinum toxin injection for facial wrinkles. American family physician, v. 90, n. 3, p. 168-175, 2014. | 4 | |  |
| 1. CHEN, Shuyue; LONG, Jianhong. Adverse events of botulinum toxin A in facial injection: Mechanism, prevention and treatment. Zhong nan da xue xue bao. Yi xue ban= Journal of Central South University. Medical sciences, v. 44, n. 7, p. 837-844, 2019. | 6 | |  |
| 1. COHEN, Sarit; ARTZI, Ofir; HELLER, Lior. Forehead lift using botulinum toxin. Aesthetic Surgery Journal, v. 38, n. 3, p. 312-320, 2018. | 2 | |  |
| 1. BERTUCCI, Vince et al. Efficacy and Safety of Flexible Hyaluronic Acid Fillers in Lip and Perioral Enhancement. Journal of Drugs in Dermatology: JDD, v. 20, n. 4, p. 402-408, 2021. | 5 | |  |
| 1. MANDAL, Priyanka; GAMA, Frank. The use of periocular fillers in aesthetic medicine. Journal of Plastic, Reconstructive & Aesthetic Surgery, v. 74, n. 7, p. 1602-1609, 2021. | 4 | |  |
| 1. MONHEIT, Gary D. et al. Efficacy, safety, and subject satisfaction after abobotulinumtoxinA treatment for moderate to severe glabellar lines. Dermatologic Surgery, v. 46, n. 1, p. 61-69, 2020. | 2 | |  |
| 1. VARTANIAN, A. John; DAYAN, Steven H. Complications of botulinum toxin A use in facial rejuvenation. Facial Plastic Surgery Clinics, v. 11, n. 4, p. 483-492, 2003. | 4 | |  |
| 1. FRAMPTON, James E.; EASTHOPE, Stephanie E. Botulinum Toxin A (Botox® Cosmetic). American journal of clinical dermatology, v. 4, n. 10, p. 709-725, 2003. | 2 | |  |
| 1. NIAMTU III, Joseph. Complications in fillers and Botox. Oral and maxillofacial surgery clinics of North America, v. 21, n. 1, p. 13-21, 2009. | 4 | |  |
| 1. BERTOSSI, Dario et al. The facial grid analysis for filler injection: a cohort study of 300 patients. Giornale Italiano di Dermatologia e Venereologia: Organo Ufficiale, Societa Italiana di Dermatologia e Sifilografia, v. 151, n. 5, p. 467-472, 2015. | 5 | |  |
| 1. KARBASSI, Esmat; NAKHAEE, Nouzar; ZAMANIAN, Maryam. The efficacy and complications of a new technique of Abobotulinum‐toxin A (Dysport) injection in patients with glabellar lines. Journal of cosmetic dermatology, v. 18, n. 1, p. 55-58, 2019. | 2 | |  |
| 1. CARRUTHERS, Jean; CARRUTHERS, Alastair. Botulinum toxin type A treatment of multiple upper facial sites: patient‐reported outcomes. Dermatologic surgery, v. 33, p. S10-S17, 2007. | 2 | |  |
| 1. WEISS, Robert et al. A randomized, controlled, evaluator-blinded, multi-center study of hyaluronic acid filler effectiveness and safety in lip fullness augmentation. Dermatologic Surgery, v. 47, n. 4, p. 527, 2021. | 2 | |  |
| 1. FENG, Zheng et al. Optimal dosage of botulinum toxin type A for treatment of glabellar frown lines: efficacy and safety in a clinical trial. Dermatologic Surgery, v. 41, p. S56-S63, 2015. | 4 | |  |
| 1. RZANY, Berthold et al. Repeated botulinum toxin A injections for the treatment of lines in the upper face: a retrospective study of 4,103 treatments in 945 patients. Dermatologic surgery, v. 33, p. S18-S25, 2007. | 3 | |  |
| 1. RZANY, Berthold-Josef et al. A multicenter, randomized, double-blind, placebo-controlled, single-dose, phase III, non-inferiority study comparing PrabotulinumtoxinA and OnabotulinumtoxinA for the treatment of moderate to severe glabellar lines in adult patients. Aesthetic surgery journal, v. 40, n. 4, p. 413-429, 2020. | 7 | |  |
| 1. CARRUTHERS, Alastair; CARRUTHERS, Jean; SAID, Samireh. Dose‐ranging study of botulinum toxin type A in the treatment of glabellar rhytids in females. Dermatologic surgery, v. 31, n. 4, p. 414-422, 2005. | 7 | |  |
| 1. ZACCARIA, Giovanna et al. Filler-induced complications of the lips: 10 years experience with intralesional laser treatment and refinements. Journal of Plastic, Reconstructive & Aesthetic Surgery, v. 75, n. 3, p. 1215-1223, 2022. | 2 | |  |
| 1. STREKER, Meike et al. Patient-reported outcomes after incobotulinumtoxinA treatment for upper facial wrinkles. Dermatologic Surgery, v. 41, p. S29-S38, 2015. | 2 | |  |
| 1. MCCRACKEN, Michael S. et al. Hyaluronic acid gel (Restylane) filler for facial rhytids: lessons learned from American Society of Ophthalmic Plastic and Reconstructive Surgery member treatment of 286 patients. Ophthalmic Plastic & Reconstructive Surgery, v. 22, n. 3, p. 188-191, 2006. | 2 | |  |
| 1. DE ALMEIDA, Ada Trindade et al. Patient satisfaction and safety with aesthetic onabotulinumtoxinA after at least 5 years: a retrospective cross-sectional analysis of 4,402 glabellar treatments. Dermatologic Surgery, v. 41, p. S19-S28, 2015. | 3 | |  |
| 1. SCHLESSINGER, Joel et al. Long‐term safety of abobotulinumtoxinA for the treatment of glabellar lines: results from a 36‐month, multicenter, open‐label extension study. Dermatologic Surgery, v. 40, n. 2, p. 176-183, 2014. | 2 | |  |
| 1. SUN, Yi et al. Laser lipolysis used to treat localized adiposis: a preliminary report on experience with Asian patients. Aesthetic plastic surgery, v. 33, n. 5, p. 701-705, 2009. | 1 | |  |
| 1. CARRUTHERS, J. Alastair et al. A multicenter, double-blind, randomized, placebo-controlled study of the efficacy and safety of botulinum toxin type A in the treatment of glabellar lines. Journal of the american academy of dermatology, v. 46, n. 6, p. 840-849, 2002. | 3 | |  |
| 1. CARRUTHERS, Jean et al. A multicenter, randomized, double-blind, placebo-controlled study to evaluate the efficacy and safety of repeated onabotulinumtoxinA treatments in subjects with crow's feet lines and glabellar lines. Dermatologic Surgery, v. 41, n. 6, p. 702-711, 2015. | 2 | |  |
| 1. O'RYAN, Felice; SCHENDEL, Stephen; POOR, David. Submental-submandibular suction lipectomy: indications and surgical technique. Oral surgery, oral medicine, oral pathology, v. 67, n. 2, p. 117-125, 1989. | 4 | |  |
| 1. LAWSON, William; NAIDU, Rahul K. The male facelift: an analysis of 115 cases. Archives of Otolaryngology–Head & Neck Surgery, v. 119, n. 5, p. 535-539, 1993. | 4 | |  |
| 1. SEO, Kyle et al. Efficacy and Safety of IncobotulinumtoxinA in Asian Subjects: A Pooled Analysis of Clinical Trials in the Treatment of Glabellar Frown Lines. Journal of Drugs in Dermatology: JDD, v. 15, n. 9, p. 1084-1087, 2016. | 5 | |  |
| 1. KLEIN, Arnold William. Complications and adverse reactions with the use of botulinum toxin. Disease-A-Month, v. 48, n. 5, p. 336-356, 2002. | 4 | |  |
| 1. KIM, Jung Eun et al. The efficacy and safety of liquid-type botulinum toxin type A for the management of moderate to severe glabellar frown lines. Plastic and Reconstructive Surgery, v. 135, n. 3, p. 732-741, 2015. | 2 | |  |
| 1. JOSEPH, John et al. AbobotulinumtoxinA for the Treatment of Moderate-to-Severe Glabellar Lines: A Randomized, Dose-Escalating, Double-Blind Study. Journal of Drugs in Dermatology: JDD, v. 20, n. 9, p. 980-987, 2021. | 2 | |  |
| 1. KANE, Michael AC et al. Evaluation of variable-dose treatment with a new US Botulinum Toxin Type A (Dysport) for correction of moderate to severe glabellar lines: results from a phase III, randomized, double-blind, placebo-controlled study. Plastic and reconstructive surgery, v. 124, n. 5, p. 1619-1629, 2009. | 2 | |  |
| 1. SULOVSKY, Monika et al. A prospective open‐label, multicentre study evaluating a non‐cross‐linked hyaluronic acid based soft‐tissue filler in the correction of lateral canthal and perioral lines. Journal of Cosmetic Dermatology, v. 21, n. 1, p. 191-198, 2022. | 2 | |  |
| 1. HEVIA, Oscar. Retrospective review of 500 patients treated with abobotulinumtoxinA. Journal of Drugs in Dermatology: JDD, v. 9, n. 9, p. 1081-1084, 2010. | 5 | |  |
| 1. SOLISH, Nowell et al. Efficacy and safety of onabotulinumtoxinA treatment of forehead lines: a multicenter, randomized, dose-ranging controlled trial. Dermatologic Surgery, v. 42, n. 3, p. 410-419, 2016. | 2 | |  |
| 1. LEE, Ko‐Eun; KIM, Gye‐Jung; SA, Ho‐Seok. The clinical spectrum of periorbital vascular complications after facial injection. Journal of Cosmetic Dermatology, v. 20, n. 5, p. 1532-1540, 2021. | 2 | |  |
| 1. DELORENZI, Claudio. New high dose pulsed hyaluronidase protocol for hyaluronic acid filler vascular adverse events. Aesthetic Surgery Journal, v. 37, n. 7, p. 814-825, 2017. | 2 | |  |
| 1. SMITH, Stacy R. et al. Functional safety assessments used in a randomized controlled study of small gel particle hyaluronic acid for lip augmentation. Dermatologic Surgery, v. 41, p. S137-S142, 2015. | 2 | |  |
| 1. NGUYEN, Tuyet A.; REDDY, Shivani; GHARAVI, Nima. Specific complications associated with non-surgical rhinoplasty. Journal of Cosmetic and Laser Therapy, v. 22, n. 4-5, p. 171-173, 2020. | 4 | |  |
| 1. FITZGERALD, Rebecca et al. Adverse reactions to injectable fillers. Facial Plastic Surgery, v. 32, n. 05, p. 532-555, 2016. | 5 | |  |
| 1. ABDELMOHSEN, Mohsen Ahmed. Injectable fillers: imaging features and related complications. Egyptian Journal of Radiology and Nuclear Medicine, v. 51, n. 1, p. 1-9, 2020. | 1 | |  |
| 1. ASCHER, Benjamin et al. Efficacy and safety of a new hyaluronic acid dermal filler in the treatment of severe nasolabial lines–6‐month interim results of a randomized, evaluator‐blinded, intra‐individual comparison study. Journal of Cosmetic Dermatology, v. 10, n. 2, p. 94-98, 2011. | 2 | |  |
| 1. LEW, Helen et al. Effect of botulinum toxin A on facial wrinkle lines in Koreans. Ophthalmologica, v. 216, n. 1, p. 50-54, 2002. | 3 | |  |
| 1. KWON, Hyun Jung et al. The efficacy and safety of a monophasic hyaluronic acid filler in the correction of nasolabial folds: A randomized, multicenter, single blinded, split‐face study. Journal of Cosmetic Dermatology, v. 17, n. 4, p. 584-589, 2018. | 2 | |  |
| 1. BERTOSSI, Dario et al. Injectable profiloplasty: forehead, nose, lips, and chin filler treatment. Journal of Cosmetic Dermatology, v. 18, n. 4, p. 976-984, 2019. | 2 | |  |
| 1. MATARASSO, Seth L. Complications of botulinum A exotoxin for hyperfunctional lines. Dermatologic surgery, v. 24, n. 11, p. 1249-1254, 1998. | 4 | |  |
| 1. DE SANTIS, Giorgio et al. Lipofilling after laser-assisted treatment for facial filler complication: Volumetric and regenerative effect. Plastic and Reconstructive Surgery, v. 147, n. 3, p. 585-591, 2021. | 1 | |  |
| 1. AHSANUDDIN, Salma et al. Adverse events associated with Botox as reported in a Food and Drug Administration Database. Aesthetic Plastic Surgery, v. 45, n. 3, p. 1201-1209, 2021. | 1 | |  |
| 1. BERMAN, Walter E.; MONETI, Charles M. Complications in blepharoplasties and face lift operations. Otolaryngologic Clinics of North America, v. 7, n. 1, p. 87-105, 1974. | 5 | |  |
| 1. BERRY, M. G.; DAVIES, Dai. Platysma-SMAS plication facelift. Journal of plastic, reconstructive & aesthetic surgery, v. 63, n. 5, p. 793-800, 2010. | 1 | |  |
| 1. BLOOM, Jason D.; IMMERMAN, Sara B.; ROSENBERG, David Brent. Face-lift complications. Facial Plastic Surgery, v. 28, n. 03, p. 260-272, 2012. | 5 | |  |
| 1. CÁRDENAS-CAMARENA, Lázaro et al. Strategies for reducing fatal complications in liposuction. Plastic and reconstructive surgery Global open, v. 5, n. 10, 2017. | 1 | |  |
| 1. LEE, Kevin C. et al. What are the most commonly reported complications with cosmetic botulinum toxin type A treatments?. Journal of Oral and Maxillofacial Surgery, v. 78, n. 7, p. 1190. e1-1190. e9, 2020. | 2 | |  |
| 1. KIM, Byung Wook et al. Adverse events associated with botulinum toxin injection: a multidepartment, retrospective study of 5310 treatments administered to 1819 patients. Journal of dermatological treatment, v. 25, n. 4, p. 331-336, 2014. | 1 | |  |
| 1. CHEN, Youbai et al. Treatment of Complications following Facial Thread-Lifting. Plastic and Reconstructive Surgery, v. 148, n. 1, p. 159e-161e, 2021. | 4 | |  |
| 1. CHOI, Hwan Jun; LEE, Jun Beom. The Complication of Middle Facelift Surgery Resulting in Migration of Temporal Augmentation Implant. Journal of Craniofacial Surgery, v. 27, n. 4, p. e413-e414, 2016. | 4 | |  |
| 1. MOERS-CARPI, Marion et al. European Multicenter Prospective Study Evaluating Long-Term Safety and Efficacy of the Polycaprolactone-Based Dermal Filler in Nasolabial Fold Correction. Dermatologic Surgery, v. 47, n. 7, p. 960-965, 2021. | 5 | |  |
| 1. WOLLINA, Uwe; KONRAD, Helga. Managing adverse events associated with botulinum toxin type A. American journal of clinical dermatology, v. 6, n. 3, p. 141-150, 2005. | 4 | |  |
| 1. BEER, Kenneth et al. Safe and effective chin augmentation with the hyaluronic acid injectable filler, VYC-20L. Dermatologic Surgery, v. 47, n. 1, p. 80, 2021. | 2 | |  |
| 1. CARRUTHERS, Alastair et al. Dose‐finding, safety, and tolerability study of botulinum toxin type B for the treatment of hyperfunctional glabellar lines. Dermatologic surgery, v. 33, p. S60-S68, 2007. | 2 | |  |
| 1. DAUWE, Phillip B. et al. Infection in face-lift surgery: an evidence-based approach to infection prevention. Plastic and reconstructive surgery, v. 135, n. 1, p. 58e-66e, 2015. | 4 | |  |
| 1. DURANTI, Fabrizio et al. Injectable hyaluronic acid gel for soft tissue augmentation: A clinical and histological study. Dermatologic surgery, v. 24, n. 12, p. 1317-1325, 1998. | 2 | |  |
| 1. ELLENBOGEN, Richard. A 15-year follow-up study of the non-SMAS skin-tightening facelift with midface defatting: Equal or better than deeper plane procedures in result, duration, safety, and patient satisfaction. Clinics in plastic surgery, v. 24, n. 2, p. 247-267, 1997. | 4 | |  |
| 1. FABBROCINI, Gabriella et al. Adverse reaction of temporary fillers used for treating facial aging. In: JOURNAL OF THE AMERICAN ACADEMY OF DERMATOLOGY. 360 PARK AVENUE SOUTH, NEW YORK, NY 10010-1710 USA: MOSBY-ELSEVIER, 2011. p. AB24-AB24. | 5 | |  |
| 1. FABI, Sabrina G. et al. A prospective multicenter pilot study of the safety and efficacy of microfocused ultrasound with visualization for improving lines and wrinkles of the décolleté. Dermatologic Surgery, v. 41, n. 3, p. 327-335, 2015. | 1 | |  |
| 1. FEDOK, Fred G. The avoidance and management of complications, and revision surgery of the lower face and neck. Clinics in Plastic Surgery, v. 45, n. 4, p. 623-634, 2018. | 1 | |  |
| 1. FERREIRA FILHO, José Laurentino et al. Oral and Maxillofacial Complications due to the use of Hyaluronic Acid as an Alternative for Facial Implants. Journal of Young Pharmacists, v. 13, n. 1, p. 14, 2021. | 4 | |  |
| 1. FLEURY, Christopher M. et al. Adverse event incidences following facial plastic surgery procedures: incorporating FACE-Q data to improve patient preparation. Plastic and Reconstructive Surgery, v. 141, n. 1, p. 28e-33e, 2018. | 1 | |  |
| 1. KANG, Seung Hoon et al. Wedge‐shaped polydioxanone threads in a folded configuration (“Solid fillers”): A treatment option for deep static wrinkles on the upper face. Journal of cosmetic dermatology, v. 18, n. 1, p. 65-70, 2019. | 2 | |  |
| 1. HU, Xiaogen et al. Comparative study of autologous fat vs hyaluronic acid in correction of the nasolabial folds. Journal of Cosmetic Dermatology, v. 16, n. 4, p. e1-e8, 2017. | 3 | |  |
| 1. GASSIA, V. Prevention and management of locoregional complications of botulinum A toxin injections in cosmetic treatment. In: Annales de Dermatologie et de Venereologie. 2009. p. S146-51. | 4 | |  |
| 1. GRAIVIER, Miles H. et al. Differentiating nonpermanent injectable fillers: prevention and treatment of filler complications. Aesthetic Surgery Journal, v. 38, n. suppl_1, p. S29-S40, 2018. | 4 | |  |
| 1. SARIGUL GUDUK, Sukran; KARACA, Nezih. Safety and complications of absorbable threads made of poly‐L‐lactic acid and poly lactide/glycolide: experience with 148 consecutive patients. Journal of Cosmetic Dermatology, v. 17, n. 6, p. 1189-1193, 2018. | 2 | |  |
| 1. HARII, Kiyonori; KAWASHIMA, Makoto. A double-blind, randomized, placebo-controlled, two-dose comparative study of botulinum toxin type A for treating glabellar lines in Japanese subjects. Aesthetic plastic surgery, v. 32, n. 5, p. 724-730, 2008. | 2 | |  |
| 1. HEPPT, M. et al. Fillers and associated side effects. HNO, v. 63, n. 7, p. 472-480, 2015. | 4 | |  |
| 1. SABET-PEYMAN, Esfandiar J.; WOODWARD, Julie A. Complications using intense ultrasound therapy to treat deep dermal facial skin and subcutaneous tissues. Dermatologic Surgery, v. 40, n. 10, p. 1108-1112, 2014. | 1 | |  |
| 1. CARRUTHERS, Jean; CARRUTHERS, Alastair. Complications of botulinum toxin type A. Facial Plastic Surgery Clinics, v. 15, n. 1, p. 51-54, 2007. | 4 | |  |
| 1. HWANG, Catherine J.; CHON, Brian H.; PERRY, Julian D. Blindness After Filler Injection: Mechanism and Treatment. Facial Plastic Surgery Clinics, v. 29, n. 2, p. 359-367, 2021. | 4 | |  |
| 1. JURADO, Sara A.; TOMECKI, Kenneth. Infectious Complications of Cosmetic Procedures: Avoidance and Management. CUTIS, p. 6-10, 2013. | 5 | |  |
| 1. KASHKOULI, Mohsen Bahmani et al. Short-and Long-term Patient Satisfaction and Complications in 650 Endoscopic Forehead Lift Procedures. Ophthalmic Plastic and Reconstructive Surgery, v. 38, n. 2, p. 138-145, 2021. | 2 | |  |
| 1. PALM, Melanie et al. A Randomized Study on PLLA Using Higher Dilution Volume and Immediate Use Following Reconstitution. Journal of Drugs in Dermatology: JDD, v. 20, n. 7, p. 760-766, 2021. | 5 | |  |
| 1. GINAT, D. T.; SCHATZ, C. J. Imaging features of midface injectable fillers and associated complications. American Journal of Neuroradiology, v. 34, n. 8, p. 1488-1495, 2013. | 4 | |  |
| 1. KATO, Kiyoko et al. Increase in the incidence of acute inflammatory reactions to injectable fillers during COVID‐19 era. Journal of Cosmetic Dermatology, 2022. | 2 | |  |
| 1. KLEIN, Arnold William. Complications, adverse reactions, and insights with the use of botulinum toxin. Dermatologic surgery, v. 29, n. 5, p. 549-556, 2003. | 4 | |  |
| 1. LATIMER, P. R. et al. Necrotising fasciitis as a complication of botulinum toxin injection. Eye, v. 12, n. 1, p. 51-53, 1998. | 4 | |  |
| 1. LOMBARDI, T. et al. Orofacial granulomas after injection of cosmetic fillers. Histopathologic and clinical study of 11 cases. Journal of oral pathology & medicine, v. 33, n. 2, p. 115-120, 2004. | 2 | |  |
| 1. MAAMARI, Robi N.; MASSRY, Guy G.; HOLDS, John Bryan. Complications associated with fat grafting to the lower eyelid. Facial Plastic Surgery Clinics, v. 27, n. 4, p. 435-441, 2019. | 2 | |  |
| 1. PENG, Hsien‐Li Peter; PENG, Jui‐Hui. Complications of botulinum toxin injection for masseter hypertrophy: Incidence rate from 2036 treatments and summary of causes and preventions. Journal of cosmetic dermatology, v. 17, n. 1, p. 33-38, 2018. | 1 | |  |
| 1. JONES, Derek et al. A Randomized, Comparator-Controlled Study of HARC for Cheek Augmentation and Correction of Midface Contour Deficiencies. Journal of Drugs in Dermatology: JDD, v. 20, n. 9, p. 949-956, 2021. | 5 | |  |
| 1. MCDOWELL, ALLYN J. Effective practical steps to avoid complications in face-lifting: review of 105 consecutive cases. Plastic and Reconstructive Surgery, v. 50, n. 6, p. 563-572, 1972. | 4 | |  |
| 1. JONES, Derek H. et al. Microcannula injection of large gel particle hyaluronic acid for cheek augmentation and the correction of age-related midface contour deficiencies. Dermatologic Surgery, v. 46, n. 4, p. 465-472, 2020. | 2 | |  |
| 1. SNEISTRUP, Christian; HÖLMICH, Lisbet Rosenkrantz; DAHLSTRØM, Karin. Long-term complications after injection of permanent tissue-fillers to the lips. Ugeskrift for Laeger, v. 171, n. 17, p. 1414-1414, 2009. | 5 | |  |
| 1. NAMIN, Arya et al. Complications in facial esthetic surgery. In: Seminars in Plastic Surgery. Thieme Medical Publishers, Inc., 2020. p. 272-276. | 4 | |  |
| 1. ORLOVA, O. R. et al. Correction of esthetic complications of facial nerve pathology with the use of botulinum toxin type A (Dysport). In: MOVEMENT DISORDERS. DIV JOHN WILEY & SONS INC, 111 RIVER ST, HOBOKEN, NJ 07030 USA: WILEY-LISS, 2007. p. S56-S56. | 1 | |  |
| 1. LI, Dong; SUN, Jiaming; WU, Sufan. A multi‐center comparative efficacy and safety study of two different hyaluronic acid fillers for treatment of nasolabial folds in a Chinese population. Journal of Cosmetic Dermatology, v. 18, n. 3, p. 755-761, 2019. | 2 | |  |
| 1. PHILIPP-DORMSTON, Wolfgang G. et al. Global approaches to the prevention and management of delayed-onset adverse reactions with hyaluronic acid-based fillers. Plastic and Reconstructive Surgery Global Open, v. 8, n. 4, 2020. | 4 | |  |
| 1. PREIBISZ, Lukasz; BOULMÉ, Florence; PAUL LORENC, Z. Barbed Polydioxanone Sutures for Face Recontouring: Six-Month Safety and Effectiveness Data Supported by Objective Markerless Tracking Analysis. Aesthetic Surgery Journal, v. 42, n. 1, p. NP41-NP54, 2022. | 5 | |  |
| 1. DE LACERDA, Davi. Prevention and management of iatrogenic blindness associated with aesthetical filler injections. Dermatologic Therapy, v. 31, n. 6, p. e12722, 2018. | 4 | |  |
| 1. SADICK, Neil S. Overview of complications of nonsurgical facial rejuvenation procedures. Clinics in plastic surgery, v. 28, n. 1, p. 163-176, 2001. | 5 | |  |
| 1. SANCHIS, J. M. et al. Orofacial granulomatosis as an adverse reaction to cosmetic fillers: study of 20 cases. In: ORAL DISEASES. COMMERCE PLACE, 350 MAIN ST, MALDEN 02148, MA USA: WILEY-BLACKWELL PUBLISHING, INC, 2010. p. 550-550. | 5 | |  |
| 1. SCARDOVI, Silvio et al. Clinical study of the efficacy, duration and adverse effects of hyaluronic acid implants in the oral-maxillofacial area. Odontoestomatologia, v. 19, n. 30, 2017. | 2 | |  |
| 1. SHAHRABI-FARAHANI, Shokoufeh et al. Granulomatous foreign body reaction to dermal cosmetic fillers with intraoral migration. Oral Surgery, Oral Medicine, Oral Pathology and Oral Radiology, v. 117, n. 1, p. 105-110, 2014. | 2 | |  |
| 1. SZCZERKOWSKA-DOBOSZ, Aneta et al. Acquired facial lipoatrophy: pathogenesis and therapeutic options. Advances in Dermatology and Allergology/Postępy Dermatologii i Alergologii, v. 32, n. 2, p. 127-133, 2015. | 1 | |  |
| 1. TALEI, Ben. Complications of Injectables in the Perioral Region. Facial Plastic Surgery, v. 35, n. 02, p. 182-192, 2019. | 4 | |  |
| 1. TRUSWELL, William H. Complications in Lower Face Rejuvenation: Avoiding, Minimizing, Recognizing, Dealing with Them, and Helping the Patient through the Process of Fixing the Problems. Facial Plastic Surgery, v. 36, n. 04, p. 462-477, 2020. | 4 | |  |
| 1. WOLLINA, Uwe; GOLDMAN, Alberto. Dermal fillers: facts and controversies. Clinics in dermatology, v. 31, n. 6, p. 731-736, 2013. | 1 | |  |
| 1. KADOUCH, Jonathan A. et al. Complications after facial injections with permanent fillers: important limitations and considerations of MRI evaluation. Aesthetic surgery journal, v. 34, n. 6, p. 913-923, 2014. | 4 | |  |
| 1. THULESEN, Jesper. Iatrogenic vision loss following aesthetic treatment with hyaluronic acid‐containing filler: Every injector should be prepared. Dermatologic Therapy, v. 33, n. 6, p. e13913, 2020. | 2 | |  |
| 1. WADIA, Reena. Complications with injectable facial fillers. British Dental Journal, v. 227, n. 10, p. 885-885, 2019. | 4 | |  |
| 1. YEO, Seung Hun; LEE, Young Bae; HAN, Dong Gil. Early Complications from Absorbable Anchoring Suture Following Thread-Lift for Facial Rejuvenation. Archives of Aesthetic Plastic Surgery, v. 23, n. 1, p. 11-16, 2017. | 3 | |  |
| 1. ZHANG, Fangfei; CHEN, Yan. Lipogranuloma after facial cosmetic procedures. Oral Surgery, Oral Medicine, Oral Pathology and Oral Radiology, v. 123, n. 4, p. e123-e132, 2017. | 2 | |  |
| 1. ZAPPI, Eduardo et al. The long‐term host response to liquid silicone injected during soft tissue augmentation procedures: a microscopic appraisal. Dermatologic surgery, v. 33, p. S186-S192, 2007. | 2 | |  |
| 1. DALEY, Tom et al. Oral lesions associated with injected hydroxyapatite cosmetic filler. Oral surgery, oral medicine, oral pathology and oral radiology, v. 114, n. 1, p. 107-111, 2012. | 2 | |  |
| 1. RAYESS, Hani M. et al. A cross-sectional analysis of adverse events and litigation for injectable fillers. JAMA facial plastic surgery, v. 20, n. 3, p. 207-214, 2018. | 2 | |  |
| 1. THOMPSON, DENNIS P.; ASHLEY, FRANKLIN L. Face-lift complications: a study of 922 cases performed in a 6-year period. Plastic and Reconstructive Surgery, v. 61, n. 1, p. 40-49, 1978. | 5 | |  |
| 1. WILSON, Monique Vanaman; FABI, Sabrina Guillen; GREENE, Ryan. Correction of age-related midface volume loss with low-volume hyaluronic acid filler. JAMA Facial Plastic Surgery, v. 19, n. 2, p. 88-93, 2017. | 2 | |  |
| 1. NOH, Tai Kyung et al. Effects of highly concentrated hyaluronic acid filler on nasolabial fold correction: a 24-month extension study. Journal of Dermatological Treatment, v. 27, n. 6, p. 510-514, 2016. | 4 | |  |
| 1. WEINBERG, Michael J.; SOLISH, Nowell. Complications of hyaluronic acid fillers. Facial Plastic Surgery, v. 25, n. 05, p. 324-328, 2009. | 4 | |  |
| 1. MARTIN, Lisette HC; HANKINSON, Paul M.; KHURRAM, Syed A. Beauty is only mucosa deep: a retrospective analysis of oral lumps and bumps caused by cosmetic fillers. British Dental Journal, v. 227, n. 4, p. 281-284, 2019. | 2 | |  |
| 1. WOODWARD, Julie; KHAN, Tanya; MARTIN, John. Facial filler complications. Facial Plastic Surgery Clinics, v. 23, n. 4, p. 447-458, 2015. | 4 | |  |
| 1. CASSUTO, Daniel et al. Management of complications caused by permanent fillers in the face: A treatment algorithm. Plastic and Reconstructive Surgery, v. 138, n. 2, p. 215e-227e, 2016. | 2 | |  |
| 1. SCLAFANI, Anthony P.; FAGIEN, Steven. Treatment of injectable soft tissue filler complications. Dermatologic Surgery, v. 35, p. 1672-1680, 2009. | 4 | |  |
| 1. RIEFKOHL, Ronald; KOSANIN, Radoslav; GEORGIADE, Gregory S. Complications of the forehead-brow lift. Aesthetic Plastic Surgery, v. 7, n. 3, p. 135-138, 1983. | 4 | |  |
| 1. BROLY, M. et al. Management of granulomatous foreign body reaction to fillers with methotrexate. Journal of the European Academy of Dermatology and Venereology, v. 34, n. 4, p. 817-820, 2020. | 2 | |  |
| 1. FARHI, David et al. The Emervel French survey: a prospective real-practice descriptive study of 1,822 patients treated for facial rejuvenation with a new hyaluronic acid filler. Journal of Drugs in Dermatology: JDD, v. 12, n. 5, p. e88-93, 2013. | 5 | |  |
| 1. KADOUCH, Jonathan A. et al. Delayed‐onset complications of facial soft tissue augmentation with permanent fillers in 85 patients. Dermatologic Surgery, v. 39, n. 10, p. 1474-1485, 2013. | 3 | |  |
| 1. ROUANET, Cédric et al. Management of vascular complications following facial hyaluronic acid injection: High-dose hyaluronidase protocol: A technical note. Journal of Stomatology, Oral and Maxillofacial Surgery, v. 123, n. 2, p. 262-265, 2022. | 4 | |  |
| 1. GLOGAU, Richard G.; KANE, Michael AC. Effect of injection techniques on the rate of local adverse events in patients implanted with nonanimal hyaluronic acid gel dermal fillers. Dermatologic surgery, v. 34, p. S105-S109, 2008. | 2 | |  |
| 1. GLAICH, Adrienne S.; COHEN, Joel L.; GOLDBERG, Leonard H. Injection necrosis of the glabella: protocol for prevention and treatment after use of dermal fillers. Dermatologic surgery, v. 32, n. 2, p. 276-281, 2006. | 4 | |  |
| 1. ASCHER, Benjamin et al. A 12-month follow-up, randomized comparison of effectiveness and safety of two hyaluronic acid fillers for treatment of severe nasolabial folds. Dermatologic Surgery, v. 43, n. 3, p. 389, 2017. | 2 | |  |
| 1. BACHMANN, F. et al. Adverse reactions caused by consecutive injections of different fillers in the same facial region: risk assessment based on the results from the Injectable Filler Safety study. Journal of the European Academy of Dermatology and Venereology, v. 25, n. 8, p. 902-912, 2011. | 2 | |  |
| 1. HIRSCH, Ranella J.; STIER, Meghan. Complications of soft tissue augmentation. Journal of drugs in dermatology: JDD, v. 7, n. 9, p. 841-845, 2008. | 4 | |  |
| 1. MACK, William P. Complications in periocular rejuvenation. Facial Plastic Surgery Clinics, v. 18, n. 3, p. 435-456, 2010. | 4 | |  |
| 1. DE MELO CARPANEDA, Erick; CARPANEDA, Carlos Augusto. Adverse results with PMMA fillers. Aesthetic plastic surgery, v. 36, n. 4, p. 955-963, 2012. | 2 | |  |
| 1. LEE, Hyejeong; YOON, Kichan; LEE, Munjae. Outcome of facial rejuvenation with polydioxanone thread for Asians. Journal of Cosmetic and Laser Therapy, v. 20, n. 3, p. 189-192, 2018. | 2 | |  |
| 1. RODRIGUES-BARATA, Ana Rita; CAMACHO-MARTÍNEZ, Francisco M. Undesirable effects after treatment with dermal fillers. J Drugs Dermatol, v. 12, n. 4, p. e59-e62, 2013. | 4 | |  |
| 1. ALIJOTAS‐REIG, JAUME et al. Delayed immune‐mediated adverse effects related to polyacrylamide dermal fillers: clinical findings, management, and follow‐up. Dermatologic surgery, v. 35, p. 360-366, 2009. | 2 | |  |
| 1. HEVIA, Oscar. A retrospective review of calcium hydroxylapatite for correction of volume loss in the infraorbital region. Dermatologic surgery, v. 35, n. 10, p. 1487-1494, 2009. | 4 | |  |
| 1. NARINS, Rhoda S. Minimizing adverse events associated with poly‐L‐lactic acid injection. Dermatologic surgery, v. 34, p. S100-S104, 2008. | 4 | |  |
| 1. COLEMAN 3RD, William P. A special issue devoted to filler complications. Dermatologic Surgery: Official Publication for American Society for Dermatologic Surgery [et al.], v. 35, p. 1597-1597, 2009. | 4 | |  |
| 1. HANEKE, Eckart. Adverse effects of fillers and their histopathology. Facial plastic surgery, v. 30, n. 06, p. 599-614, 2014. | 4 | |  |
| 1. KESTEMONT, Philippe et al. Sustained efficacy and high patient satisfaction after cheek enhancement with a new hyaluronic acid dermal filler. Journal of drugs in dermatology: JDD, v. 11, n. 1 Suppl, p. s9-16, 2012. | 5 | |  |
| 1. SADICK, Neil S.; KATZ, Bruce E.; ROY, Deborshi. A multicenter, 47‐month study of safety and efficacy of calcium hydroxylapatite for soft tissue augmentation of nasolabial folds and other areas of the face. Dermatologic surgery, v. 33, p. S122-S127, 2007. | 2 | |  |
| 1. CHRISTENSEN, Lise et al. Adverse reactions to injectable soft tissue permanent fillers. Aesthetic plastic surgery, v. 29, n. 1, p. 34-48, 2005. | 4 | |  |
| 1. BEAUVAIS, Daniel; FERNEINI, Elie M. Complications and litigation associated with injectable facial fillers: a cross-sectional study. Journal of Oral and Maxillofacial Surgery, v. 78, n. 1, p. 133-140, 2020. | 2 | |  |
| 1. FELLER-HEPPT, Gabriele; HANEKE, Eckart; HEPPT, Markus V. Diagnosis and management of filler adverse effects: an algorithm. Facial plastic surgery, v. 30, n. 06, p. 647-655, 2014. | 4 | |  |
| 1. EL-KHALAWANY, Mohamed et al. Dermal filler complications: a clinicopathologic study with a spectrum of histologic reaction patterns. Annals of diagnostic pathology, v. 19, n. 1, p. 10-15, 2015. | 1 | |  |
| 1. LEVY, Lauren L. et al. Complications of minimally invasive cosmetic procedures: prevention and management. Journal of cutaneous and aesthetic surgery, v. 5, n. 2, p. 121, 2012. | 4 | |  |
| 1. AKSENENKO, Irina et al. A Method for Treating Complications Developed Following Countour Correction with Calcium Hydroxylaptite-based Filler. The Journal of Clinical and Aesthetic Dermatology, v. 15, n. 3, p. 38, 2022. | 4 | |  |
| 1. MARUSZA, Wojciech et al. Treatment of late bacterial infections resulting from soft-tissue filler injections. Infection and Drug Resistance, v. 12, p. 469, 2019. | 4 | |  |
| 1. GLADSTONE, Hayes B.; COHEN, Joel L. Adverse effects when injecting facial fillers. In: Seminars in cutaneous medicine and surgery. WB Saunders, 2007. p. 34-39. | 7 | |  |
| 1. WOLTERS, Marianne; LAMPE, Hermann. Prospective multicenter study for evaluation of safety, efficacy, and esthetic results of cross‐linked polyacrylamide hydrogel in 81 patients. Dermatologic surgery, v. 35, p. 338-343, 2009. | 2 | |  |
| 1. HEXSEL, Doris; DE MORAIS, Marina Resener. Management of complications of injectable silicone. Facial Plastic Surgery, v. 30, n. 06, p. 623-627, 2014. | 4 | |  |
| 1. ELKWOOD, Andrew et al. National plastic surgery survey: brow lifting techniques and complications. Plastic and reconstructive surgery, v. 108, n. 7, p. 2143-50; discussion 2151, 2001. | 4 | |  |
| 1. LEE, Sang-Chang et al. Inflammatory granuloma caused by injectable soft tissue filler (Artecoll). Journal of the Korean Association of Oral and Maxillofacial Surgeons, v. 39, n. 4, p. 193, 2013. | 4 | |  |
| 1. PHILIPP‐DORMSTON, W. G. et al. Consensus statement on prevention and management of adverse effects following rejuvenation procedures with hyaluronic acid‐based fillers. Journal of the European Academy of Dermatology and Venereology, v. 31, n. 7, p. 1088-1095, 2017. | 4 | |  |
| 1. SULAMANIDZE, M.; SULAMANIDZE, G. Facial lifting with aptos methods. Journal of cutaneous and aesthetic surgery, v. 1, n. 1, p. 7, 2008. | 4 | |  |
| 1. JONES, Barry M.; GROVER, Rajiv. Reducing complications in cervicofacial rhytidectomy by tumescent infiltration: a comparative trial evaluating 678 consecutive face lifts. Plastic and reconstructive surgery, v. 113, n. 1, p. 398-403, 2004. | 2 | |  |
| 1. PARK, Tae Hwan; SEO, Sang Won; WHANG, Kwi Whan. Facial rejuvenation with fine-barbed threads: the simple Miz lift. Aesthetic plastic surgery, v. 38, n. 1, p. 69-74, 2014. | 2 | |  |
| 1. LOWE, Nicholas J. et al. Hyaluronic acid skin fillers: adverse reactions and skin testing. Journal of the American Academy of Dermatology, v. 45, n. 6, p. 930-933, 2001. | 1 | |  |
| 1. LIU, H. L.; CHEUNG, W. Y. Complications of polyacrylamide hydrogel (PAAG) injection in facial augmentation. Journal of Plastic, Reconstructive & Aesthetic Surgery, v. 63, n. 1, p. e9-e12, 2010. | 7 | |  |
| 1. HUH, Chang-Hun et al. A randomized, active-controlled, 52-week study of hyaluronic acid fillers for anteromedial malar region augmentation. Plastic and Reconstructive Surgery Global Open, v. 8, n. 2, 2020. | 2 | |  |
| 1. VOY, E. D.; KOBERG, W. Complications and dangers in face and neck lifting. Fortschritte der Kiefer-und Gesichts-chirurgie, v. 34, p. 14-16, 1989. | 5 | |  |
| 1. RACHEL, John D.; LACK, Edward B.; LARSON, Bridget. Incidence of complications and early recurrence in 29 patients after facial rejuvenation with barbed suture lifting. Dermatologic surgery, v. 36, n. 3, p. 348-354, 2010. | 3 | |  |
| 1. BECKER, Ferdinand F.; CASTELLANO, Richard D. Safety of face-lifts in the older patient. Archives of Facial Plastic Surgery, v. 6, n. 5, p. 311-314, 2004. | 2 | |  |
| 1. KHAN, Galina et al. Combined press cog type and cog PDO threads in comparison with the cog PDO threads in facial rejuvenation. Journal of Cosmetic Dermatology, v. 20, n. 10, p. 3294-3298, 2021. | 2 | |  |
| 1. NEWMAN, James. Safety and efficacy of midface-lifts with an absorbable soft tissue suspension device. Archives of Facial Plastic Surgery, 2006. | 2 | |  |
| 1. DAI, Xia et al. Safety and effectiveness of hyaluronic acid dermal filler in correction of moderate-to-severe nasolabial folds in Chinese subjects. Clinical, Cosmetic and Investigational Dermatology, v. 12, p. 57, 2019. | 4 | |  |
| 1. GARVEY, Patrick B.; RICCIARDELLI, Edward J.; GAMPPER, Thomas. Outcomes in threadlift for facial rejuvenation. Annals of plastic surgery, v. 62, n. 5, p. 482-485, 2009. | 3 | |  |
| 1. JUNKINS-HOPKINS, Jacqueline M. Filler complications. Journal of the American Academy of Dermatology, v. 63, n. 4, p. 703-705, 2010. | 4 | |  |
| 1. MORADI, Amir; SHIRAZI, Azadeh; MORADI, Jeanette. A 12-month, prospective, evaluator-blinded study of small gel particle hyaluronic acid filler in the correction of temporal fossa volume loss. Journal of Drugs in Dermatology: JDD, v. 12, n. 4, p. 470-475, 2013. | 5 | |  |
| 1. VACHIRAMON, Vasanop et al. Accuracy of a high‐intensity focused ultrasound device with and without real‐time visualization system in face and neck treatment of skin laxity. Journal of Cosmetic Dermatology, v. 20, n. 1, p. 132-137, 2021. | 1 | |  |
| 1. ASCHER, Benjamin et al. Botulinum toxin A in the treatment of glabellar lines: scheduling the next injection. Aesthetic Surgery Journal, v. 25, n. 4, p. 365-375, 2005. | 7 | |  |
| 1. ZHONG, Yehong et al. A single-blinded prospective study on using botulinum toxin type A for reducing alar mobility. Aesthetic Surgery Journal, v. 42, n. 5, p. 460-469, 2022. | 5 | |  |
| 1. MYUNG, Yujin; JUNG, Chinkoo. Mini-midface lift using polydioxanone cog threads. Plastic and Reconstructive Surgery Global Open, v. 8, n. 6, 2020. | 2 | |  |
| 1. SOLOMON, Philip et al. Facial Soft Tissue Augmentation With Bellafill: A Review of 4 Years of Clinical Experience in 212 Patients. Plastic Surgery, v. 29, n. 2, p. 98-102, 2021. | 3 | |  |
| 1. CHIRICO, Fabrizio et al. Non-surgical touch-up with hyaluronic acid fillers following facial reconstructive surgery. Applied Sciences, v. 11, n. 16, p. 7507, 2021. | 3 | |  |
| 1. SHALMON, Dana et al. Management patterns of delayed inflammatory reactions to hyaluronic acid dermal fillers: an online survey in Israel. Clinical, Cosmetic and Investigational Dermatology, v. 13, p. 345, 2020. | 2 | |  |
| 1. BLANCHARD, Jessica et al. Complications of self-injected facial fillers: a treatment conundrum in the UK. Case Reports in surgery, v. 2019, 2019. | 4 | |  |
| 1. MARUSZA, Wojciech et al. Treatment of late bacterial infections resulting from soft-tissue filler injections. Infection and Drug Resistance, v. 12, p. 469, 2019. | 7 | |  |
| 1. Removal of Polyacrylamide Gel (Aquamid®) from the Lip as a Solution for Late-Onset Complications: Our 8-Year Experience | 2 | |  |
| 1. RAUSO, Raffaele et al. Safety and early satisfaction assessment of patients seeking nonsurgical rhinoplasty with filler. Journal of Cutaneous and Aesthetic Surgery, v. 10, n. 4, p. 207, 2017. | 4 | |  |
| 1. ORTIZ, Arisa E. et al. Analysis of US Food and Drug Administration data on soft-tissue filler complications. Dermatologic Surgery, v. 46, n. 7, p. 958-961, 2020. | 5 | |  |
| 1. INNOCENTI, Alessandro; AMODEO, Chiara Andretto; CIANCIO, Francesco. Wide-undermining neck liposuction: tips and tricks for good results. Aesthetic plastic surgery, v. 38, n. 4, p. 662-669, 2014. | 4 | |  |
| 1. PARK, Tae Hwan; SEO, Sang Won; WHANG, Kwi Whan. Facial rejuvenation with fine-barbed threads: the simple Miz lift. Aesthetic plastic surgery, v. 38, n. 1, p. 69-74, 2014. | 4 | |  |
| 1. EDWARDS, Paul C.; FANTASIA, John E. Review of long-term adverse effects associated with the use of chemically-modified animal and nonanimal source hyaluronic acid dermal fillers. Clinical Interventions in Aging, v. 2, n. 4, p. 509, 2007. | 4 | |  |
| 1. CHRISTENSEN, Lise et al. Adverse reactions to injectable soft tissue permanent fillers. Aesthetic plastic surgery, v. 29, n. 1, p. 34-48, 2005. | 4 | |  |
| 1. LEW, Helen et al. Effect of botulinum toxin A on facial wrinkle lines in Koreans. Ophthalmologica, v. 216, n. 1, p. 50-54, 2002. | 4 | |  |
| 1. VARTANIAN, A. John; DAYAN, Steven H. Complications of botulinum toxin A use in facial rejuvenation. Facial Plastic Surgery Clinics, v. 11, n. 4, p. 483-492, 2003. | 4 | |  |
| 1. JUNKINS-HOPKINS, Jacqueline M. Filler complications. Journal of the American Academy of Dermatology, v. 63, n. 4, p. 703-705, 2010. | 5 | |  |
| 1. WOLLINA, Uwe; KONRAD, Helga. Managing adverse events associated with botulinum toxin type A. American journal of clinical dermatology, v. 6, n. 3, p. 141-150, 2005. | 4 | |  |
| 1. NETTAR, Kartik; MAAS, Corey. Facial filler and neurotoxin complications. Facial Plastic Surgery, v. 28, n. 03, p. 288-293, 2012. | 4 | |  |
| 1. CARRUTHERS, Jean; CARRUTHERS, Alastair. Complications of botulinum toxin type A. Facial Plastic Surgery Clinics, v. 15, n. 1, p. 51-54, 2007. | 4 | |  |
| 1. IANHEZ, Mayra; SOUZA, Marcela, B.; MIOT, Hélio A. Frequency of Complications of Aesthetic Facial Fillers in Brazil. Plast Reconstr Surg, v. 149; n. 3, p.599e-601e, 2022. | 2/5 | |  |
| 1. ORTIZ, Arisa E. et al. Analysis of US Food and Drug Administration data on soft-tissue filler complications. Dermatologic Surgery, v. 46, n. 7, p. 958-961, 2020. | 2 | |  |
| 1. CASSUTO, Daniel et al. Management of complications caused by permanent fillers in the face: A treatment algorithm. Plastic and Reconstructive Surgery, v. 138, n. 2, p. 215e-227e, 2016. | 7 | |  |
| 1. KIM, Byung Wook et al. Adverse events associated with botulinum toxin injection: a multidepartment, retrospective study of 5310 treatments administered to 1819 patients. Journal of dermatological treatment, v. 25, n. 4, p. 331-336, 2014. | 1 | |  |
| 1. NIAMTU III, Joseph. Complications in fillers and Botox. Oral and maxillofacial surgery clinics of North America, v. 21, n. 1, p. 13-21, 2009. | 4 | |  |
| 1. BERTOSSI, Dario et al. Non surgical facial reshaping using MD Codes. Journal of cosmetic dermatology, v. 19, n. 9, p. 2219-2228, 2020. | 3 | |  |
| 1. KEEN, Monte et al. Botulinum toxin A for hyperkinetic facial lines: results of a double-blind, placebo-controlled study. Plastic and reconstructive surgery, v. 94, n. 1, p. 94-99, 1994. | 4 | |  |
| 1. ROBATI, Reza M.; MOEINEDDIN, Fatemeh; ALMASI-NASRABADI, Mina. The risk of skin necrosis following hyaluronic acid filler injection in patients with a history of cosmetic rhinoplasty. Aesthetic surgery journal, v. 38, n. 8, p. 883-888, 2018. | 2 | |  |
| 1. KALANTAR-HORMOZI, Abdolijalil; MOZAFARI, Naser; RASTI, Mehdi. Adverse effects after use of polyacrylamide gel as a facial soft tissue filler. Aesthetic Surgery Journal, v. 28, n. 2, p. 139-142, 2008. | 4 | |  |
| 1. XI, Wenjing et al. The injection for the lower eyelid retraction: a mechanical analysis of the lifting effect of the hyaluronic acid. Aesthetic Plastic Surgery, v. 43, n. 5, p. 1310-1317, 2019. | 4 | |  |
| 1. DAI, Xia et al. Safety and effectiveness of hyaluronic acid dermal filler in correction of moderate-to-severe nasolabial folds in Chinese subjects. Clinical, Cosmetic and Investigational Dermatology, v. 12, p. 57, 2019. | 4 | |  |
| 1. YAZDANPARAST, Taraneh et al. Assessment of the efficacy and safety of hyaluronic acid gel injection in the restoration of fullness of the upper lips. Journal of cutaneous and aesthetic surgery, v. 10, n. 2, p. 101, 2017. | 4 | |  |
| 1. CALLAN, Peter et al. Efficacy and safety of a hyaluronic acid filler in subjects treated for correction of midface volume deficiency: a 24 month study. Clinical, Cosmetic and Investigational Dermatology, v. 6, p. 81, 2013. | 2 | |  |
| 1. ECCLESTON, David; MURPHY, Diane K. Juvéderm® Volbella™ in the perioral area: a 12-month prospective, multicenter, open-label study. Clinical, Cosmetic and Investigational Dermatology, v. 5, p. 167, 2012. | 2 | |  |
| 1. DUCIC, Yadranko; ADELSON, Robert. Use of the endoscopic forehead-lift to improve brow position in persistent facial paralysis. Archives of facial plastic surgery, v. 7, n. 1, p. 51-54, 2005. | 4 | |  |
| 1. WATTANAKRAI, Kamol; CHIEMCHAISRI, Nattawut; WATTANAKRAI, Penpun. Mesh suspension thread for facial rejuvenation. Aesthetic Plastic Surgery, v. 44, n. 3, p. 766-774, 2020. | 2 | |  |
| 1. PARK, Kyu Hyung et al. Iatrogenic occlusion of the ophthalmic artery after cosmetic facial filler injections: a national survey by the Korean Retina Society. JAMA ophthalmology, v. 132, n. 6, p. 714-723, 2014. | 2 | |  |
| 1. ZHANG, Li-xia et al. Evaluation of intraarterial thrombolysis in treatment of cosmetic facial filler-related ophthalmic artery occlusion. Plastic and Reconstructive Surgery, v. 145, n. 1, p. 42e-50e, 2020. | 2 | |  |
| 1. PATEL, Sagar; KRIDEL, Russell. Current trends in management of submental liposis: a pooled analysis and survey. JAMA Facial Plastic Surgery, v. 20, n. 3, p. 202-206, 2018. | 1 | |  |
| 1. D'EMILIO, Roberta; ROSATI Giuseppe. Full-face treatment with onabotulinumtoxinA: Results from a single-center study. J Cosmet Dermatol, v. 19, n. 4, p. 809-816, 2020. | 11 | |  |
| 1. FAN, Xing et al. Safety and efficacy evaluation of botulinum toxin an injection by FITTER® microneedle to treat medial lower eyelid wrinkles. J Cosmet Dermatol, v. 23, n.4, p. 1253-1258, 2024. | 11 | |  |
| 1. GONG, Xi et al. Application of Botulinum Toxin at the Yonsei Point for the Treatment of Gummy Smile: A Randomized Controlled Trial. Plast Reconstr Surg, v. 153, n. 4, p. 711e-721e, 2024. | 11 | |  |
| 1. HEXSEL, Doris; VALENTE-BEZERRA, Indira; MOSENA, Gabriela; OAKIM MOURAO, Maria A; FABRIS, Vitor C. Subjective and Objective Measurements of the Facial Effects of Microdoses of Botulinum Toxin. Dermatol Pract Concept, v. 13, n. 3, p. e2023168, 2023. | 11 | |  |
| 1. HEXSEL, Doris et al. Efficacy, Safety, and Subject Satisfaction After AbobotulinumtoxinA Treatment of Upper Facial Lines. Dermatol Surg, v. 44, n. 12, p. 1555-1564, 2018. | 11 | |  |
| 1. HUA, Tianzhen et al. Is Vibration Anesthesia Effective and Safe for Pain Reduction in Botulinum Toxin Injection? A Randomized Split-Face Controlled Trial and Cadaver Experiment. Aesthet Surg J, p. sjae231, 2024 | 11 | |  |
| 1. LIU, Xuanjun et al. Efficacy and Safety of Hyaluronic Acid Combined with Botulinum Toxin Type A in the Treatment of Midcheek Groove: A Prospective Study. Aesthetic Plast Surg, Epub ahead of print, 2024. | 11 | |  |
| 1. ALBALAT, Waleed; GHONEMY, Soheir; SALEH, Ayat; ELRADI, Mona. Microneedling combined with botulinum toxin-A versus microneedling combined with platelet-rich plasma in treatment of atrophic acne scars: a comparative split face study. Arch Dermatol Res, v. 315, n. 4, p. 839-846, 2023. Erratum in: Arch Dermatol Res, v. 315, n. 10, p. 3007-3008, 2023. | 13 | |  |
| 1. ÖZTÜRK, Bilkay Ö; YEĞIN, Mehmet E; BILKAY, Ufuk. Thread-Filler: A Standardized Combination Therapy. J Craniofac Surg, v. 36, n. 1, 177-181, 2025. | 13 | |  |
| 1. WANG, Ru-Jie; WANG, Ying; WU, Jin-Fang; SI, Ting-Ting. Clinical effect of botulinum toxin type A combined with autologous fat grafting in patients with nasolabial fold depression. World J Clin Cases, v. 12, n.22, p. 4973-4982, 2024. | 13 | |  |
| 1. OZDEMIR CETINKAYA, Pinar et al. Functional and esthetic effects of botulinum toxin injection into the masseter muscles: evaluation of 80 patients from a dermatological perspective. Int J Dermatol, v. 64, n. 1, p. 149-154, 2025. | 1 | |  |
| 1. DASTGHEIB, Mani et al. Investigating the impact of added Profhilo mesogel to subcision versus subcision monotherapy in treating acne scars; a single-blinded, split-face randomized trial. J Cosmet Dermatol, v. 23, n. 6, p. 1992-2000, 2024. | 1 | |  |
| 1. GENNAI, Alessandro et al. Guided Superficial Enhanced Fluid Fat Injection (SEFFI) Procedures for Facial Rejuvenation: An Italian Multicenter Retrospective Case Report. Clin Pract, v. 13, n. 4, p. 924-943, 2023. | 1 | |  |
| 1. HE, Anqi et al. Clinical Efficacy of Mechanical Micronized Fat-Assisted Fat Grafting on Temporal Depression. Ann Plast Surg, v. 93, n. 2S Suppl 1, p. S64-S68, 2024. | 1 | |  |
| 1. JIA, Xinyu; LI Ming; LI, Facheng; YIN, Bo. Microliposuction and Radiofrequency Combined With Fat Grating as a New Method for Hybrid-Type Nasolabial Folds. J Craniofac Surg, v.35, n. 7, p. 2150-2155, 2024. | 1 | |  |
| 1. MOVASSAGHI, Kiya; CHECK, Janna; GOUGOUTAS, Alexander. The U-SMASectomy Facelift. Plast Reconstr Surg, Epub ahead of print, 2024. | 1 | |  |
| 1. SERGESKETTER, Amanda R et al. Tracking Complications and Unplanned Healthcare Utilization in Aesthetic Surgery: An Analysis of 214,504 Patients Using the TOPS Database. Plast Reconstr Surg, v. 151, n. 6 ,p. 1169-1178, 2023. | 1 | |  |
| 1. ŞIRINOGLU, Hakan; ERGÜN TATAR, Burak; GÜVERCIN, Emre. Long Terms Results of Temporal Facelift: 6 Years of Experience in 250 Cases. J Craniofac Surg,Epub ahead of print, 2024. | 1 | |  |
| 1. SPARAVIGNA, Adele; GRIMOLIZZI, Franco; CIGNI, Clara; LUALDI, Roberto; BELLIA, Gilberto. Dual-Plane Treatment With Highly Concentrated Hybrid Cooperative Complexes of Hyaluronans for Facial Atrophic Acne Scars. Dermatol Surg, v. 51, n. 2, p. 152-156, 2025. | 1 | |  |
| 1. WILL, P A et al. Treatment of retracted, postsurgical scars and reduction of locoregional edema using a combined three-dimensional approach of liposuction lipofilling, dissecting cannulas, and suspension sutures. Eur J Plast Surg, v. 46, p. 1357–1367, 2023. | 1 | |  |
| 1. WOLF, Yoram; SELINGER, Rami; SKOROCHOD, Ron; MITZ, Vladimir. Micro-face-lift: A novel biplanar, composite, less-invasive procedure. J Cutan Aesthet Surg, v, 17, n. 1, p. 60-65, 2024. | 1 | |  |
| 1. XIONG, Chenlu et al. Complications Following Facial Injection of Growth Factor Solution. Aesthetic Plast Surg, v. 47, n. 2, p. 612-621, 2023. | 1 | |  |
| 1. YIN, Yue; LI, Tong; WANG, Chufang; MA, Qiaoxin; FAN, Xing. Treatment of Facial Flaccidity and Sagging after Botulinum Toxin A Injection into the Masseter. Plast Reconstr Surg, v. 151, n. 3, p. 521-525, 2023. | 1 | |  |
| 1. ZHANG, Lei et al. Long-term Prognosis of Vision Loss Caused by Facial Hyaluronic Acid Injections and the Potential Approaches to Address This Catastrophic Event. Aesthet Surg J, v. 43, n. 4, p. 484-493, 2023. | 1 | |  |
| 1. FAKIH-GOMEZ, Nabil; KADOUCH, Jonathan; FELICE, Fernando; HAYKAL, Diala; MUÑOZ-GONZALEZ, Cristina. The Hybrid Filler Technique: A 5-Year Retrospective Analysis. Aesthetic Plast Surg, Epub ahead of print, 2024. | 3 | |  |
| 1. CAVALLINI, Maurizio; CASASCO, Andrea; FERRARA, Fulvio; RAICHI, Mauro; SPINELLI, Giuseppina. Hyaluronic Acid Fillers, Needle Contamination by Fastidious Microorganisms, and Risk of Complications. Dermatol Surg, v. 49, n. 2, p. 161-163, 2023. | 4 | |  |
| 1. HONG, Gi-Woong et al. Adverse Effects Associated with Dermal Filler Treatments: Part II Vascular Complication. Diagnostics (Basel), v. 14, n. 14, p. 1555, 2024. | 4 | |  |
| 1. IMEN, Mehri T. Delayed infectious reactions of dermal filler injections in the face: Causes and management. J Stomatol Oral Maxillofac Surg, v. 124, n. 2, p. 101334, 2023. | 4 | |  |
| 1. FENG, Ge et al. A Two-Center, Prospective, Randomized Controlled Trial to Evaluate the Efficacy and Safety of and Satisfaction with Different Methods of ART FILLER® UNIVERSAL Injection for Correcting Moderate to Severe Nasolabial Folds in Chinese Individuals. Aesthetic Plast Surg, v. 47, n. 4, p. 1550-1559, 2023. | 4 | |  |
| 1. PARIKH, Alomi O; CONGER, Jordan R; SIBUG SABER, Maria E; SAMIMI, David; BURNSTINE, Michael A. Multiple Cases of Facial Disfigurement From Filler Use and One Injector. Ophthalmic Plast Reconstr Surg, v. 39, n. 4, p. 366-369, 2023. | 4 | |  |
| 1. SOARES, Danny J; HYNES, Stephanie D; YI, Christina H; SHAH-DESAI, Sabrina; IRVING, Steven C. Cosmetic Filler-Induced Vascular Occlusion: A Rising Threat Presenting to Emergency Departments. Ann Emerg Med, v. 83, n. 1, p. 59-67, 2024. | 4 | |  |
| 1. STEENEN, Serge A et al. Head-to-head comparison of 4 hyaluronic acid dermal fillers for lip augmentation: A multicenter randomized, quadruple-blind, controlled clinical trial. J Am Acad Dermatol, v. 88, n. 4, p. 932-935, 2023. | 4 | |  |
| 1. STEENEN, Serge A; BAULAND, Constantijn G; DE LANGE, Jan; VAN DER LEI, Berend. Complications After Botulinum Neurotoxin Type A and Dermal Filler Injections: Data From a Large Retrospective Cohort Study. Aesthet Surg J, v. 43, n. 1, p. 56-63, 2023. | 4 | |  |
| 1. WOODWARD, Julie et al. Infraorbital Hollow Rejuvenation: Considerations, Complications, and the Contributions of Midface Volumization. Aesthet Surg J Open Forum, v. 5, p. ojad016, 2023. | 4 | |  |
| 1. Zaccaria Giovanna, Dotti Alessandro, Benanti Elisa, Vigliarolo Camilla, Vaienti Luca. A treatment algorithm for hyaluronic acid filler related complications of the face. J Plast Reconstr Aesthet Surg, v. 91, p. 207-217, 2024. | 4 | |  |
| 1. SARIGUL GUDUK, Sukran; KARACA, Nezih. Safety and complications of absorbable threads made of poly-L-lactic acid and poly lactide/glycolide: Experience with 148 consecutive patients. J Cosmet Dermatol, v. 17, n. 6, p. 1189-1193, 2018. | 8 | |  |
| 1. SAVOIA, Antonella; ACCARDO, Ciro; VANNINI, Fulvio; DI PASQUALE, Basso; BALDI, Alfonso. Outcomes in thread lift for facial rejuvenation: a study performed with happy lift™ revitalizing. Dermatol Ther (Heidelb), v. 4, n. 1, p. 103-14, 2014 | 8 | |  |
| 1. SINGH, Sukbir et al. Real-World Study of Definisse Threads for Facial Reshaping in Indian Patients: REDEFINE FACE Study. Cureus, v. 16, n. 4, p. e58258, 2024. | 8 | |  |
| 1. SULAMANIDZE, Marlen; SULAMANIDZE, George; VOZDVIZHENSKY, Ivan; SULAMANIDZE; Constatin. Avoiding complications with Aptos sutures. Aesthet Surg J, v. 31, n. 8, p. 863-73, 2011. | 8 | |  |
| 1. ALEXIADES, Macrene et al. A Randomized, Multicenter, Evaluator-blind Study to Evaluate the Safety and Effectiveness of VYC-12L Treatment for Skin Quality Improvements. Dermatol Surg, v. 49, n. 7, p. 682-688, 2023. | 10 | |  |
| 1. BARONE, Mauro; SALZILLO, Rosa; DE BERNARDIS, Riccardo; CARRUTHERS, Jean; PERSICHETTI, Paolo. Three Key Submuscular Points for Nonsurgical Rejuvenation of the Midface in Caucasian Patients: A Methodological Approach Using Injectable Hyaluronic Acid Fillers. Aesthetic Plast Surg, v. 48, n. 16, p. 3154-3162, 2024. | 10 | |  |
| 1. BEER, Kenneth; BIESMAN, Brian; COX, Sue E; SMITH, Stacy; PICAULT, Laura; TREVIDIC, Patrick. Efficacy and Safety of Resilient Hyaluronic Acid Fillers Injected with a Cannula: A Randomized, Evaluator-Blinded, Split-Face Controlled Study. Clin Cosmet Investig Dermatol, v. 16, p. 959-972, 2023. Erratum in: Clin Cosmet Investig Dermatol, v.16, p. 1763-1764, 2023. | 10 | |  |
| 1. BHOJANI-LYNCH, Tahera; DECKERS, Anne; OHANES, Ohan; POUPARD, Kevin; MAFFERT, Pauline. A Prospective, Observational Registry Study to Evaluate Effectiveness and Safety of Hyaluronic Acid-Based Dermal Fillers in Routine Practice: Interim Analysis Results with One Year of Subject Follow-Up. Clin Cosmet Investig Dermatol, v. 14, p. 1685-1695, 2021 | 10 | |  |
| 1. BIESMAN, Brian S, et al. A Multicenter, Randomized, Evaluator-Blinded Study to Examine the Safety and Effectiveness of Hyaluronic Acid Filler in the Correction of Infraorbital Hollows. Aesthet Surg J, v. 44, n. 9, p. 1001-1013, 2024. | 10 | |  |
| 1. BIESMAN, Brian S; MONTES, Jose R; RADUSKY, Ross C; MERSMANN, Sabine; GRAUL, Virginia W. A Prospective, Multicenter, Evaluator-Blind, Randomized, Controlled Study of Belotero Balance (+), a Hyaluronic Acid Filler With Lidocaine, for Correction of Infraorbital Hollowing in Adults. Aesthet Surg J, v. 44, n. 9, p. 976-986, 2024. | 10 | |  |
| 1. CALOMENI, Mariana et al. Precision of Soft-Tissue Filler Injections: An Ultrasound-Based Verification Study. Aesthet Surg J, v. 43, n. 3, p. 353-361, 2023. | 10 | |  |
| 1. FANIAN, Ferial et al. P. Evaluation of the Performance and Tolerance of the Combination of an HA-based Filler with Tri-Hyal Technology and a Skin Biorevitalizer on Skin Aging Parameters. Clin Cosmet Investig Dermatol, v. 16, p. 1095-1105, 2023 | 10 | |  |
| 1. FANIAN, Ferial et al. A hyaluronic acid-based micro-filler improves superficial wrinkles and skin quality: a randomized prospective controlled multicenter study. J Dermatolog Treat, v. 34, n. 1, p. 2216323, 2023. | 10 | |  |
| 1. MAHMOOD, Faris BJ. The Use of Facial Fillers in Clinical Practice: The Level of Patient Satisfaction and an Overview of Common Clinical Complications. Actas Dermosifiliogr, v. 115, n. 5, p. 458-465, 2024. English, Spanish. | 10 | |  |
| 1. HARB, Ayad; BREWSTER, Colin T. The Nonsurgical Rhinoplasty: A Retrospective Review of 5000 Treatments. Plast Reconstr Surg, v. 145, n. 3, p. 661-667, 2020. | 10 | |  |
| 1. JALALI, Arash. Nonsurgical rhinoplasty using the hyaluronic acid filler VYC-25L: Safety and patient satisfaction in a retrospective analysis of 492 patients. J Cosmet Dermatol, v. 23, n. 2, p. 426-433, 2024. | 10 | |  |
| 1. KESTEMONT, Philippe et al. Long-term efficacy and safety of a hyaluronic acid dermal filler based on Tri-Hyal technology on restoration of midface volume. J Cosmet Dermatol, v. 22, n. 9, p. 2448-2456, 2023. | 10 | |  |
| 1. LÓPEZ, Petra V; GARCÍA, Paloma T; LÓPEZ-PITALÚA, Juan A; PINTO, Hernán. Side effects after hyaluronic acid facial injection in adults during COVID-19 pandemic. J Cosmet Dermatol, v. 22, n. 6, p. 1714-1719, 2023. | 10 | |  |
| 1. MARTÍN, Javier M et al. Superficial versus deep injections of the upper midface-A prospective interventional split-face study. J Cosmet Dermatol, v. 22, n. 11, p. 2940-2949, 2023. | 10 | |  |
| 1. NIKOLIS, Aandreas; ENRIGHT, Kaitlyn M; COTOFANA, Sebastian; NGUYEN, Quynh; SAFRAN, Tyler. Comparative Trial Evaluating a High- Versus Low-Integration Hyaluronic Acid Filler for Contouring the Jawline. Aesthetic Plast Surg, v. 49, n. 1, p. 31-42, 2025. | 10 | |  |
| 1. NIKOLIS, Andreas; BERTUCCI, Vince; HUMPHREY, Shannon; BELEZNAY, Katie; BERNSTEIN, Steven; PRYGOVA, Inna. Effectiveness of hyaluronic acid fillers for cheek augmentation using a treatment guide to choose between products. J Cosmet Dermatol, v. 23, n. 11, p. 3525-3531, 2024. | 10 | |  |
| 1. NISHIKAWA, Ayaka; AIKAWA, Yoshiyuki; KONO, Taro. Current Status of Early Complications Caused by Hyaluronic Acid Fillers: Insights From a Descriptive, Observational Study of 41,775 Cases. Aesthet Surg J, v. 43, n. 8, p. 893-904, 2023. | 10 | |  |
| 1. PARK, Su J; YOO, Kwang H. One-Year Safety Evaluation of New Hyaluronic Acid Fillers (YYS Series): A Prospective, Multicenter, Observational Study. Dermatol Surg, v. 50, n. 8, p. 731-738, 2024. | 10 | |  |
| 1. REN, Rongxin et al. Restoring long-lasting midface volume in the Asian face with a hyaluronic acid filler: A randomized controlled multicenter study. J Cosmet Dermatol, v. 23, n. 6, p. 1985-1991, 2024. | 10 | |  |
| 1. RIVKIN, Alexander et al. Safe and Effective Restoration of Jawline Definition With Hyaluronic Acid Injectable Gel VYC-25L: Results From a Randomized Controlled Study. Aesthet Surg J, v. 44, n. 12, p. 1341-1349, 2024. | 10 | |  |
| 1. SIQUIER-DAMETO, Gabriel; SALTI, Giovanni; RHARBAOUI, Siham; MALGAPO, Dennis MH; INNOCENTI, Silvia; MANNI, Martina. A 12-Month Analysis of the Dermatologic Effects and Durability of Midface Volume Correction With DEF CL Volumizing Filler in a Prospective, Single-Center Study. Dermatol Surg, v. 50, n. 12, p. 1131-1136, 2024. | 10 | |  |
| 1. SMITH, Scott W; MASSEY, Blaine L; HALL, Michael B; BUCKINGHAM, Edward D. A Prospective Open-Label Study for Treatment of Infraorbital Hollows Using a Volumizing Hyaluronic Acid Filler. Facial Plast Surg, p. 40, n. 3, p. 363-369, 2024. | 10 | |  |
| 1. SPARAVIGNA, Adele; GRIMOLIZZI, Franco; CIGNI, Cigni; LUALDI, Roberto; BELLIA, Gilberto. Efficacy and tolerability of Profhilo® Structura intended to restore lateral cheek fat compartment: An observational pilot study. Health Sci Rep, v. 7, n. 1, p. e1743, 2024. | 10 | |  |
| 1. TODDE, Salvatore; SVOLACCHIA, Fabiano; SVOLACCHIA, Lorenzo; GIUZIO, Federica; PANDA, Sameer K; FERRARO, Giuseppe A. Performance and Safety of Amino-Acid- and Hydroxyapatite Enriched-Hyaluronic Acid Intradermal Gel in Facial Skin Defects. Medicina (Kaunas), v. 60, n. 7, p. 1121, 2024. | 10 | |  |
| 1. URDIALES-GÁLVEZ, Fernando; BRAZ, André; CAVALLINI, Maurizio. Facial rejuvenation with the new hybrid filler HArmonyCa™: Clinical and aesthetic outcomes assessed by 2D and 3D photographs, ultrasound, and elastography. J Cosmet Dermatol, v. 22, n. 8, p. 2186-2197, 2023. | 10 | |  |
| 1. BACHMANN, Frank; ERDMANN, Ricardo; HARTMANN, Vanessa; WIEST, Luitgard; RZANY, Berthold. The spectrum of adverse reactions after treatment with injectable fillers in the glabellar region: results from the Injectable Filler Safety Study. Dermatol Surg, v. 35, Suppl 2, p. 1629-34, 2009. | 12 | |  |
| 1. KAUFMAN GOLDBERG, Tal; MCGONAGLE, Elizabeth R; HADLOCK, Tessa A. Post-Face Lift Facial Paralysis: A 20-Year Experience. Plast Reconstr Surg, v. 154, n. 4, p. 748-758, 2024. | 12 | |  |
| 1. GRIPPAUDO, Francesca R; DI GIROLAMO, Marco; MATTEI, Mauro; PUCCI, Eugenio; GRIPPAUDO, Cristina. Diagnosis and management of dermal filler complications in the perioral region. J Cosmet Laser Ther, v. 16, n. 5, p. 246-52, 2014. | 12 | |  |
| 1. LI, Yi-Lin; LI, Ze-Hui; CHEN, Xue-Ying; XING, Wen-Shan; HU, Jin-Tian. Facial Thread Lifting Complications in China: Analysis and Treatment. Plast Reconstr Surg Glob Open, v. 9, n. 9, p. e3820, 2021. | 12 | |  |
| 1. PARK, Tae-Han; SEO, Sang-Won; KIM, June-Kyu; CHANG, Choong-Hyun. Clinical experience with hyaluronic acid-filler complications. J Plast Reconstr Aesthet Surg, v. 64, n. 7, p. 892-6, 2011. | 12 | |  |
| 1. PIRES, Fabio R et al. Oral/Perioral Reactions to Injectable Soft Tissue Fillers: A Clinicopathological Multicentric Study. Oral Dis, Epub ahead of print, 2024. | 12 | |  |
| 1. ROSENDY, Gabriela et al. Adverse Reactions Associated with Dermal Fillers in the Oral and Maxillofacial Region: A Venezuelan Experience. Head Neck Pathol, v. 17, n. 3, p. 631-637, 2023. | 12 | |  |
| 1. SUN, Zhong-Sheung et al. Clinical Outcomes of Impending Nasal Skin Necrosis Related to Nose and Nasolabial Fold Augmentation with Hyaluronic Acid Fillers. Plast Reconstr Surg, v. 136, n. 4, p. 434e-441e, 2015. | 12 | |  |
| 1. UTH, Charlotte C; ELBERG, Jens.J; ZACHARIAE, Claus. Complications caused by injection of dermal filler in Danish patients. Eur J Plast Surg, v. 39, p. 441–448, 2016. | 12 | |  |
| 1. YANG, Hong et al. Facial Artery Branch Thrombolysis for Nasal Vascular Embolism Induced by Hyaluronic Acid Injection. Ann Plast Surg, v. 93, n. 6, p. 658-663, 2024. | 12 | |  |
| 1. ANGULO-MANZANEQUE, Gema; BAUS-DOMÍNGUEZ, María; RUIZ-DE-LEÓN-HERNÁNDEZ, Gonzalo; SERRERA-FIGALLO, María Á; TORRES-LAGARES, Daniel; AGUILERA, Fátima S. Evaluation of a New Technique of Gingival Smile Reduction after Hyaluronic Acid Infiltration: A Cohort Study Focusing on Gingival Exposure and Patient-Oriented Outcomes. Dent J (Basel), v. 12, n. 10, 329, 2024. | 2 | |  |
| 1. BUHSEM, Omer. Comparing the Effects of Different Injection Techniques Used in Lip Augmentation on Filler Migration and Patient Satisfaction. Cureus, v. 16, n. 7, p. e64716, 2024. | 2 | |  |
| 1. CHOI, Sun Y et al. A multicenter, double-blind, randomized, parallel-group, active-controlled, phase 3 clinical trial to compare the effectiveness and safety of two botulinum toxin type A formulations for improving moderate to severe glabellar wrinkles in Asians. J Dermatolog Treat, v. 35, n. 1, p. 2359511, 2024. | 2 | |  |
| 1. CHOI, Sun Y; KOH, Young G; YOO, Kwang H; HAN, Hye S; SEOK, Joon; KIM, Beom J. A Randomized, Participant- and Evaluator-Blinded, Matched-Pair, Prospective Study Comparing the Safety and Efficacy Between Polycaprolactone and Polynucleotide Fillers in the Correction of Crow's Feet. J Cosmet Dermatol, v. 24, n. 1, p. e16576, 2025. | 2 | |  |
| 1. COLEMAN, William et al. NivobotulinumtoxinA in the Treatment of Glabellar Lines With or Without Concurrent Treatment of Lateral Canthal Lines in Two Phase 3 Clinical Trials. Aesthet Surg J, sjae233, 2024. | 2 | |  |
| 1. GERMANI, Marcelo; MIRANDA DE SOUZA ALMEIDA, Claudia C; MUÑOZ-LORA, Victor RM. Comparison of 2 Fillers for Lip Injection-A Randomized-Controlled Clinical Trial Assessed by 3D Imaging. Aesthet Surg J Open Forum, v. 6, ojae003, 2024 | 2 | |  |
| 1. GIAMMARIOLI, Giulio; LIBERTI, Alberto. Non-surgical rhinoplasty technique: An innovative approach for nasal reshaping with hyaluronic acid fillers. J Cosmet Dermatol, v. 22, n. 7, p. 2054-2062, 2023. | 2 | |  |
| 1. GONG, Xi et al. Effects of Dose and Injection Site on Gingival Smile Treatment with Botulinum Toxin Type A: A Prospective Study. Plast Reconstr Surg, v. 151, n. 1, p. 56e-67e, 2023. | 2 | |  |
| 1. HARII, Kiyonori; KAWASHIMA, Makoto; FURUYAMA, Nobutaka; LEI, Xiaofang; HOPFINGER, René; LEE, Elizabeth. OnabotulinumtoxinA (Botox) in the Treatment of Crow's Feet Lines in Japanese Subjects. Aesthetic Plast Surg, v. 41, n. 5, p. 1186-1197, 2017. | 2 | |  |
| 1. HEDAYAT, Kamand; EHSANI, Amir H. A Phase III Clinical Study of the Efficacy and Safety of Botulinum Toxin Type A (MASPORT) with DYSPORT for the Treatment of Glabellar Lines. Aesthetic Plast Surg, v. 48, n. 3, p. 324-332, 2024. | 2 | |  |
| 1. JIA, Yao et al. Efficacy and Safety of Type III Collagen Lyophilized Fibers Using Mid-to-Deep Dermal Facial Injections for the Correction of Dynamic Facial Wrinkles. Aesthetic Plast Surg, v. 48, n. 13, p. 2500-2512, 2024. | 2 | |  |
| 1. RODRIGUEZ-CHAVEZ, Nicte; GONZALEZ-MONDRAGÓN, Edric; NAVA-CASTAÑEDA, Angel. Comparative study between the efficacy of prabotulinum toxin-A versus onabotulinum toxin-A for the treatment of upper facial expression lines. J Cosmet Dermatol, v. 23, n. 11, p. 3532-3538, 2024. | 2 | |  |
| 1. SALTI, Giovanni; SIQUIER-DAMETO, Gabriel; RHARBAOUI, Siham; HERNANDEZ MALGAPO, Dennis M; INNOCENTI, Silvia; MANNI, Martina. An Interim 6-Month Analysis of the Dermatologic Effects and Midface Volume Correction With XTR CL Filler in a Prospective, Single-Center Study. Dermatol Surg, v. 49, n. 10, p. 943-948, 2023. | 2 | |  |
| 1. SCARDUA, Natália; ROVARIS, Denize P; MOREIRA, Kelly MS; GUIMARÃES, André LS; SCARDUA, Maria T. Supraperiosteal technique protocol for forehead filling with a mixture of calcium hydroxyapatite and hyaluronic acid: Double-blind, randomized controlled clinical trial. J Cosmet Dermatol, v. 23, n. 12, p. 4116-4122, 2024. | 2 | |  |
| 1. SHENOY, Chaithra; AGRAWAL, Ritu; CHANDRASHEKAR, Byalakere S; LALCHANDANI, Rajesh. Comparison of Safety and Efficacy of Two Brands of Botulinum Toxin A for the Treatment of Lateral Canthal Lines (Crow's Feet): A Split-Face Study. J Cutan Aesthet Surg, v. 16, n. 4, p. 270-278, 2023. | 2 | |  |
| 1. SULYMAN, Omotara et al. Non-surgical rhinoplasty using polydioxanone threads. J Cosmet Dermatol, v. 23, n. 1, p. 199-206, 2024. | 2 | |  |
| 1. SY, Jessica J; WU, Raymond; WAN, Jovian; KIM, Soo-Bin; YI, Kyu-Hoo. The efficacy and safety of neubotulinumtoxinA for the treatment of forehead horizontal lines in Asians - A clinical, prospective, interventional, split-face study. Skin Res Technol, v. 30, n. 4, p. e13644, 2024. | 2 | |  |
| 1. YANG, Bin et al. Clinical efficacy of intradermal type I collagen injections in treating skin photoaging in patients from high-altitude areas. World J Clin Cases, v. 12, n. 16, p. 2713-2721, 2024. | 2 | |  |
| 1. ZERBINATI, Nicola et al. Efficacy and Safety of Neauvia Intense in Correcting Moderate-to-Severe Nasolabial Folds: A Post-Market, Prospective, Open-Label, Single-Centre Study. Clin Cosmet Investig Dermatol, v. 17, p. 1351-1363, 2024. | 2 | |  |
| 1. ALAM, Murad; YOO, Simon S. Technique for calcium hydroxylapatite injection for correction of nasolabial fold depressions. J Am Acad Dermatol, v. 56, n. 2, p. 285-289, 2007. | 9 | |  |
| 1. DAINES, Steven M; WILLIAMS, Edwin F. Complications associated with injectable soft-tissue fillers: a 5-year retrospective review. JAMA Facial Plast Surg, v. 15, n. 3, p. 226-31, 2013. | 9 | |  |
| 1. FAKIH-GOMEZ, Nabil; VERANO-GARCIA, Alba; PORCAR PLANA, Carmen A; MUÑOZ-GONZALEZ, Cristina; KADOUCH, Jonathan. Jawline Sharp Contouring With Hybrid Filler. Aesthetic Plast Surg, v. 49, n. 1, p. 334-340, 2025. | 9 | |  |
| 1. GREEN, Jeremy B et al. Long-term duration and safety of Radiesse (+) for the treatment of jawline. J Cosmet Dermatol, v. 23, n. 10, p. 3202-3209, 2024. | 9 | |  |
| 1. HAN, Woo Y; KIM, Hyeon J; KWON, Rosie; KANG, So M; YON, Dong K. Safety and Efficacy of Poly-L-Lactic Acid Filler (Gana V vs. Sculptra) Injection for Correction of the Nasolabial Fold: A Double-Blind, Non-Inferiority, Randomized, Split-Face Controlled Trial. Aesthetic Plast Surg, v. 47, n. 5, p. 1796-1805, 2023. | 9 | |  |
| 1. HONG, Ji Y; LEE, Yoon H; KIM, Hyun-Jo; PARK, Kui Y. Therapeutic Performance of Needle Injection Versus Needle-Free Jet Injector System for Polynucleotide Filler in Skin Rejuvenation. J Cosmet Dermatol, v. 24, n. 1, p. e16595, 2025. | 9 | |  |
| 1. KALANTAR-HORMOZI, Abdolijalil; MOZAFARI, Naser; RASTI, Mehdi. Adverse effects after use of polyacrylamide gel as a facial soft tissue filler. Aesthet Surg J, v. 28, n. 2, p. 139-142, 2008. | 9 | |  |
| 1. LIAO, Zhi-Feng et al. Cross-linked Sodium Hyaluronate Gel with PLLA-b-PEG Microsphere for Facial Contouring in Chinese: A Retrospective Study. Aesthetic Plast Surg, v. 48, n. 21, p. 4252-4261, 2024. | 9 | |  |
| 1. LIN, Shang-Li; CHRISTEN, Marie-Odile. Polycaprolactone-based dermal filler complications: A retrospective study of 1111 treatments. J Cosmet Dermatol, v. 19, n. 8, p. 1907-1914, 2020. | 9 | |  |
| 1. PAN, Yuyan et al. A Multicenter, Randomized, Double-Blind, Parallel-Grouped, Positive-Controlled, Non-Inferiority Clinical Study to Evaluate the Efficacy and Safety of Injectable Calcium Hydroxylapatite Microsphere Hydrogel Fillers in the Correction of Nasolabial Fold in Chinese Subjects. Aesthetic Plast Surg, Epub ahead of print, 2024. | 9 | |  |
| 1. PROIETTI, Ilaria et al. HArmonyCa™ hybrid filler to restore connective tissue: An Italian real-life retrospective study. J Cosmet Dermatol, v. 23, n. 12, p. 3883-3892, 2024. | 9 | |  |
| 1. REDA-LARI, Abdul. Augmentation of the malar area with polyacrylamide hydrogel: experience with more than 1300 patients. Aesthet Surg J, v. 28, n. 2, p. 131-138, 2008. | 9 | |  |
| 1. RIVKIN, Alexander. Nonsurgical Rhinoplasty Using Injectable Fillers: A Safety Review of 2488 Procedures. Facial Plast Surg Aesthet Med, v. 23, n. 1, p. 6-11, 2021. | 9 | |  |
| 1. VASCONCELOS-BERG, Roberta; REAL, Julia; WENZ, Franziska; AVELAR, Luiz Eduardo T. Safety of the Immediate Reconstitution of Poly-l-Lactic Acid for Facial and Body Treatment-A Multicenter Retrospective Study. J Cosmet Dermatol, v. 23, n. 12, p. 3918-3923, 2024. | 9 | |  |
| 1. YANG, Chin-Yi et al. Evaluation of Collagen Dermal Filler with Lidocaine for the Correction of Nasolabial Folds: A Randomized, Double-Blind, Multicenter Clinical Trial. Clin Cosmet Investig Dermatol, v. 17, p. 1621-1631, 2024. | 9 | |  |
| 1. AVELAR, Rui. Post Hoc Analysis Comparing the Safety and Efficacy of PrabotulinumtoxinA in Millennials and Nonmillennials With Moderate to Severe Glabellar Lines. Dermatol Surg, v. 50, n. 9S, p. 42-47, 2024. | 7 | |  |

(1) Studies involving procedures for non-aesthetic reasons, non-orofacial locations, or procedures other than BoNT-A, HA fillers or non-surgical facelift with absorbable threads.

(2) Studies that did not investigate the frequency of TRAEs related to the specified procedures.

(3) Studies in which TRAEs could not be extracted due to clustering with other aesthetic procedures.

(4) Reviews, case series/reports, protocols, short communications, personal opinions, letters, conference abstracts, book chapters, and in vitro or in vivo animal studies.

(5) Studies for which a full-text copy was not available.

(6) Studies published in other language than English, Spanish, or Portuguese.

(7) Studies with a duplicated sample.

(8) Studies involving thread-lifting procedures that extended to the neck.

(9) Studies on injectable fillers that did not use hyaluronic acid.

(10) Studies on HA fillers that did not include the marionette lines, chin, lips, or nasolabial folds.

(11) Studies on botulinum toxin that did not target the procerus, frontal region/forehead, or lateral canthal lines.

(12) Historical cohort studies in which TRAEs were part of the inclusion criterion.

(13) Studies that combined multiple minimally invasive facial aesthetic procedures within the same treatment period, thereby preventing isolated analysis of individual interventions.
