# Supplementary material for: Adverse Events in Nonsurgical Facial Aesthetic Procedures: A Systematic Review and Meta‐Analysis
Source: Oral Dis. 2025 Oct 5;32(2):384–94. doi: 10.1111/odi.70109 (PMC13077022; doi:10.1111/odi.70109)
Supplement: Supplementary file 5 — Table S4: Treatment‐related adverse events associated with aesthetic hyaluronic acid fillers in the lips, nasolabial folds, marionette lines and chin. Table S5: Treatment‐related adverse events associated with aesthetic use of botulinum toxin type A in the upper face region. Table S6: Treatment‐related adverse events associated with aesthetic nonsurgical facial lifting using absorbable threads. [file ODI-32-384-s005.docx]

**Supplementary Table S4.** Treatment-related adverse events associated with aesthetic hyaluronic acid fillers in the lips, nasolabial folds, marionette lines and chin.

|  | HA fillers | | | | | | | |
| --- | --- | --- | --- | --- | --- | --- | --- | --- |
| Reference | | **Site** | **Sex** | **Age range**  **(years)** | **Patients with**  **TRAEs (n)** | **TRAEs (n)** | **TRAE’s description (n)** | **Management** |
| YAZDANPARAST et al. (2017) | | Upper lips | F: 10 | 28-45 | 10 | 17 | Pain/Tenderness (5), Swelling (1), Bruising (7), Firmness (3), Lump/Bump (1) | Lump/bump: hyaluronidase |
| TAYLOR et al. (2019) | | Lips/perioral | F: 69 M: 3 | 20-79 | 59 | 352 | Swelling (55), Firmness (52), Tenderness (50), Lumps/Bumps (49), Bruising (47), Pain (41), Redness (37), Discoloration (21) | NR |
| NIKOLIS et al. (2021) | | Nasolabial folds | F: 10 | 33-72 | 5 | 24 | Ecchymosis (3), Edema (4), Erythema (3), Pain (5), Pruritus (2), Bumpiness (4), Discoloration (2), Headache (1) | Spontaneous resolution |
| HILTON et al. (2022) | | Lips | F: 40 | 23-65 | 40 | 133 | Edema/Swelling (39), Erythema (22), Bruising (35), Pain/Tenderness (31), Pruritus (6) | NR |
| MARCUS et al. (2022) | | Chin | NR: 129 | 20-73 | 18 | 20 | Pain (8), Bruising (3), Swelling (3) Erythema (2),  Hemorrhage (2), Nodule (2) | Nodule: hyaluronidase |
| DAVID et al. (2023) | | Lips, marionette lines and nasolabial folds | F: 144  M: 9 | 27-77 | 74 | 157 | Edema (53), Hematoma (37), Erythema (15), Irregularities At Palpation (26), Pain (26) | NR |
| EHLINGER-DAVID et al. (2023) | | Lips/ perioral area | F: 93  M: 7 | 18-36 | 54 | 81 | Swelling (23), Hematoma (18), Pain (12), Irregularities At Palpation (11), Erythema (5), Nodules (5), Tingling Sensation (5), Induration (2) | Spontaneous resolution |
| FENG et al. (2023) | | Nasolabial folds | F: 40 | 24-54 | NR | 46 | Swelling (7), Erythema (1), Numb (2), Sore (5), Ecchymosis (1), Foreign Body Sensation (7), Firmness (21), Hyperpigmentation (2) | NR |
| INCE et al. (2024) | | Lips | NR: 22 | 21-34 | 3 | 3 | Ecchymosis (2), Hematoma (1) | NR |
| LI et al. (2023a) | | Nasolabial folds | NR: 95 | 30-65 | NR | 17 | Erythema (5), Lumps (2), Pigmentation (2), Swelling (8) | NR |
| LI et al. (2023b) | | Nasolabial folds | F: 196  M: 9 | NR | 99 | 64 | Nodule (31), Bruising (27), Pruritus (3), Rash (1), Device Dislocation (2) | NR |
| XIE et al. (2023) | | Nasolabial folds | F: 188  M: 18 | NR | 4 | 5 | Bruising (1), Abscess (1), Nasal Pruritus (1), Erythema (1), Therapeutic Embolization (1) | NR |
| ALIMOHAMMADI et al. (2024) | | Nasolabial folds | F: 48 | 44-64 | 48 | 610 | Swelling/Edema (152), Bruising (116), Tenderness (125), Redness (111), Pain (72), Pruritus (27), Mild Implant-Site Mass (7) | Implant-site mass: hyaluronidase or spontaneous resolution |
| GUO et al. (2024) | | Chin | F: 21  M: 4 | NR | NR | 41 | Pain (16), Itchiness (1), Lumps (10), Redness (9), Bruising (5) | NR |
| LHERITIER et al. (2024) | | Nasolabial folds | F: 38  M: 7 | 37-70 | 31 | 142 | Redness (65), Lumps/Bumps (45), Induration/ Firmness (32) | NR |
| LIAO et al. (2024) | | Chin | F: 131  M: 19 | 19-55 | 79 | 236 | Tenderness to touch (41), Redness (39), Nodule (17), Pain (52), Swelling (49), Bruising (27), Itching (11) | NR |
| MASSIDDA et al. (2024) | | Lips | F: 29  M: 1 | NR | NR | 3 | Pain (1), Burning (1), Edema (1) | NR |
| NIKOLIS et al. (2024) | | Chin | F: 136  M: 4 | NR | 127 | 632 | Tenderness (121), Pain (125), Swelling (116), Bruising (97), Redness (80), Itching (76), Nodule (5), Mass (8), Headache (4) | NR |
| SAMADI et al. (2024) | | Nasolabial folds | F: 28  M: 8 | 29-65 | NR | 13 | Swelling (5), Headache (1), Pain (1), Erythema (3), Contusion (2), Nodule (1) | Pain and mild discoloration at the injection site immediately  after the injection was treated with warm compresses,  topical nitroglycerin, and oral acetaminophen 500 mg. |
| SHAO et al. (2024) | | Nasolabial folds | F: 323  M:16 | 20-64 | 296 | 991 | Swelling (275), Pain (259), Bruising (163), Erythema (163), Pruritus (128), Induration (2), Nodule (1) | Nodules and induration: massage and heat application. |
| MULLER et al. (2024) | | Lips | F: 114 | 18-73 | 67 | 142 | Pain (55), Bruising (43), Swelling (44) | NR |

NR: not reported; TRAE: treatment-related adverse event.

**Supplementary Table 5.** Treatment-related adverse events associated with aesthetic use of botulinum toxin type A in the upper face region.

|  | BoNT-A | | | | | | | |
| --- | --- | --- | --- | --- | --- | --- | --- | --- |
| Reference | | **Site** | **Sex** | **Age range**  **(years)** | **Patients with TRAEs (n)** | **TRAEs (n)** | **TRAE’s description (n)** | **Management** |
| AHN et al. (2000) | | Forehead, glabellar area, lateral canthal area, nasal dorsal area | F: 33 M: 5 | 26-56 | NR | 8 | Altered Facial Appearance (3), Swelling (2), Ecchymosis (3) | NR |
| CARRUTHERS et al. (2003) | | Glabellar lines | F: 161 M: 41 | NR | 23 | 54 | Headache (23), Erythema (6), Edema At Injection Site (1), Nausea (5), Dizziness (3), Pain In Face (4), Pain At Injection Site (4), Paresthesia (4), Infection (4) | NR |
| ASCHER et al. (2004) | | Glabellar lines | F: 97 M: 5 | NR | 7 | 7 | Forehead Rigidity (1), Headache (2), Migraine (1), Vertigo (1), Forehead Muscle Spasm (1), Forehead Ecchymosis (1) | NR |
| LOWE et al. (2005) | | Crow’s feet | F: 115 M: 15 | 27-64 | 32 | 21 | Bruising (12), Headache (9) | NR |
| MOY et al. (2009) | | Glabellar lines | F: 1084 M: 116 | 21-80 | 432 | 547 | Eye Disorders (155), Eyelid Ptosis (55), Injection Site Pain (97), Bruising (72), Headache (168) | NR |
| KERSCHER et al. (2015) | | Glabellar lines, forehead, lateral canthal area | F: 94 M: 11 | NR | 65 | 32 | Headache (24), Hematoma (4), Eyelid Ptosis (2), Dry Eyes (2) | NR |
| ZHANG et al. 2020 | | Forehead | F: 341 M: 13 | 27-61 | NR | 218 | Slight Stiffness (196), Bruising (22) | Spontaneous resolution |
| COX et al. (2023) | | Glabellar lines | F: 667  M: 70 | 21-81 | 100 | 95 | Headache (79), Ptosis (12), Vision Blurred (2), Diplopia (1), Muscle Twitching (1) | NR |
| DOVER et al. (2023) | | Glabellar, forehead, and lateral canthal lines | F: 40  M: 8 | 23-84 | 10 | 10 | Injection Site Erythema (7), Facial Discomfort (2), Headache (1) | NR |
| SOLISH et al. (2023) | | Glabellar lines | F: 2465  M: 320 | 21-86 | 997 | 424 | Headache (128), Injection Site Pain (96), Erythema (69), Injection Site Edema (61), Edema (42), Injection Site Pruritus (17), Facial Paresis (11) | NR |
| CHADHA et al. (2024) | | Glabellar lines | F: 150 | 23-64 | 18 | 15 | Pain (11), Headache (2), Tension Headache (2) | NR |
| FAGIEN et al. (2024) | | Glabellar lines | F: 145  M: 9 | 20-69 | 26 | 8 | Headache (5), Eyelid Ptosis (1), Facial Discomfort (1), Presyncope (1) | NR |
| HAN et al. (2024) | | Glabellar lines | F: 195  M: 54 | 22-60 | 7 | 8 | Eyelid Sensory Disorder (3), Injection Site Reaction (2), Erythema (1), Pruritus (2) | NR |

NR: not reported; TRAE: treatment-related adverse event.

**Supplementary Table 6.** Treatment-related adverse events associated with aesthetic non-surgical facelift using absorbable threads.

| NON-SURGICAL FACELIFT WITH ABSORBABLE THREADS | | | | | | |
| --- | --- | --- | --- | --- | --- | --- |
| Reference | **Sex** | **Age range**  **(years)** | **Patients with**  **TRAEs (n)** | **TRAEs (n)** | **TRAE’s description (n)** | **Management** |
| SUH et al. (2015) | F: 27  M:4 | NR | NR | 59 | Bruising (29), Swelling (28), Asymmetry (2) | All had spontaneous resolution |
| KANG et al. (2017) | F:38  M:1 | 31-70 | 6 | 6 | Dimpling (2), Bruising (1), Asymmetry (1), Extrusion (1), Contour Irregularity (1) | Dimpling: massage and subcision. |
| BERTOSSI et al. (2019) | F: 136  M:24 | 19-65 | 55 | 115 | Pain (52), Mild Intraoperative Bleeding And Hematoma (8), Extrusion Of The Thread (18), Erythema (15), Skin Dimpling (10), Infection (10), Facial Stiffness (2) | Extrusion of the thread: removal thread. Erythema: spontaneous resolution.  Skin dimpling: manual massage.  Infection: removal of the thread.  Facial stiffness: spontaneous resolution. |
| CHOI et al. (2020) | F:173  M:6 | NR | NR | 9 | Ecchymosis (3), Infection (1), Thread Extrusion (2), Skin Dimpling (3) | All had spontaneous resolution |
| UNAL et al. (2021) | F: 33  M:5 | NR | 4 | 4 | Infection (2), Granuloma Formation (2) | Infection: antibiotic therapy.  Granuloma: corticosteroid therapy. |
| SAHAN et al. (2023) | F: 50 | 22-56 | 9 | 16 | Ecchymosis (4), Edema (3), Erythema (2), Dimpling (2), Pain (5) | Ecchymosis and edema: cold compresses (without firm pressure).  Pain: Nonsteroid anti-inflammatory  drugs for several days.  Erythema: topical antibiotics for 1 week. Dimpling: follow-up (spontaneous resolution). |
| SINGH et al. (2023) | F: 42  M: 8 | 28-72 | NR | 87 | Pain (35), Bruising (24), Edema (15), Puckering (9), Nodule Formation (2), Thread Protrusion (2) | Thread  protrusion which occurred within the first week of the procedure was  treated with excision of protruding thread. Two patients with nodule  formation were treated with 7 days of antibiotics, upon which  it resolved. |
| LIAO & LIAO (2024) | F: 40  M: 3 | NR | NR | 72 | Pain (24), Swelling (21), Bruising (17), Irregularities (9), Thread Extrusion (1) | NR |

NR: not reported; TRAE: treatment-related adverse event.
