# Supplementary material for: Adverse Events in Nonsurgical Facial Aesthetic Procedures: A Systematic Review and Meta‐Analysis
Source: Oral Dis. 2025 Oct 5;32(2):384–94. doi: 10.1111/odi.70109 (PMC13077022; doi:10.1111/odi.70109)
Supplement: Supplementary file 6 — Table S7: Risk of bias reviewers' summary judgments about each checklist item presented as percentages according to the randomized controlled trial studies. Table S8: Risk of bias reviewers' summary judgments about each checklist item presented as percentages according to the non‐randomized controlled study. Table S9: Risk of bias reviewers' summary judgments about each checklist item presented as percentages according to the cohort study. [file ODI-32-384-s002.docx]

**Supplementary Table S7.** Risk of bias reviewers' summary judgments about each checklist item presented as percentages according to the randomized controlled trial studies.

| Nº | Author | Year | 1. | 2. | 3. | 4. | 5. | 6. | 7. | 8. | 9. | 10. | 11. | 12. | 13. | Overall rating | % |
| --- | --- | --- | --- | --- | --- | --- | --- | --- | --- | --- | --- | --- | --- | --- | --- | --- | --- |
| 1 | **Carruthers et al.** | **2003** | **Y** | **Y** | **Y** | **Y** | **Uncl** | **Uncl** | **Y** | **Y** | **Y** | **Y** | **Y** | **Y** | **Y** | **11** | **84.6** |
| 2 | **Ascher et al.** | **2004** | **Y** | **Y** | **Y** | **Y** | **Y** | **Y** | **Y** | **Y** | **Y** | **Y** | **Y** | **Y** | **Y** | **13** | **100** |
| 3 | **Lowe et al.** | **2005** | **Y** | **Y** | **Y** | **Y** | **Uncl** | **Uncl** | **Y** | **Y** | **Y** | **Y** | **Y** | **Y** | **Y** | **11** | **84.6** |
| 4 | **Kerscher et al.** | **2015** | **Y** | **Y** | **Y** | **Y** | **Y** | **Y** | **Y** | **Y** | **Y** | **Y** | **Y** | **Y** | **Y** | **13** | **100** |
| 5 | **Nikolis et al.** | **2021** | **Y** | **Y** | **Y** | **Y** | **N** | **Y** | **Y** | **Y** | **Y** | **Y** | **Y** | **Y** | **Y** | **12** | **92.3** |
| 6 | **Hilton et al.** | **2022** | **Y** | **Y** | **Y** | **Y** | **N** | **Y** | **Y** | **Y** | **Y** | **Y** | **Y** | **Y** | **Y** | **12** | **92.3** |
| 7 | **Marcus et al.** | **2022** | **Y** | **N** | **Y** | **N** | **N** | **Y** | **Y** | **Y** | **Y** | **Y** | **Y** | **Y** | **Y** | **11** | **84.6** |
| 8 | **Cox et al.** | **2023** | **Y** | **Y** | **Y** | **Y** | **N** | **Uncl** | **Y** | **Uncl** | **Y** | **Y** | **Y** | **Y** | **Y** | **10** | **76.9** |
| 9 | **Feng et al.** | **2023** | **Y** | **N** | **Y** | **N** | **N** | **Y** | **Y** | **Y** | **Y** | **Y** | **Y** | **Y** | **Y** | **10** | **76.9** |
| 10 | **Li et al.** | **2023a** | **Y** | **Y** | **Y** | **Y** | **N** | **Y** | **Y** | **Uncl** | **Y** | **Y** | **Y** | **Y** | **Y** | **11** | **84.6** |
| 11 | **Li et al.** | **2023b** | **Y** | **Y** | **Y** | **Y** | **N** | **Y** | **Y** | **Y** | **Y** | **Y** | **Y** | **Y** | **Y** | **12** | **92.3** |
| 12 | **Solish et al.** | **2023** | **Y** | **Y** | **Y** | **Y** | **N** | **Y** | **Y** | **Y** | **Y** | **Y** | **Y** | **Y** | **Y** | **12** | **92.3** |
| 13 | **Xie et al.** | **2023** | **Y** | **Y** | **Y** | **Y** | **N** | **Y** | **Y** | **Y** | **Y** | **Y** | **Y** | **Y** | **Y** | **12** | **92.3** |
| 14 | **Alimohammadi et al.** | **2024** | **Y** | **Y** | **Y** | **Y** | **N** | **Y** | **Y** | **Y** | **Y** | **Y** | **Y** | **Y** | **Y** | **12** | **92.3** |
| 15 | **Chadha et al.** | **2024** | **Y** | **Y** | **Y** | **N** | **N** | **N** | **Y** | **Y** | **Y** | **Y** | **Y** | **Y** | **Y** | **11** | **84.6** |
| 16 | **Fagien et al.** | **2024** | **Y** | **Y** | **Y** | **Y** | **N** | **Y** | **Y** | **Y** | **Y** | **Y** | **Y** | **Y** | **Y** | **12** | **92.3** |
| 17 | **Guo et al.** | **2024** | **Y** | **N** | **Y** | **N** | **N** | **Y** | **Y** | **Y** | **Y** | **Y** | **Y** | **Y** | **Y** | **10** | **76.9** |
| 18 | **Liao & Liao** | **2024** | **Y** | **N** | **Y** | **Y** | **N** | **Y** | **Y** | **Y** | **Y** | **Y** | **Y** | **Y** | **Y** | **11** | **84.6** |
| 19 | **Lheritier et al.** | **2024** | **Y** | **Y** | **Y** | **Y** | **N** | **Y** | **Y** | **Y** | **Y** | **Y** | **Y** | **Y** | **Y** | **12** | **92.3** |
| 20 | **Muller et al.** | **2024** | **Y** | **N** | **Y** | **N** | **N** | **Y** | **Y** | **Y** | **Y** | **Y** | **Y** | **Y** | **Y** | **10** | **76.9** |
| 21 | **Nikolis et al.** | **2024** | **Y** | **N** | **Y** | **N** | **N** | **Y** | **Y** | **Y** | **Y** | **Y** | **Y** | **Y** | **Y** | **10** | **76.9** |
| 22 | **Shao et al.** | **2024** | **Y** | **Y** | **Y** | **Y** | **Y** | **Y** | **Y** | **Y** | **Y** | **Y** | **Y** | **Y** | **Y** | **13** | **100** |
|  | **%** |  | **100** | **72.7** | **100** | **72.7** | **15** | **94.7** | **100** | **100** | **100** | **100** | **100** | **100** | **100** |  |  |

1.Was true randomization used for assignment of participants to treatment groups?

2.Was allocation to treatment groups concealed?

3.Were treatment groups similar at the baseline?

4.Were participants blind to treatment assignment?

5.Were those delivering treatment blind to treatment assignment?

6.Were outcomes assessors blind to treatment assignment?

7.Were treatment groups treated identically other than the intervention of interest?

8.Was follow up complete and if N t, were differences between groups in terms of their follow up adequately described and analyzed?

9.Were participants analyzed in the groups to which they were randomized?

10.Were outcomes measured in the same way for treatment groups?

11.Were outcomes measured in a reliable way?

12.Was appropriate statistical analysis used?

13.Was the trial design appropriate, and any deviations from the standard RCT design (individual randomization, parallel groups) accounted for in the conduct and analysis of the trial?

**Supplementary Table S8.** Risk of bias reviewers' summary judgments about each checklist item presented as percentages according to the non-randomized controlled study.

| Nº | Author | Year | 1. | 2. | 3. | 4. | 5. | 6. | 7. | 8. | 9. | Overall rating | % |
| --- | --- | --- | --- | --- | --- | --- | --- | --- | --- | --- | --- | --- | --- |
| 1 | **Moy et al.** | **2019** | **Y** | **Y** | **N** | **N** | **Y** | **N** | **Y** | **Y** | **N** | **5** | **55.6** |
| 2 | **Yazdanparast et al.** | **2017** | **Y** | **NA** | **NA** | **N** | **Y** | **Y** | **NA** | **Y** | **Y** | **5** | **83.3** |
| 3 | **Ehlinger-David et al.** | **2022** | **Y** | **Y** | **Y** | **N** | **Y** | **Y** | **Y** | **Y** | **Y** | **8** | **88.9** |
| 4 | **David et al.** | **2023** | **Y** | **Y** | **Y** | **N** | **Y** | **Y** | **Y** | **Y** | **Y** | **8** | **88.9** |
| 5 | **Dover et al.** | **2023** | **Y** | **Y** | **Y** | **N** | **Y** | **N** | **Y** | **Y** | **Y** | **7** | **77.8** |
| 6 | **Han et al.** | **2024** | **Y** | **Y** | **Y** | **N** | **Y** | **N** | **Y** | **Y** | **Y** | **7** | **77.8** |
| 7 | **Liao et al.** | **2024** | **Y** | **Y** | **Y** | **Y** | **Y** | **Uncl** | **Y** | **Y** | **Y** | **8** | **77.8** |
| 8 | **Massidda et al.** | **2024** | **Y** | **Y** | **Y** | **N** | **Y** | **Y** | **Y** | **Y** | **Y** | **8** | **88.9** |
| 9 | **Samadi et al.** | **2024** | **Y** | **Y** | **Y** | **N** | **Y** | **Y** | **Y** | **Y** | **Y** | **8** | **88.9** |
|  | **%** |  | **100** | **100** | **87.5** | **11.1** | **100** | **62.5** | **100** | **100** | **88.9** |  |  |

1. Is it clear in the study what is the ‘cause’ and what is the ‘effect’ (i.e. there is N confusion about which variable comes first)?

2. Were the participants included in any comparisons similar?

3. Were the participants included in any comparisons receiving similar treatment/care, other than the exposure or intervention of interest?

4. Was there a control group?

5. Were there multiple measurements of the outcome both pre and post the intervention/exposure?

6. Was follow up complete and if N t, were differences between groups in terms of their follow up adequately described and analyzed?

7. Were the outcomes of participants included in any comparisons measured in the same way?

8. Were outcomes measured in a reliable way?

9. Was appropriate statistical analysis used?

| Nº | Author | Year | 1 | 2 | 3 | 4 | 5 | 6 | 7 | 8 | 9 | 10 | 11 | Overall rating | % |  |
| --- | --- | --- | --- | --- | --- | --- | --- | --- | --- | --- | --- | --- | --- | --- | --- | --- |
| 3 | **Ahn et al.** | **2000** | **NA** | **NA** | **Y** | **N** | **N** | **Y** | **N** | **Y** | **N** | **N** | **N** | **3** | **33.3** |  |
| 2 | **Suh et al.** | **2015** | **NA** | **NA** | **Y** | **N** | **N** | **Y** | **N** | **Y** | **Y** | **N** | **N** | **4** | **44.4** |  |
| 6 | **Kang et al.** | **2017** | **NA** | **NA** | **Y** | **N** | **N** | **Y** | **Y** | **Y** | **Y** | **NA** | **Y** | **6** | **75** |  |
| 5 | **Bertossi et al.** | **2019** | **NA** | **NA** | **Y** | **N** | **N** | **Y** | **Y** | **Uncl** | **N** | **N** | **N** | **3** | **33.3** |  |
| 1 | | **Taylor et al.** | **2019** | **N** | **Y** | **Y** | **N** | **N** | **Y** | **Y** | **Y** | **Uncl** | **Uncl** | **N** | **5** | **45.5** |
| 10 | **Unal et al.** | **2019** | **NA** | **NA** | **Y** | **N** | **N** | **Y** | **Y** | **Y** | **Y** | **NA** | **Y** | **6** | **66.7** |  |
| 4 | **Choi et al.** | **2020** | **NA** | **NA** | **Y** | **N** | **N** | **Y** | **Y** | **Y** | **N** | **N** | **N** | **4** | **44.4** |  |
| 9 | **Zhang et al.** | **2020** | **NA** | **NA** | **Y** | **Y** | **Y** | **Y** | **Y** | **Y** | **Y** | **Y** | **Y** | **9** | **100** |  |
| 11 | **Ince et al.** | **2023** | **NA** | **NA** | **Y** | **N** | **N** | **Y** | **Y** | **NA** | **N** | **N** | **Y** | **4** | **50** |  |
| 7 | **Sahan et al.** | **2023** | **NA** | **NA** | **Y** | **N** | **N** | **Y** | **Y** | **Y** | **N** | **N** | **Y** | **5** | **55.6** |  |
| 8 | **Singh et al.** | **2023** | **NA** | **NA** | **Y** | **N** | **N** | **Y** | **Y** | **Y** | **Y** | **NA** | **Y** | **6** | **75** |  |
|  | **%** |  | **0** | **100** | **100** | **9.1** | **9.1** | **100** | **81.8** | **100** | **50** | **14.3** | **50** |  |  |  |

**Supplementary Table S9.** Risk of bias reviewers' summary judgments about each checklist item presented as percentages according to the cohort study.

1.Were the two groups similar and recruited from the same population?

2.Were the exposures measured similarly to assign people to both exposed and unexposed groups?

3.Was the exposure measured in a valid and reliable way?

4.Were confounding factors identified?

5.Were strategies to deal with confounding factors stated?

6.Were the groups/participants free of the outcome at the start of the study (or at the moment of exposure)?

7.Were the outcomes measured in a valid and reliable way?

8.Was the follow up time reported and sufficient to be long eN ugh for outcomes to occur?

9.Was follow up complete, and if N t, were the reasons to loss to follow up described and explored?

10.Were strategies to address incomplete follow up utilized?

11. Was appropriate statistical analysis used?
