# Supplementary material for: Adverse Events in Nonsurgical Facial Aesthetic Procedures: A Systematic Review and Meta‐Analysis
Source: Oral Dis. 2025 Oct 5;32(2):384–94. doi: 10.1111/odi.70109 (PMC13077022; doi:10.1111/odi.70109)
Supplement: Supplementary file 7 — Table S10: General characteristics of aesthetic procedures using HA fillers in the lips, lower face and nasolabial folds. Table S11: General characteristics of aesthetic BoNT‐A injections in the upper face region (procerus, forehead, and lateral canthal region). Table S12: General characteristics of aesthetic nonsurgical facelift procedures using absorbable threads. [file ODI-32-384-s004.docx]

| Supplementary Table S10. General characteristics of aesthetic procedures using HA fillers in the lips, lower face and nasolabial folds. | | | | | | | | | | |
| --- | --- | --- | --- | --- | --- | --- | --- | --- | --- | --- |
| HA FILLERS | | | | | | | | | | |
| Reference | **Study design** | **Patients (n)** | **Procedures (n)** | **Follow-up** | **Patients with TRAEs (n)** | **TRAEs (n)** | **Intensity** | **Permanent sequela** | **Time to appear** | **Time to healing** |
| YAZDANPARAST et al. (2017) | Non-Randomized Controlled Trial | 10 | NR | 24 weeks | 10 | 17 | Mild- Moderate(17) | None | NR | Pain/bruising: 1-5 days |
| TAYLOR et al. (2019) | Prospective cohort study | 72 | 112 | 9-12 months | 59 | 352 | Mild (165), Moderate(123); Severe: (64) | NR | NR | NR |
| NIKOLIS et al. (2021) | Randomized Controlled Trial | 10 | NR | 1 month | 5 | 24 | Mild-moderate (24) | None | NR | Ecchymosis: 3-7 days; Edema: 2-3 days; Erythema: 2-3 days; Pain: 1-3 days; Pruritus: 1-2 days; Bumpiness: 4-6 days; Discoloration: 2-3 days; Headache:1 day. |
| HILTON et al. (2022) | Randomized Controlled Trial | 40 | 40 | 6 months | 40 | 133 | Mild (94), Moderate (34), Severe (5) | None | NR | NR |
| MARCUS et al. (2022) | Randomized Controlled Trial | 129 | 184 | 48 weeks | 18 | 20 | Mild/moderate (20) | None | NR | Nodule: 74-122 days |
| DAVID et al. (2023) | Non-Randomized Controlled Trial | 153 | 153 | 18 months | 74 | 157 | NR | NR | NR | Most of these local reactions were resolved within 14  days |
| EHLINGER-DAVID et al. (2023) | Non-Randomized Controlled Trial | 100 | 100 | 18 months | 54 | 81 | NR | None | NR | Several days/weeks. |
| FENG et al. (2023) | Randomized Controlled Trial | 40 | NR | 6 months | NR | 46 | Mild-Moderate (46) | None | NR | All symptoms subsided within two weeks |
| INCE et al. (2024) | Retrospective cohort study | 22 | NR | NR | 3 | 3 | NR | NR | NR | NR |
| LI et al. (2023a) | Randomized Controlled Trial | 95 | NR | 12 months | NR | 17 | NR | NR | NR | NR |
| LI et al. (2023b) | Randomized Controlled Trial | 205 | NR | 12 months | 52 | 64 | Mild (64) | NR | NR | All treatment-related were resolved before the end of the study |
| XIE et al. (2023) | Randomized Controlled Trial | 206 | NR | 18 months | 4 | 5 | Mild-Moderate (5) | None | NR | NR |
| ALIMOHAMMADI et al. (2024) | Randomized Controlled Trial | 48 | 85 | 6 months | 48 | 610 | Mild (446), Moderate (146), Severe (11),  NR (7) | None | 14  days after treatment | 1-4 days |
| GUO et al. (2024) | Randomized Controlled Trial | 25 | 28 | 9.4 months | NR | 41 | Mild (41) | None | NR | NR |
| LHERITIER et al. (2024) | Randomized Controlled Trial | 45 | NR | 9 months | 31 | 142 | Mild (120), Moderate (22) | NR | NR | Self-resolution before the end of the study |
| LIAO et al. (2024) | Randomized Controlled Trial | 150 | 210 | 12 months | 79 | 236 | NR | NR | NR | NR |
| MASSIDDA et al. (2024) | Non-Randomized Controlled Trial | 30 | NR | 6 months | NR | 3 | Mild (3) | None | NR | NR |
| NIKOLIS et al. (2024) | Randomized Controlled Trial | 140 | NR | 12 months | 127 | 632 | NR | None | NR | NR |
| SAMADI et al. (2024) | Non-Randomized Controlled Trial | 36 | NR | 6 months | NR | 13 | Mild (9), Moderate (3), Severe (1) | None | 2 days | 2 weeks |
| SHAO et al. (2024) | Randomized Controlled Trial | 339 | NR | 12 months | 296 | 991 | NR | None | NR | Nodules and induration: 2 weeks |
| MULLER et al. (2024) | Randomized Controlled Trial | 114 | 168 | 18 months | 67 | 142 | NR | NR | NR | NR |

NR: not reported. TRAE: treatment-related adverse event.

| Supplementary Table S11. General characteristics of aesthetic BoNT-A injections in the upper face region (procerus, forehead, and lateral canthal region).  BoNT-A | | | | | | | | | | | |
| --- | --- | --- | --- | --- | --- | --- | --- | --- | --- | --- | --- |
| Reference | **Study design** | **Patients (n)** | **Procedures (n)** | **Material** | **Follow-up** | **Patients with TRAEs (n)** | **TRAEs (n)** | **Intensity** | **Permanent sequela** | **Time to appear** | **Time to healing** |
| AHN et al. (2000) | Retrospective cohort study | 38 | 59 | Botulinum Toxin Type A (n=38) | 0-4 months | NR | 8 | Mild (2), NR (6) | None | NR | NR |
| CARRUTHERS et al. (2003) | Randomized Controlled Trial | 202 | NR | Botulinum Toxin Type A (n=202) | 0-4 months | 23 | 54 | NR | None | NR | Headache: some hours |
| ASCHER et al. (2004) | Randomized Controlled Trial | 102 | 102 | Botulinum Toxin Type A (n=102) | 3-12 months | 7 | 7 | NR | None | NR | Altered facial appearance and swelling: 2-3 weeks; Ecchymosis: up to 1 month. |
| LOWE et al. (2005) | Randomized Controlled Trial | 130 | 130 | Botulinum Toxin Type A  (n=130) | 12 months | 32 | 21 | NR | NR | NR | NR |
| MOY et al. (2009) | Non-Randomized Controlled Trial | 1200 | 4214 | OnabotulinumtoxinA A (n=1,200) | 13 months | 432 | 547 | NR | NR | NR | NR |
| KERSCHER et al. (2015) | Randomized Controlled Trial | 105 | NR | IncobotulinumtoxinA  (n=105) | 12 months | 65 | 32 | NR | NR | NR | NR |
| ZHANG et al. (2020) | Retrospective cohort study | 354 | 667 | Botulinum Toxin Type A (n=354) | 6 months | NR | 218 | NR | NR | NR | NR |
| COX et al. (2023) | Randomized Controlled Trial | 737 | NR | PrabotulinumtoxinA (n=737) | 5 months | 100 | 95 | NR | NR | NR | NR |
| DOVER et al. (2023) | Non-Randomized Controlled Trial | 48 | 48 | DaxibotulinumtoxinA (n=48) | 36 weeks | 10 | 10 | NR | NR | NR | NR |
| SOLISH et al (2023) | Randomized Controlled Trial | 2785 | 2785 | DaxibotulinumtoxinA (n=2,785) | 8.33 months | 997 | 424 | NR | NR | NR | NR |
| CHADHA et al. (2024) | Randomized Controlled Trial | 150 | NR | AbobotulinumtoxinA (n=150) | 1-6 months | 18 | 15 | Mild (13), Moderate (1), Severe (1) | None | On the day of treatment | 4 days |
| FAGIEN et al. (2024) | Randomized Controlled Trial | 154 | 154 | PrabotulinumtoxinA-xvfs (n=104)  OnabotulinumtoxinA A (n=50) | 5.17 – 6.3 months | 26 | 8 | Mild (8) | None | Eyelid ptosis: 15 days | Eyelid ptosis: 67 days |
| HAN et al. (2024) | Non-Randomized Controlled Trial | 249 | 712 | Botulinum Toxin Type A- MBA-P01 (n=249) | 4.77 months | 7 | 8 | NR | NR | NR | NR |

NR: not reported. TRAE: treatment-related adverse event.

| Supplementary Table S12. General characteristics of aesthetic non-surgical facelift procedures using absorbable threads. | | | | | | | | | | | |
| --- | --- | --- | --- | --- | --- | --- | --- | --- | --- | --- | --- |
| NON-SURGICAL FACELIFT WITH ABSORBABLE THREADS | | | | | | | | | | | |
| Reference | **Study design** | **Patients (n)** | **Procedures (n)** | **Material** | **Follow-up** | **Patients with TRAEs (n)** | **TRAEs (n)** | **Intensity** | **Permanent sequela** | **Time to appear** | **Time to healing** |
| SUH et al. (2015) | Retrospective cohort study | 31 | 31 | Polydioxanone monofilament thread (n=31) | 6 months | NR | 59 | NR | None | NR | 2 weeks |
| KANG et al. (2017) | Retrospective cohort study | 39 | 39 | Polydioxanone monofilament thread (n=39) | 6 months | 6 | 6 | NR | NR | NR | NR |
| BERTOSSI et al. (2019) | Retrospective cohort study | 160 | NR | Polydioxanone monofilament thread (n=160) | 0-14 months | 55 | 115 | NR | NR | NR | NR |
| CHOI et al. (2020) | Retrospective cohort study | 179 | 179 | Polydioxanone monofilament thread (n=179) | 22.3 months (mean) | NR | 9 | Mild- Moderate (9) | NR | NR | NR |
| UNAL et al. (2021) | Retrospective cohort study | 38 | NR | Polydioxanone monofilament thread (n=38) | 11-44 months | 4 | 4 | NR | NR | NR | NR |
| SAHAN et al. (2023) | Retrospective cohort study | 50 | 50 | Polydioxanone thread (n=50) | 13-23 months | 9 | 16 | NR | None | NR | Skin dimpling: 10 days;  Erythema: 7 days. |
| SINGH et al. (2023) | Retrospective cohort study | 50 | 50 | Poly-lactic-caprolactone thread (n=50) | 3-6 months | NR | 87 | NR | NR | NR | NR |
| LIAO et al. (2024) | Non-Randomized Controlled Trial | 43 | NR | Polydioxanone thread (n=50) | 12 months | NR | 72 | NR | NR | NR | NR |
|  |  |  |  |  |  |  |  |  |  |  |  |

NR: not reported. TRAE: treatment-related adverse event.
