# Supplementary material for: Adverse Events in Nonsurgical Facial Aesthetic Procedures: A Systematic Review and Meta‐Analysis
Source: Oral Dis. 2025 Oct 5;32(2):384–94. doi: 10.1111/odi.70109 (PMC13077022; doi:10.1111/odi.70109)
Supplement: Supplementary file 8 — Table S13: Grading of Recommendation, Assessment, Development, and Evaluation (GRADE) evidence profile. [file ODI-32-384-s009.docx]

**Supplementary Table S13. Grading of Recommendation, Assessment, Development, and Evaluation (GRADE) evidence profile.**

| **Certainty assessment** | | | | | | | **№ of patients** | | **Effect** | | **Certainty** | **Importance** |
| --- | --- | --- | --- | --- | --- | --- | --- | --- | --- | --- | --- | --- |
| **№ of studies** | **Study design** | **Risk of bias** | **Inconsistency** | **Indirectness** | **Imprecision** | **Other considerations** | **Total** | **Events** | **Relative (95% CI)** | **Absolute (95% CI)** |  |  |
| **Prevalence of TRAEs after procedures with HA fillers in lips, nasolabial folds, chin and marionette lines** | | | | | | | | | | | | |
| 16 | randomised trials | not serious | serious^b^ | not serious | very serious^c^ | none | 1783 | 1014 | - | mean **0.63 SD higher** (0.35 higher to 0.84 higher) | ⨁◯◯◯ Very low |  |
| **Prevalence of TRAEs after upper face BoNT-A** | | | | | | | | | | | | |
| 11 | randomised trials | not serious | serious^b^ | not serious | very serious^c^ | none | 5862 | 1717 | - | mean **0.18 SD higher** (0.1 higher to 0.32 higher) | ⨁◯◯◯ Very low |  |
| **Prevalence of TRAEs after non-surgical facelift with absorbable threads** | | | | | | | | | | | | |
| 4 | non-randomised studies | serious^a^ | serious^b^ | not serious | very serious^c^ | none | 287 | 74 | - | mean **0.2 SD higher** (0.08 higher to 0.41 higher) | ⨁◯◯◯ Very low |  |

**CI:** confidence interval

**Explanations**

a. Studies with moderate and high risk of bias

b. Considerable heterogeneity (I^2^>75%)

c. Large confidence interval
