# Supplementary material for: Adverse Events in Nonsurgical Facial Aesthetic Procedures: A Systematic Review and Meta‐Analysis
Source: Oral Dis. 2025 Oct 5;32(2):384–94. doi: 10.1111/odi.70109 (PMC13077022; doi:10.1111/odi.70109)
Supplement: Supplementary file 9 — Table S14: Checklist for Treatment‐Related Adverse Event (TRAE) Reporting in Minimally Invasive Aesthetic Treatments. [file ODI-32-384-s010.docx]

**Supplementary Table S14.** Checklist for Treatment-Related Adverse Event (TRAE) Reporting in Minimally Invasive Aesthetic Treatments.

**Definition of TRAE adopted (e.g., any undesirable sign, symptom, or disease with plausible causal relationship to the procedure).**

| 1. **Population, Heath Care Provider and Procedures** |
| --- |
| - - Total number of patients in the treatment and placebo groups. |
| - - Average number of procedures per patient. |
| - - Total number of procedures performed per group. |
| - - Follow-up period for AE monitoring. |
| - - Category and training of the health care provider performing the procedure (e.g., physician, dentist, nurse; specify level of training/experience). |
| 1. **Treatment-Related Adverse Events** |
| - - Total number of TRAEs reported per group (treatment and placebo). |
| - - Detailed clinical description of each adverse event. |
| - - Whether the adverse event was related to the treatment. |
| - - Time of onset of signs or symptoms after the procedure (specify exact interval; classify as immediate <24h, early 24h–4 weeks, delayed >4 weeks whenever possible). |
| - - Duration of the adverse event. |
| - - Whether medical intervention was required. |
| - - Type of intervention performed and outcome (resolution, persistence, recurrence, etc.). |
| - - Specify whether each AE was transient or permanent (if absent, explicitly state that no permanent sequelae were observed).   - Report whether the AE was objectively assessed (by clinician) or subjectively reported (by patient). |
| 1. **Severity of Events** |
| - - Severity grading according to the **Common Terminology Criteria for Adverse Events (CTCAE v5.0)**, U.S. Department of Health and Human Services – National Cancer Institute: |
| **Grade 1 – Mild**: Asymptomatic or mild symptoms; only clinical or diagnostic observations; no intervention indicated. |
| **Grade 2 – Moderate**: Minimal, local, or noninvasive intervention indicated; limits age-appropriate instrumental activities of daily living (instrumental ADL). |
| **Grade 3 – Severe**: Medically significant but not immediately life-threatening; hospitalization or prolonged hospitalization indicated; disabling; limits self-care activities of daily living (self-care ADL). |
| U.S. Department of Health and Human Services. Common Terminology Criteria for Adverse Events (CTCAE) Version 5.0. National Institutes of Health, National Cancer Institute; 2017. Available from: https://ctep.cancer.gov/protocoldevelopment/electronic_applications/ctc.htm |
