# Supplementary material for: Adverse Events in Nonsurgical Facial Aesthetic Procedures: A Systematic Review and Meta‐Analysis
Source: Oral Dis. 2025 Oct 5;32(2):384–94. doi: 10.1111/odi.70109 (PMC13077022; doi:10.1111/odi.70109)
Supplement: Supplementary file 10 — Appendix S1: List of references of articles included in this systematic review. [file ODI-32-384-s001.docx]

**SUPPLEMENTARY MATERIAL** List of references of articles included in this systematic review.

Ahn, K. Y., Park, M. Y., Park, D. H., & Han, D. G. (2000). Botulinum toxin A for the treatment of facial hyperkinetic wrinkle lines in Koreans. *Plast Reconstr Surg,* 105(2), 778-84. doi: 10.1097/00006534-200002000-00050.

Alimohammadi, M., Furman-Assaf, S., & Nilsson, J. (2024). A Prospective, Randomized, Double-Blind, Split-Face, Comparative Study to Evaluate the Efficacy and Safety of DKL23 and Juvéderm Volift for Correcting Moderate-to-Severe Nasolabial Folds. *Aesthet Surg J,* 44(11), 1218-1226. doi: 10.1093/asj/sjae133.

Ascher, B., Zakine, B., Kestemont, P., Baspeyras, M., Bougara, A., & Santini, J. (2004). A multicenter, randomized, double-blind, placebo-controlled study of efficacy and safety of 3 doses of botulinum toxin A in the treatment of glabellar lines. *J Am Acad Dermatol*, 51(2), 223-233. doi: 10.1016/j.jaad.2003.11.084. Erratum in: *J Am Acad Dermatol,* 52(1), 156, 2005.

Bertossi, D., Botti, G., Gualdi, A., Fundarò, P., Nocini, R., Pirayesh, A., & van der Lei, B. (2019). Effectiveness, Longevity, and Complications of Facelift by Barbed Suture Insertion. *Aesthet Surg J,* 39(3), 241-247. doi: 10.1093/asj/sjy042.

Carruthers, J. D., Lowe, N. J., Menter, M. A., Gibson, J., & Eadie, N. (2003). Double-blind, placebo-controlled study of the safety and efficacy of botulinum toxin type A for patients with glabellar lines. *Plast Reconstr Surg,* 112(4), 1089-98. doi: 10.1097/01.PRS.0000076504.79727.62.

Chadha, P., Gerber, P. A., Hilton, S., Molina, B., Haq, S., Partridge, J., … Prygova, I. (2024). Ready-to-use abobotulinumtoxinA solution versus powder botulinumtoxinA for treatment of glabellar lines: Investigators' and subjects' experience in a Phase IV study. *J Cosmet Dermatol,* 23(9), 2857-2866. doi: 10.1111/jocd.16359.

Choi, M. S., Byeon, H. S., & Moon, H. J. (2020). Short-term safety of facial rejuvenation using an absorbable polydioxanone monofilament thread in patients with mild-to-moderate facial skin sagging. *Archives of Aesthetic Plastic Surgery,* 26(2), 53-56.

Cox, S. E., Ascher, B., Avelar, R. L., Beer, K. R., Carruthers, J., Cartier, H., … Solish, N. (2023). PrabotulinumtoxinA for the treatment of glabellar lines in adults, 65 years of age and older: The fourth in a series of post hoc analyses of the phase III clinical study data. *J Cosmet Dermatol,* 22(6), 1745-1756. doi: 10.1111/jocd.15783.

David, M., Braccini, F., Garcia, P., Loreto, F., Benadiba, L., Gorj, M., ... Fanian F. (2023). Long-Term Efficacy and Tolerability of a Medium G' HA Filler with Tri-Hyal Technology on the Rejuvenation of the Mobile Facial Zone. Clin Cosmet Investig Dermatol, 16, 1795-1805. doi: 10.2147/CCID.S395353.

Dover, J. S., Humphrey, S. D., Lorenc, Z. P., Shamban, A., Gross, T. M., Rubio, R. G., & Vitarella, D. (2023). Treatment of Upper Facial Lines With DaxibotulinumtoxinA for Injection: Results From an Open-Label Phase 2 Study. *Dermatol Surg,* 49(1), 60-65. doi: 10.1097/DSS.0000000000003637.

Ehlinger-David, A., Gorj, M., Braccini, F., Loreto, F., Grand-Vincent, A., Garcia, P., … Fanian F. (2023). A prospective multicenter clinical trial evaluating the efficacy and safety of a hyaluronic acid-based filler with Tri-Hyal technology in the treatment of lips and the perioral area. *J Cosmet Dermatol,* 22(2), 464-472. doi: 10.1111/jocd.15169.

Fagien, S., Avelar, R. L., Cox, S. E., Joseph, J. H., Kaufman-Janette, J., & Marcus, K. A. Safety and Duration of Effect of 40-Unit PrabotulinumtoxinA-xvfs for the Treatment of Moderate to Severe Glabellar Lines in Adult Patients: A Phase II, Multicenter, Randomized, Double-Blind, Active-Controlled Trial. Aesthet Surg J. 2024;44(9):987-1000. doi: 10.1093/asj/sjae051.

Feng, G., Peng, T., Hong, W. J., Zhang, Y. L., Li, G., Zheng, W., … Luo, S. K. (2023). A Two-Center, Prospective, Randomized Controlled Trial to Evaluate the Efficacy and Safety of and Satisfaction with Different Methods of ART FILLER® UNIVERSAL Injection for Correcting Moderate to Severe Nasolabial Folds in Chinese Individuals. *Aesthetic Plast Surg,* 47(4), 1550-1559. doi: 10.1007/s00266-023-03278-2.

Guo, Y., Wang, J., Wei, W., Zhang, A., Li, Q., Tao, … Jin, P. (2024). Treatment of Chin Retrusion With Botulinum Toxin Plus Hyaluronic Acid Filler in Comparison With Hyaluronic Acid Filler Alone: A Randomized, Evaluator-Blinded, Controlled Study. *Aesthet Surg J,* 44(5), 537-544. doi: 10.1093/asj/sjad358.

Han, H. S., Kim, W. S., Lee, Y. W., Won, C. H., Lee, W., Choi, S. Y., & Kim, B. J. (2024). Long-term safety and efficacy of MBA-P01 for the treatment of glabellar lines: results from a multicenter, repeated-dose, open-label extension study. *J Dermatolog Treat,* 35(1), 2418919. doi: 10.1080/09546634.2024.2418919.

Hilton, S., Frank, K., Alfertshofer, M., & Cotofana, S. (2023). Clinical outcomes after lip injection procedures-Comparison of two hyaluronic acid gel fillers with different product properties. *J Cosmet Dermatol,* 22(1), 119-127. doi: 10.1111/jocd.15548.

Ince, B., Zuhour, M., Kadiyoran, C., Avunduk, M. C., & Dadaci, M. (2024). A Comparison Between Hyaluronic Acid Filler and Dermofat Grafts With or Without Tie-Over Dressing for Lip Augmentation. *Dermatol Surg,* 50(1), 52-58. doi: 10.1097/DSS.0000000000003995.

Kang, S. H., Byun, E. J., & Kim, H. S. (2017) Vertical Lifting: A New Optimal Thread Lifting Technique for Asians. *Dermatol Surg,* 43(10), 1263-1270. doi: 10.1097/DSS.0000000000001169.

Kerscher, M., Rzany, B., Prager, W., Turnbull, C., Trevidic, P., & Inglefield, C. (2015). Efficacy and Safety of IncobotulinumtoxinA in the Treatment of Upper Facial Lines: Results From a Randomized, Double-Blind, Placebo-Controlled, Phase III Study. *Dermatol Surg,* 41(10), 1149-57. doi: 10.1097/DSS.0000000000000450.

Lheritier, C., Converset, S., Rzany, B. J., Cartier, H., & Ascher, B. (2024). Efficacy of a New Hyaluronic Acid Dermal Filler on Nasolabial Folds Correction: A Prospective, Comparative, Double-Blinded Clinical Trial. *Dermatol Surg,* 50(8), 746-751. doi: 10.1097/DSS.0000000000004207.

Li, W., Li, B., Hofmann, M., Klein, G., & Xie, H. (2023b). A Multicenter Noninferiority Study Comparing Safety and Effectiveness of Hyaluronic Acid Fillers for Correction of Nasolabial Folds in Chinese Subjects. *Plast Reconstr Surg Glob Open,* 11(2), e4810. doi: 10.1097/GOX.0000000000004810.

Li, X. Z., Chiang, C. F., Lin, Y. H., Chen, T. M., Wang, C. H., Tzeng, Y. S., & Cui, H. Y. (2023a). Safety and efficacy of hyaluronic acid injectable filler in the treatment of nasolabial fold wrinkle: a randomized, double-blind, self-controlled clinical trial. *J Dermatolog Treat,* 34(1), 2190829. doi: 10.1080/09546634.2023.2190829.

Liao, K. L., & Liao, K. H. (2024). Study and Analysis of the Clinical Effects and Maintenance Duration of Facial Rejuvenation Treatment in Middle-Aged and Elderly Individuals through the Combined Use of Facial Hyaluronic Acid Fillers and PPDO Thread Lift. *Altern Ther Health Med,* AT10596.

Liao, Z., Ma, J., Bu, X., Zhang, Y., Wang, S., Hou, Y., … Li, B. (2025). Effectiveness and Safety of a Hyaluronic Acid Filler for Chin Augmentation and Correction of Chin Retrusion: A Multi-center, Prospective, Randomized Controlled Trial. *Aesthetic Plast Surg,* 49(4), 1046-1053. doi: 10.1007/s00266-024-04494-0.

Lowe, N. J., Ascher, B., Heckmann, M., Kumar, C., Fraczek, S., & Eadie, N. (2005). Double-blind, randomized, placebo-controlled, dose-response study of the safety and efficacy of botulinum toxin type A in subjects with crow's feet. *Dermatol Surg,* 31(3), 257-62. doi: 10.1111/j.1524-4725.2005.31070.

Marcus, K., Moradi, A., Kaufman-Janette, J., Ablon, G., Donofrio, L., Chapas, A., … George R. (2022). A Randomized Trial to Assess Effectiveness and Safety of a Hyaluronic Acid Filler for Chin Augmentation and Correction of Chin Retrusion. *Plast Reconstr Surg,* 150(6), 1240e-1248e. doi: 10.1097/PRS.0000000000009733.

Massidda, E., Ciampa, S., Iozzo, I., Emanuele, E., & Minoretti, P. (2024). Real-World Outcomes of Lip Augmentation Using a Hyaluronic Acid-Based Filler With Low 1,4-Butanediol Diglycidyl Ether Content: A Prospective, Open-Label, Multicenter, Post-marketing Study. *Cureus,* 16(2), e53513. doi: 10.7759/cureus.53513.

Moy, R., Maas, C., Monheit, G., & Huber, M.B. (2009). Long-term safety and efficacy of a new botulinum toxin type A in treating glabellar lines. *Arch Facial Plast Surg,* 11(2), 77-83. doi: 10.1001/archfacial.2009.5.

Müller, D. S., Grablowitz, D., Krames-Juerss, A., & Worseg, A. (2024). Lip Augmentation With Saypha LIPS Lidocaine: A Postmarket, Prospective, Open-Label, Randomized Clinical Study To Evaluate Its Efficacy and Short- and Long-term Safety. *Aesthet Surg J,* 45(1), 84-97. doi: 10.1093/asj/sjae149.

Nikolis, A., Enright, K. M., Öhrlund, Å., Winlöf, P., Cotofana, S. (2021). A randomized, split-face, double-blind, comparative study of the safety and efficacy of small- and large-particle hyaluronic acid fillers for the treatment of nasolabial folds. *J Cosmet Dermatol,* 20(5), 1450-1458. doi: 10.1111/jocd.13668.

Nikolis, A., Humphrey, S., Rivers, J. K., Bertucci, V., Solish, N., McGillivray, W., … Bromee, T. (2024). Effectiveness and Safety of a New Hyaluronic Acid Injectable for Augmentation and Correction of Chin Retrusion. *J Drugs Dermatol,* 23(4), 255-261. doi: 10.36849/JDD.8145.

Sahan, A., Karaosmanoglu, N., & Ozdemir Cetinkaya, P. (2023). Is it possible to obtain long-lasting results with thread lift in the brow region? Introduction of a new suspension technique and evaluation of 50 patients. *J Cosmet Dermatol,* 22(6), 1863-1869. doi: 10.1111/jocd.15658.

Samadi, A., Ahmadian, Yazdi, H., Kafi, H., Yazdanparast, T., Ahmad Nasrollahi, S., Zartab, H., & Firooz, A. (2025). Efficacy Evaluation of a Hyaluronic Acid Dermal Filler Containing Mannitol: Clinical and Aesthetic Assessment Using High-Frequency Ultrasound. *Dermatol Surg,* 51(1), 46-51. doi: 10.1097/DSS.0000000000004355.

Shao, H., Wang, L., Tang, J., Chen, L., Zhang, S., Chen, Q., …Zhao, H. (2024). A 52-week follow-up, multi-center, randomized, double-blinded comparison of efficacy and safety of two hyaluronic acid fillers for the treatment of moderate-to-severe nasolabial folds in Chinese population. *J Dermatolog Treat,* 35(1), 2378165. doi: 10.1080/09546634.2024.2378165.

Singh, S., Ballani, I., Patil, C. Y., & Kaushik, I. (2023). Retrospective real-world study of Definisse threads for jaw line reshaping in Indian patients (REDEFINE JAW study). *J Cosmet Dermatol,* 22(10), 2785-2790. doi: 10.1111/jocd.15774.

Solish, N., Burgess, C. M., Weinkle, S. H., Ablon, G., Brown, J., Kooken, K., & Rubio, R. G. (2023). Efficacy and Safety of DaxibotulinumtoxinA for Injection in the Treatment of Glabellar Lines by Age and Race: Subgroup Analysis of the SAKURA Clinical Trials. *Aesthet Surg J,* 43(2), 205-214. doi: 10.1093/asj/sjac246.

Suh, D. H., Jang, H. W., Lee, S. J., Lee, W. S., & Ryu, H. J. (2015). Outcomes of polydioxanone knotless thread lifting for facial rejuvenation. *Dermatol Surg,* 41(6), 720-725. doi: 10.1097/DSS.0000000000000368.

Taylor, S. C., Downie, J. B., Shamban, A., Few, J., Weichman, B. M., Schumacher, A., & Gallagher, C. J. (2019). Lip and Perioral Enhancement With Hyaluronic Acid Dermal Fillers in Individuals With Skin of Color. *Dermatol Surg,* 45(7), 959-967. doi: 10.1097/DSS.0000000000001842.

Unal, M., İslamoğlu, G. K., Ürün Unal, G., & Köylü, N. (2021). Experiences of barbed polydioxanone (PDO) cog thread for facial rejuvenation and our technique to prevent thread migration. *J Dermatolog Treat,* 32(2), 227-230. doi: 10.1080/09546634.2019.1640347.

Xie, Y., Wu, S., Wang, L., Mu, X., Shu, M., Hofmann, M., … Li, Q. (2023). Long-term Safety and Effectiveness of Hyaluronic Acid Fillers Correcting Nasolabial Folds in Chinese Patients. *Plast Reconstr Surg Glob Open,* 11(11), e5423. doi: 10.1097/GOX.0000000000005423.

Yazdanparast, T., Samadi, A., Hasanzadeh, H., Nasrollahi, S. A., Firooz, A., & Kashani, M. N. (2017). Assessment of the Efficacy and Safety of Hyaluronic Acid Gel Injection in the Restoration of Fullness of the Upper Lips. *J Cutan Aesthet Surg,* 10(2), 101-105. doi: 10.4103/JCAS.JCAS_115_16.

Zhang, X., Cai, L., Yang, M., Li, F., & Han, X. (2020). Botulinum Toxin to Treat Horizontal Forehead Lines: A Refined Injection Pattern Accommodating the Lower Frontalis. *Aesthet Surg J,* 40(6), 668-678. doi: 10.1093/asj/sjz174.
